# Genetics of self-reported risk-taking behaviour, trans-ethnic consistency and relevance to brain gene expression

**DOI:** 10.1038/s41398-018-0236-1

**Published:** 2018-09-04

**Authors:** Rona J. Strawbridge, Joey Ward, Laura M. Lyall, Elizabeth M. Tunbridge, Breda Cullen, Nicholas Graham, Amy Ferguson, Keira J. A. Johnston, Donald M. Lyall, Daniel Mackay, Jonathan Cavanagh, David M. Howard, Mark J. Adams, Ian Deary, Valentina Escott-Price, Michael O’Donovan, Andrew M. McIntosh, Mark E. S. Bailey, Jill P. Pell, Paul J. Harrison, Daniel J. Smith

**Affiliations:** 1 Institute of Health and Wellbeing, University of Glasgow, Glasgow, UK; 2 Department of Medicine Solna, Karolinska Institute, Stockholm, Sweden; 3 Department of Psychiatry, University of Oxford, Oxford, UK; 4 Oxford Health NHS Foundation Trust, Oxford, UK; 5 School of Life Sciences, College of Medical, Veterinary and Life Sciences, University of Glasgow, Glasgow, UK; 6 Division of Psychiatry, College of Medicine, University of Edinburgh, Edinburgh, UK; 7 Division of Psychiatry, University of Edinburgh, Royal Edinburgh Hospital, Edinburgh, EH10 5HF UK; 8 Department of Psychology, University of Edinburgh, Edinburgh, EH8 9YL UK; 9 Centre for Cognitive Ageing and Cognitive Epidemiology, University of Edinburgh, Edinburgh, EH8 9YL UK; 10 MRC Centre for Neuropsychiatric Genetics and Genomics, Cardiff University, Cardiff, UK

## Abstract

Risk-taking behaviour is an important component of several psychiatric disorders, including attention-deficit hyperactivity disorder, schizophrenia and bipolar disorder. Previously, two genetic loci have been associated with self-reported risk taking and significant genetic overlap with psychiatric disorders was identified within a subsample of UK Biobank. Using the white British participants of the full UK Biobank cohort (*n* = 83,677 risk takers versus 244,662 controls) for our primary analysis, we conducted a genome-wide association study of self-reported risk-taking behaviour. In secondary analyses, we assessed sex-specific effects, trans-ethnic heterogeneity and genetic overlap with psychiatric traits. We also investigated the impact of risk-taking-associated SNPs on both gene expression and structural brain imaging. We identified 10 independent loci for risk-taking behaviour, of which eight were novel and two replicated previous findings. In addition, we found two further sex-specific risk-taking loci. There were strong positive genetic correlations between risk-taking and attention-deficit hyperactivity disorder, bipolar disorder and schizophrenia. Index genetic variants demonstrated effects generally consistent with the discovery analysis in individuals of non-British White, South Asian, African-Caribbean or mixed ethnicity. Polygenic risk scores comprising alleles associated with increased risk taking were associated with lower white matter integrity. Genotype-specific expression pattern analyses highlighted *DPYSL5*, *CGREF1* and *C15orf59* as plausible candidate genes. Overall, our findings substantially advance our understanding of the biology of risk-taking behaviour, including the possibility of sex-specific contributions, and reveal consistency across ethnicities. We further highlight several putative novel candidate genes, which may mediate these genetic effects.

## Introduction

The Research Domain Criteria approach proposes studying traits existing in the general population (rather than categorical diagnoses) to better understand psychopathology. One such trait is risk-taking behaviour, a key component of psychiatric disorders such as attention-deficit hyperactivity disorder (ADHD)^1,2^ and bipolar disorder (BD)^3^. Risk taking is also observed in schizophrenia (SCZ), although cognitive difficulties^4,5^ may confound this relationship. Problem behaviours such as smoking and drug and alcohol misuse^6,7^ frequently co-occur with psychiatric disorders and might also be considered a consequence of risk-taking behaviour.

Previous studies have found that risk taking and impulsivity are associated with reduced grey matter volumes and/or reduced thickness in several subcortical and prefrontal regions of the brain, as well as with reduced white matter integrity^8–10^. It is therefore likely that genetic predisposition to risk-taking behaviour impacts on brain structure and function.

Previous genetic studies of risk taking^11–13^ identified two associated loci (*CADM2* (cell adhesion molecule 2) and a locus within the *HLA* (human leukocyte antigen) region) and found genetic correlations with ADHD, BD and SCZ, smoking, alcohol and drug use^13^. The full UK Biobank data release more than doubles the sample size available for genome-wide association studies (GWAS) of risk taking and allows for the investigation of sex-specific effects, the assessment of trans-ethic heterogeneity, and assessment of genetic effects on brain structures.

## Subjects and methods

### UK Biobank and primary phenotype definition

**Fig. 1:**
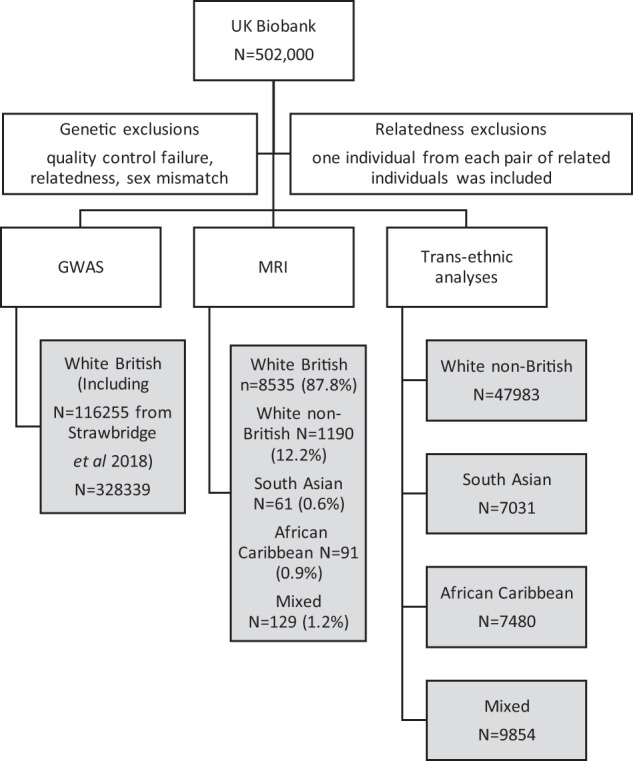
Flow diagram outlining the study design. Primary analysis included all white British participants, whereas secondary analysis included trans-ethnic assessment of the risk-taking loci

UK Biobank is a population cohort of over 0.5 million individuals recruited between 2006 and 2010^14^. Baseline assessments included extensive questionnaires on socioeconomic status, lifestyle and medical history. All participants provided informed consent. This study was carried out under the generic approval from the NHS National Research Ethics Service (Ref 16/NW/0274) and under UK Biobank applications #6553 and #17689. Genotyping, imputation and quality control procedures have been described previously^15–18^ (Supplemental Information). Self-reported risk-taking behaviour was assessed with the question: “Would you describe yourself as someone who takes risks?” (data field #2040). Individuals who responded “no” are here referred to as controls (*n* = 244,662) and those who responded “yes” are “risk takers” (*n* = 83,677). Assessment of response consistency was possible for a subset of participants (*n* = 14,551) who answered the same question at follow-up (during 2012–2013). Figure 1 demonstrates the study design.

### GWAS of self-reported risk-taking behaviour

For the GWAS, only participants of (self-reported) white British ancestry were analysed (including those analysed in our previous study^13^, Fig. 1 and Supplemental Information). Logistic regression was conducted in PLINK 1.07^19^ assuming additive allelic effects. Analyses were conducted initially in all subjects and subsequently stratified by sex, adjusting for age, genotyping chip and population structure (using eight principal components) and sex (combined analysis only). The threshold for GWAS significance was set at *p* < 5 × 10^−8^. Linkage disequilibrium score regression (LDSR)^20^ was used to estimate the risk-taking single nucleotide polymorphism (SNP) heritability (*h*^2^_SNP_, observed scale).

### Genetic correlations with related traits

Genetic correlations between risk-taking behaviour and published GWAS summary statistics of psychiatric, cognitive and behavioural traits were investigated using LDSR^20^. A small number of phenotypes for LDSR were chosen based on a priori biological and clinical knowledge, so no multiple testing correction was applied and *p* < 0.05 was considered significant (Supplemental Information). Phenotypes assessed: ADHD, post-traumatic stress disorder (PTSD), BD, SCZ, major depressive disorder (MDD), anxiety, fluid intelligence, years of education, lifetime cannabis use, ever smoking, alcohol consumption, body mass index (BMI), waist to hip ratio adjusted for BMI (WHRadjBMI), caudate and accumbens volumes.

### Genetic analysis of lead SNPs in all ethnicities

To assess whether associated loci were consistent across different ethnicities, the effects of lead SNPs on risk-taking behaviour were also assessed in individuals of self-reported south Asian (*n* = 2764 risk takers versus 4267 controls), African-Caribbean (*n* = 3139 versus 4341), white non-British (*n* = 16,169 versus 31,814) and mixed ethnicity (*n* = 3866 versus 5988) backgrounds (Fig. 1). Genetic analysis was conducted in PLINK^19^ as above. An inverse variance-weighted meta-analysis of all ethnic groups was conducted in METAL^21^ to measure heterogeneity in effects across the ethnic groups.

### Polygenic risk score analyses and magnetic resonance imaging (MRI)

The subset of UK Biobank individuals who had brain MRI data were examined to assess the impact of a polygenic risk score (PRS) for risk taking on measures of white matter integrity, total tissue volumes and volumes of selected anatomical regions of interest (ROIs) previously been implicated in risk-taking behaviour (Fig. 1, Supplemental Information). The PRS was calculated using summary statistics from a secondary GWAS in which participants with MRI data had been excluded.

### MRI analyses

Details of the MRI procedure in UK Biobank have been described previously^22^ and are presented in the Supplemental Information. In brief, brain MRI scanning was conducted at a single site, and structural T1 and DTI measures were calculated by UK Biobank, using FSL (FMRIB Software Library)^23^.

Based on a literature search (Supplemental Information), associations between PRS and structural MRI volumes of 10 anatomical ROIs were assessed. PRS associations with white matter integrity were also examined, because of prior publications of associations between diffusion tensor imaging (DTI) metrics and measures of risk taking or impulsivity^10,24^. Anatomical ROIs analysed were middle frontal gyrus, amygdala, orbitofrontal cortex, anterior cingulate, insular cortex, caudate, hippocampus, supra-marginal gyrus, nucleus accumbens and putamen. PRS were divided into quintiles^25^, and for each MRI outcome the top and bottom 20% were compared using linear regression/linear mixed model with hemisphere as a fixed factor (Supplemental Information). MRI outcomes were standardised so results reflect standard deviation change. After exclusion of participants who self-reported a neurological disorder at either the baseline assessment or imaging visit (*n* = 683, 6.9%) (Table S2), there were 9249 individuals with PRS and MRI measurements available for analysis.

### Expression quantitative trait locus analysis

The lead SNP from each locus was assessed for the possibility of genotype-specific gene expression patterns (or expression quantitative trait loci, eQTLs) in dorsolateral prefrontal cortex (DLPFC) using the Lieber Institute for Brain Development (LIBD) RNA-Seq data, accessed via the eQTL Browser^26^. For SNPs showing significant eQTLs in the LIBD dataset, we looked for replication in the CommonMind Consortium (CMC) DLPFC RNA-Seq data (*n* = 547)^27^, using the LIBD eQTL Browser. eQTLs that reached a threshold of *α* = 0.05 in the LIBD dataset (false discovery rate (FDR)-corrected) and replicated (defined as a threshold of *α* = 0.05 in the same direction) in the CMC dataset are reported. Tissue-specific expression patterns were assessed for implicated genes using the GTEx portal^28^ and the neurodevelopmental trajectories of implicated genes were assessed in the BrainCloud dataset^29^.

### Data mining

SNPs meeting the threshold for suggestive evidence of association (*p* < 1 × 10^−5^) were assessed for potential functional impact using the Variant Effect Predictor^30^. For each SNP, only the most severe consequence (https://www.ensembl.org/info/genome/variation/predicted_data.html) was considered. Lead SNPs and the locus (500 kb up and downstream of the lead SNPs) were queried using the GWAS catalogue. Psychiatric and metabolic phenotypes were reported.

## Results

Demographic characteristics are presented in Table S1. Risk takers were younger, more often men, more often current or ever-smokers, and more likely to report mood instability, a history of addiction or a history of mood disorders^13^. They were also more likely to have a university degree than controls^13^. As with our previous report^13^, test–retest reliability was 84.4% (inconsistent 13.2%, missing 2.4%, *n* = 14,551).

**Fig. 2:**
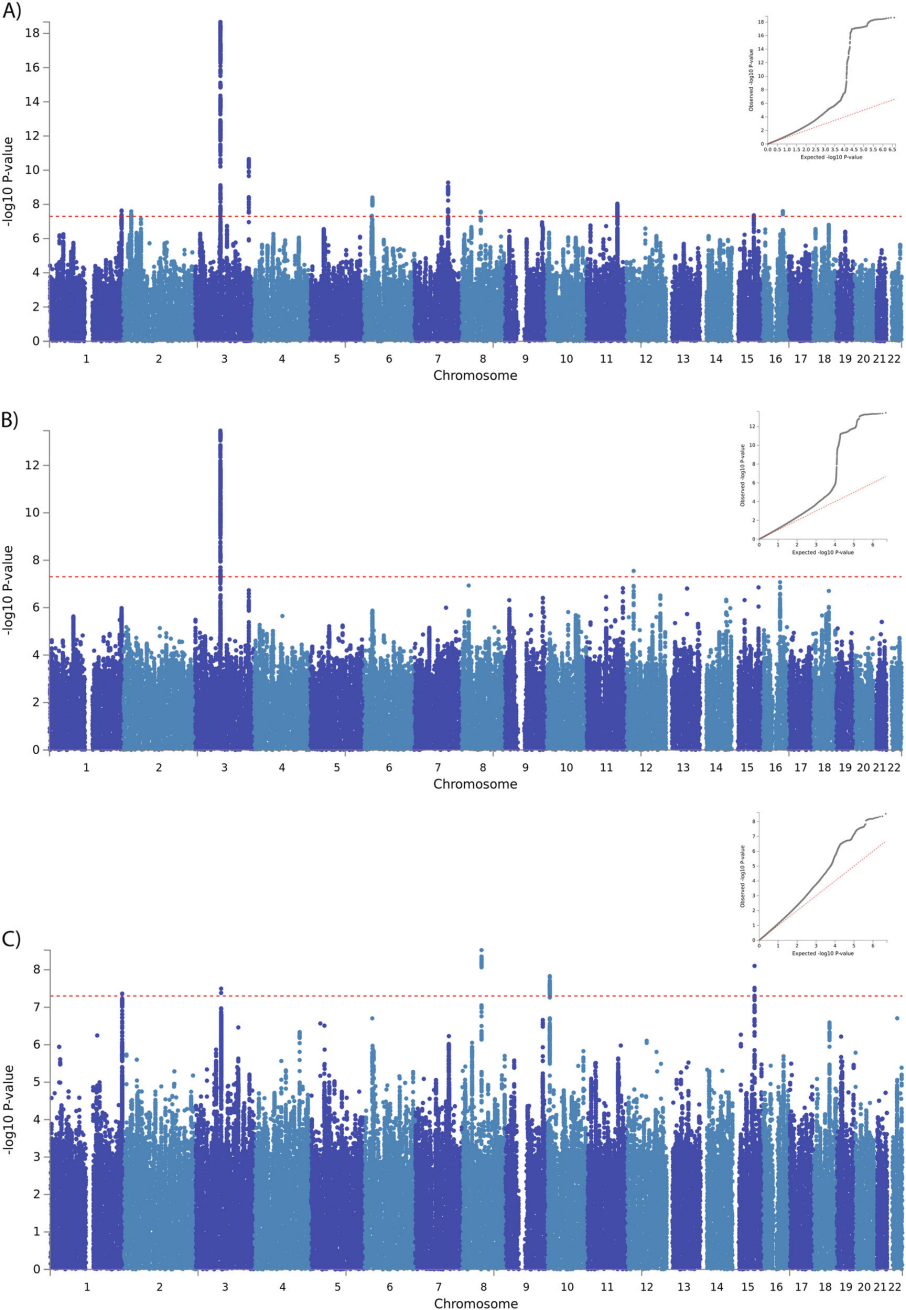
Genome-wide associations with risk-taking behaviour. Manhattan plots of association with risk-taking behaviour (inset QQ plot) for **a** all White British individuals, **b** White British men and **c** White British women

The risk-taking GWAS of white British participants identified 1162 genome-wide significant SNPs at 10 loci (Fig. 2a and Table S3), including the previously reported *CADM2* and *HLA* loci^13^.

### Association of two previously reported loci with self-reported risk taking

**Fig. 3:**
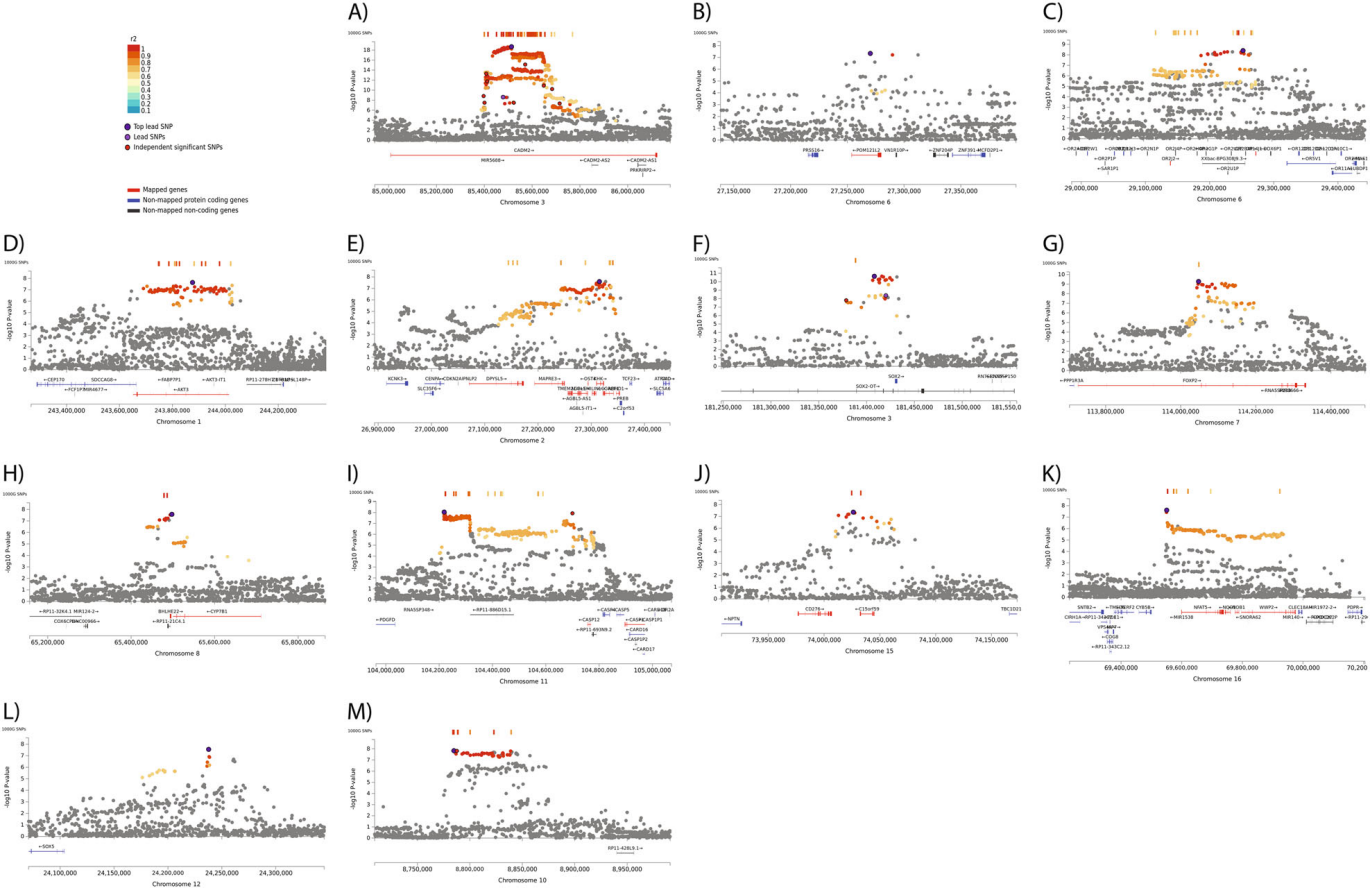
Regional plots of known loci: **a** *CADM2*, **b, c** extended *HLA* region; novel loci **d** *AKT3*, **e** *KHK*, **f** *SOX2*, **g** *FOXP2*, **h** *CYP17B1*, **i** *CASP12*, **j** *C15orf59*, **k** *NFAT5*; and sex-specific loci **l** *SOX5* and **m** Chr10 gene desert

The *CADM2* locus on Chr3 (85 Mb) contained 812 GWAS-significant SNPs (Fig. 3a and Table S3), including a novel lead SNP (rs542809491) and the previously reported lead SNP for risk taking, rs13084531^13^. Conditional analysis including the previous lead SNP (rs13084531) as a covariate had limited impact on the effect size of the new lead SNP (rs542809491) and did not remove the significance of the association (Table S4). Including rs542809491 as a covariate rendered the previous lead SNP nonsignificant. Indeed, after conditioning on rs542809491, no SNPs in this locus reached even suggestive association (*p* < 1 × 10^−5^), indicating that this locus contains only one signal.

On Chr6, significant SNPs were identified at 27 Mb and at 29 Mb (Figs. 3b, c and Table S3). As these fall within the extended *HLA* region, known to have extensive linkage disequilibrium (LD), conditional analysis was conducted on the two sets of SNPs together. The previous lead SNP in this region failed to meet the suggestive level of significance here (rs9379971, *p* = 4.94 × 10^−5^). Conditional analysis using this SNP increased the *p* of all SNPs in the region somewhat whereas conditioning on either the 27 Mb lead SNP (rs188973463) or the 29 Mb lead SNP (rs566858049) attenuated all associations (Table S5). Thus, this locus also appears to contain only one signal (index SNP rs188973463).

### Eight novel loci associated with self-reported risk taking were identified

Eight novel risk-taking-associated loci were identified (Table S3 and Figs. 3d–k). Conditional analyses demonstrated that only the Chr3 (181 Mb) locus was suggestive of a second signal (Table S6). To aid data mining, rs727644 (instead of 7:114156758 _GT_G) and rs10895735 (instead of 11:104700736 _ACTTCAC_A) were used as the lead SNPs of the loci on Chr7 and Chr11, respectively, based on minor allele frequency (MAF) similarity and nonsignificance in the conditional analyses.

### Two sex-specific loci were identified

Compared with the previous GWAS of risk-taking behaviour, the cohort size was more than doubled, allowing for well-powered sex-specific analyses. The characteristics of the sex-specific samples were comparable with the sex-combined samples (Table S7). In men, two GWAS-significant loci were identified (Fig. 2b, Table S3). The *CADM2* locus was the same as was identified in the sex-combined analysis, albeit 100 kb away, whereas the Chr12 locus (Fig. 3l) was unique to men and did not reach even suggestive significance (*p* < 1 × 10^−5^) in the sex-combined analysis. The women-only analysis identified five loci (Fig. 2c and Table S3). The lead SNPs at the *CADM2* and *CYP7B1* loci were the same as for the sex-combined analysis. The lead SNPs for the Chr1 (*AKT3)*, Chr15 (*C15orf59)* and Chr10 locus (specific to women) all reached suggestive evidence of association in the sex-combined analysis. The conditional analysis demonstrated that the women-only lead SNP of the *C15orf59* locus represented the same signal as the sex-combined analysis (conditional *p* = 0.2234). However, for the *AKT3* signal the results were inconclusive (conditional *p* = 3.5 × 10^−4^). The women-specific Chr10 locus lies within a gene desert (no coding genes within a flanking region of 500 kb up or downstream of the lead SNP) (Fig. 3m).

### Genetic overlap with psychiatric, behavioural and cognitive traits

LDSR demonstrated significant genetic overlap between self-reported risk taking and ADHD, SCZ, BD, MDD, PTSD, smoking, alcohol consumption and cannabis use, as well as with IQ (fluid intelligence) and BMI (Table 1). This is consistent with our previous report^13^.

**Table 1:** Genetic correlations of self-reported risk taking with psychiatric disorders and relevant other traits

Trait	rg	se	z	*p*	h2 obs	h2 obs se	h2 int	h2 int se	gcov int	gcov int se
ADHD	0.382	0.036	10.6047	**2.83E-26**	0.230	0.015	1.033	0.010	−0.005	0.007
PTSD	0.350	0.130	2.6952	**0.0070**	0.090	0.044	0.996	0.006	−0.001	0.005
BD	0.289	0.043	6.7072	**1.98E-11**	0.115	0.010	1.022	0.008	0.000	0.006
SCZ	0.250	0.026	9.5325	**1.54E-21**	0.249	0.010	1.036	0.011	−0.001	0.007
MDD	0.136	0.059	2.3215	**0.0203**	0.113	0.015	0.987	0.008	0.004	0.007
Anxiety (case–control)	−0.005	0.090	−0.0538	0.9571	0.074	0.029	1.003	0.007	0.002	0.005
Fluid intelligence	−0.154	0.032	−4.8381	**1.31E-06**	0.196	0.011	1.015	0.008	−0.004	0.005
Years of education	0.011	0.024	0.4646	0.6422	0.127	0.004	0.929	0.010	0.023	0.006
Lifetime cannabis use	0.428	0.069	6.2412	**4.34E-10**	0.091	0.016	0.999	0.007	−0.003	0.006
Ever smoker	0.292	0.050	5.8257	**5.69E-09**	0.074	0.007	0.999	0.006	−0.001	0.006
Alcohol (quantitative)	0.217	0.061	3.5338	**4.10E-04**	0.053	0.008	1.014	0.007	−0.005	0.006
BMI	0.076	0.025	3.0204	**0.0025**	0.139	0.007	0.643	0.010	−0.002	0.006
WHRadjBMI	0.054	0.031	1.7572	0.0789	0.093	0.007	0.854	0.010	−0.006	0.006
Caudate volume	−0.003	0.056	−0.0572	0.9544	0.247	0.038	0.969	0.006	0.004	0.005
Accumbens volume	−0.002	0.094	−0.0249	0.9801	0.084	0.037	0.981	0.006	0.004	0.005

*MDD* major depressive disorder, *BD* bipolar disorder, *SCZ* schizophrenia, *ADHD* attention-deficit hyperactivity disorder, *BMI* body mass index, *WHRadjBMI* waist:hip ratio adjusted for BMI, alcohol dependence DSM 5 criteria, *rg* regression coefficient, *se* standard error of the regression coefficient, *p* *p*-value for the regression analysis. **Bold** indicates nominally significant values (*P*<0.05)

### Consistency of associations in other ethnicities

The demographic characteristics of non-British White, South Asian, African-Caribbean and mixed ethnicities are presented in Table S8. Overall, when assessing consistency of effects across ethnicities, self-reported risk takers were more often men and more likely to be smokers and more often reported mood instability, history of addiction and mood disorders. The effect of the lead SNPs on risk taking in these ethnicities are presented in Table S9. In white non-British individuals, the *CADM2* (rs542809491), *FOXP2* (rs727644) and *CYP7B1* (rs189335278) loci demonstrated nominal significance with risk-taking behaviour. In South Asians, the *SOX2* (rs9841382) and *FOXP2* (rs727644) loci demonstrated nominal significance. No evidence of effects was observed in African-Caribbean or mixed ethnicities. Meta-analysis of all ethnicities demonstrated GWAS significance (*p* < 5 × 10^−8^) for 8 of 11 loci (Table 2). Of these loci, six had low heterogeneity (*I*^2^ was < 25%) and two had moderate heterogeneity (*I*^2^ was 25% < 50%), consistent with failure to detect effects in the separate ethnicities possibly being due to sample size rather than lack of true effects.

**Table 2:** Trans-ethnic meta-analysis of lead SNPs

CHR	BP	SNP	Locus	A1	A2	Freq1	FreqSE	Effect	StdErr	*P*	Direction	Isq^a^	ChiSq^a^	Df^a^	Het *P*
1	243,812,368	rs560977020	*AKT3*	t	c	0.66	0.07	0.034	0.005	**3.15E-10**	+++++	0	0.40	4	0.9825
2	27,315,252	rs2304681	*KHK*	a	g	0.37	0.01	−0.028	0.005	1.27E-07	--++-	51.3	8.21	4	0.08403
3	85,617,378	rs542809491	*CADM2*	a	t	0.39	0.04	0.053	0.005	**4.38E-23**	+++-+	27.1	5.48	4	0.2412
3	181,408,124	rs9841382	*SOX2*	t	c	0.84	0.08	−0.052	0.007	**7.24E-13**	-----	41.1	6.79	4	0.1476
6	27,766,842	rs188973463	*HLA*	t	g	0.74	0.04	0.037	0.006	**4.13E-09**	++--+	13.7	4.63	4	0.3269
	29,230,129	rs566858049		t	c	0.60	0.05	0.031	0.005	**7.24E-09**	++-++	6.1	4.26	4	0.3722
7	114,156,758	7:114156758	*FOXP2*	g	gt	0.64	0.01	0.041	0.005	**6.29E-14**	+++-+	2.2	4.09	4	0.3938
8	65,508,415	rs189335278	*CYP7B1*	a	t	0.11	0.01	−0.054	0.008	**7.71E-11**	----+	0	1.60	4	0.8088
11	104,700,736	11:104700736	*CASP12*	a	acttcac	0.26	0.05	0.034	0.006	**4.16E-08**	+0+++	24.9	5.33	4	0.2552
15	74,064,198	rs545973460	*C15orf59*	a	g	0.34	0.03	0.028	0.005	3.66E-07	+-+++	51.3	8.22	4	0.08382
16	69,550,486	rs145206681	*NFAT5*	t	c	0.06	0.01	0.056	0.011	3.23E-07	++-+-	29.2	5.65	4	0.227

Cohorts included White British, White non-British, South Asian, African-Carribean, mixed

*P* meta-analysis *p*-value, *Isq* measure of heterogeneity, *het P* heterogeneity *p*-value

^a^Analysis of heterogeneity. **Bold** indicates genome-wide significant values (*P*<5.00E-8)

### Risk-taking PRS and brain imaging phenotypes

Multiple strategies were employed to explore the impact of risk-taking SNPs on brain biology. One was to examine whether the genetic variants influenced the structure of brain regions previously implicated in risk-taking behaviours. Results of a secondary GWAS excluding the MRI subset were consistent with those for the discovery GWAS (Figure S2). Demographic characteristics of the MRI subset (Fig. 1) were generally comparable with the full cohort (Table S10), although there was enrichment for having a university degree and higher affluence.

Each SNP had only a small effect, therefore we also assessed the total genetic burden of all risk-taking loci using PRS. As it is likely that many variants (not only GWAS-significant SNPs) have effects on the anatomical ROIs, a variety of different *p*-value thresholds were employed^31^. Comparing the top versus bottom PRS quintile demonstrated that at some *p*-value thresholds, higher PRS was associated with lower volume of grey matter in the middle frontal gyrus and insular cortex (Table S11), but not with lower total grey matter volume or greater ventricular cerebrospinal fluid volume (both head size-normalised, Table S12). In addition, higher PRSs were associated with greater mean diffusivity (reflecting poorer white matter integrity, Table S13), but not with fractional anisotropy (FA). These findings were echoed by tract-specific analyses, where higher PRS was associated with greater MD in 9 out of 15 tracts (Table S14) but not with FA in any single tract (Table S15). Associations between the risk-taking PRS and MD but not FA are indicative of the greater sensitivity of MD. It is worth noting that PRS based on more relaxed *p*-value thresholds can demonstrate significance when the stringent ones do not, as increasing numbers of SNPs included in the more relaxed PRS contribute to increased genetic information, as well as increased statistical power. These structural MRI associations are tentative, therefore speculation as to their functional relevance is not warranted.

### Gene expression analysis

Eight of the lead SNPs (Table 2) were present in the LIBD dataset. Three of these showed robust eQTLs (rs2304681, rs3943093 and rs17187323). Most strikingly, the chromosome 2 lead SNP (rs2304681) is associated with the expression of several nearby genes (Table S16). The most numerous and statistically robust associations are with *CGREF1* (minimum FDR-corrected *p* = 3.4 × 10^−22^), with prominent associations also seen for *KHK* (*p* = 1.5 × 10^−13^) and *DPYSL5* (*p* = 4.2 × 10^−5^). Intriguingly, for both *CGREF1* and *KHK* there is evidence that the SNP may be associated with the expression of specific transcripts, because the rs2304681 A allele (associated with reduced risk taking) predicts increased expression of certain junctions/transcript features but decreased expression of others (Figure S4; Table S16). In contrast, for *DPYSL5*, the rs2304681 A allele uniformly predicts decreased expression (Table S16). *CGREF1, KHK* and *DPYSL5* are all expressed in brain (Figure S5). Notably, in the case of *CGREF1* and *DPYSL5*, the brain is the tissue in which these genes are most abundantly expressed.

Two of the SNPs found to predict risk taking only in women also showed eQTLs. The rs3943093 T allele (associated with lower risk of risk taking, Chr1) predicted increased expression of *SDCCAG8* (*p* = 7.7 × 10^−11^). The A allele of rs17187323 (associated with increased risk taking) predicted lower expression of *C15orf59* (*p* = 0.00014; Table S16).

All of the genes implicated in the eQTL analyses show some expression in human brain (Figure S5). Notably, in the case of *CGREF1*, *DPYSL5* and *C15orf59* expression in the brain is particularly prominent, compared with other tissues (Figure S5). Furthermore, all of the genes highlighted above show evidence of differential expression across development: *CGREF1*, *KHK* and *C15orf59* show greater expression in adult brain than foetal brain, whereas this pattern is reversed for *DPYSL5* and *SDCCAG8*.

### Data mining

The predicted functional consequences of risk-taking-associated SNPs (GWAS and suggestive significance) highlighted a number of missense variants with potentially moderate impact on genes (Table S17). One variant on Chr6 was predicted to have a high impact: rs539861690-A is predicted to give rise to a premature stop codon in *ZKSCAN4*. Conditional analysis of the Chr6 region demonstrates that adjusting for the Chr6 lead SNPs rs188973463 and rs566858049 reduced the association of rs539861690 with risk taking to nonsignificant (*p* = 0.2824) or nominal significance (*p* = 0.0129) respectively. This suggests that rs539861690 could be the functional variant in this region but as no genotype-specific expression patterns were identified for this SNP functional studies are required to verify this.

None of the lead SNPs have previously been reported to be associated with any trait in the GWAS catalogue (2018-01-31). These risk-taking loci have previously been associated with: educational attainment^32^, SCZ^33,34^ and PTSD^35^ (Chr1 locus); cognitive function^36,37^, educational attainment^32,38^, adiposity^39–41^ and alcohol consumption^42^ (*CADM2* locus); SCZ^43,44^ and ADHD^45^ (Chr6 locus); and sleep duration^46^ (*FOXP2* locus). Of the previously reported SNPs at these loci, 16 met the threshold for “suggestive” evidence of association with risk taking in this study (Table S18). Where the reported data allowed comparison, results were as expected (Table S18), with alleles, which increased risk of SCZ^33,34,43,44^ associated with increased risk taking; the allele for increased sleep duration^46^ associated with *reduced* risk taking; and the allele for increased information processing speed^37^ also associated with *reduced* risk taking. In contrast, the association between alleles for increased educational attainment^32^ and increased risk taking may seem counter-intuitive but is consistent with previous findings from UK Biobank (*n*~116,000)^13^. The allele associated with waist circumference^41^ was associated with increased risk taking, but the opposite was observed for BMI^39^, (although the BMI study was in a Japanese population^39^, whereas the risk-taking study was in a European study, so ethnic-specific effects (in regulation of BMI and/or risk taking) could be responsible for this discrepancy).

## Discussion

Examining the biology of risk-taking behaviour has the potential to improve our understanding of the pathophysiology of psychiatric disorders such as ADHD, SCZ and BD, as well as problem behaviours such as drug and alcohol misuse. We identified two known loci (*CADM2* and *HLA*), eight novel loci and two additional sex-specific loci associated with risk-taking behaviour. We observed that there was little heterogeneity in genetic effects across different ethnicities. We also identified significant genetic correlations between risk taking and several psychiatric disorders and eQTL analyses highlighted a number of potential candidate genes through which the SNPs might influence risk-taking behaviour.

The results presented here are consistent with previous (smaller) risk-taking GWAS^13^. The larger sample size reported here clarified that each of the known loci (*CADM2* and *HLA*) consisted of only one signal. Similarly, genetic correlations with ADHD, SCZ, BD, PTSD, smoking, use of cannabis, intelligence and BMI were comparable to those reported previously^13^. The nine signals in eight novel loci demonstrated similar effect sizes to those previously reported in UK Biobank and overlapped with loci previously associated with SCZ, sleep duration, alcohol consumption and processing speed (in the expected direction) (Table S18). As with other complex traits, the effects of the associated SNPs were modest (BETA = 0.033–0.067) compared with the effect of sex (BETA = 0.83) but comparable with the effect of age (BETA = 0.021). That increased risk taking positively associated with higher educational attainment was unexpected but is consistent with our previous report on risk taking^13^. It has been shown that (in adolescence), risk takers do not view their behaviour as risky^47^. Although we do not know if this is also the case for the UK Biobank participants, it would lead to underestimation (rather than inflation) of the true effect. In addition, we acknowledge that risk taking is based on a single question and it maybe unclear what exactly is captured by this phenotype (calculated versus impulsive risks for example), however, a more detailed study of the psychometric structure of risk taking supports the validity of our approach^48^. We cannot exclude that collider bias^49^ arising from patterns of self-selection into the UK Biobank cohort contributes to this (it is noted that self-reported risk takers in UK Biobank have an increased frequency of having a university degree than controls).

The finding of limited heterogeneity across ethnicities of these signals is preliminary, as the sample sizes are significantly smaller than that of the discovery analysis. However, this finding is plausible. Given that risk taking is likely to have serious evolutionary consequences, such traits are likely to be less varied than those with lower selection pressure.

Very recently, Clifton et al. published results from a GWAS of risk-taking behaviour in the UK Biobank^50^. They applied a different analysis strategy to the dataset and phenotype used here limited the report to a sex-combined genetic discovery experiment. By using all white British and white non-British participants and software enabling the inclusion of related individuals, their study had a larger sample size (*N* = 436,236) and identified of 26 significant loci (including most of the loci reported here). The Clifton et al. lead SNPs had *p* ≤ 0.0003 in our smaller and more homogeneous study (Table S19), suggesting that sample size is likely the reason for the identification of additional signals. Conditional analyses (including lead SNPs as covariates, Table S20) demonstrated that the same loci were identified at loci on Chr2 (*KHK/MAPRES2*), 3 (*SOX2 and CADM2*), 7 (*FOXP2*), 8 (*CYP7B1*), 11 (*CASP12/PDGFD*). These analyses also demonstrated that the loci reported on Chr6 (at 27 and 29 Mb) likely contain the same signal. Trans-ethnic analysis of the Clifton et al. lead SNPs was consistent with our analysis, with generally low heterogeneity for risk-taking loci (Table S21). PRS analyses using the Clifton et al. summary statistics demonstrated consistent effects sizes, but associations were attenuated (Tables S22-S26).

EQTL studies highlighted some genes of interest for further biological investigation. At the Chr2 locus, *CGREF1*, *KHK* and *DPYSL5* are particularly implicated. *CGREF1* encodes a secretory protein involved in cell adhesion and proliferation^51,52^. Despite its abundant expression in the brain (Figure S5), its function remains unexplored. *KHK* encodes ketohexokinase, an enzyme involved in fructose metabolism. Although some studies demonstrate fructose metabolism in the brain^53,54^, another intriguing possibility is that KHK’s role in brain is as a protein kinase^55^. *DPYSL5*, which encodes collapsin response mediator protein (CRMP) 5, is involved in neurogenesis, dendritic development and synaptic plasticity^56,57^. *DPYSL5* null mice are viable and grossly normal^57^ and it will be of significant interest to investigate cognitive functions in these mice, given our findings and impairments in learning and memory demonstrated for other members of the CMRP family^58^. The Chr1 (women only) lead SNP predicted expression of *SDCCAG8*, which is involved in cortical development^59^ and mutations are characterised by cognitive impairments^60^. At the Chr15, we highlight *C15orf59*, which encodes a postsynaptic density protein that regulates inhibitory neurotransmission and hippocampal excitability^61^. Taken together, our eQTL analyses identified a number of candidate genes involved in synaptic plasticity and neurogenesis. They also emphasise the need to better understand the roles played by *CGREF1* and *KHK* in the brain. Finally, it is notable that all the genes implicated by our eQTL analyses show differential expression in the foetal versus adult brain, emphasising the importance of considering their potential impact on brain development, as well as their role in the adult brain.

The lead Chr2 SNP shows evidence of selective and/or differential associations with specific splice isoforms of *CGREF1* and *KHK*. This is notable given the evidence suggesting that RNA splicing is a key mechanism mediating effects of disease-associated, non-coding variants, including those linked with SCZ^62^. For *CGREF1*, although multiple transcripts are annotated, the functional impact of splicing is unknown. Functionally distinct splice isoforms of KHK have been reported^63,64^; however, the junction/exon implicated by our eQTL analysis affects a different region of the KHK gene (Table S16). Indeed, the junction showing robust association with rs2304681 (which skips exons 2 and 3) is not present in any of the currently annotated *KHK* transcripts. These findings emphasise the importance of understanding the complete transcript structure of genes relevant in the translation of genomic findings into biological insights. In the case of many (if not most) genes, the complement of splice isoforms present in human brain has been little explored^65^. Our findings add further weight to the increasing body of evidence^66^ suggesting that understanding isoform diversity, and its regulation by *cis*-acting factors, will be critical to unpick the biological impact of SNPs arising from GWAS.

This study is comparable with those of most complex traits investigated to date, both in terms of the risk variants having only a small effect on risk-taking behaviour and the challenges for translating the findings into biological mechanisms. Despite this, our findings contribute substantial new knowledge on the biology of risk taking and highlight several candidate genes for further investigation. This work will stimulate future experimental studies to elucidate our understanding of an important but complex trait, which contributes to a very broad range of adverse mental and physical health outcomes.

## Electronic supplementary material

Supplemental information

Supplementary Figure 1

Supplementary Figure 2

Supplementary Figure 3

Supplementary Figure 4

Supplementary Figure 5

Supplementary Figure 6

Supplementary Table 1

Supplementary Table 2

Supplementary Table 3

Supplementary Table 4

Supplementary Table 5

Supplementary Table 6

Supplementary Table 7

Supplementary Table 8

Supplementary Table 9

Supplementary Table 10

Supplementary Table 11

Supplementary Table 12

Supplementary Table 13

Supplementary Table 14

Supplementary Table 15

Supplementary Table 16

Supplementary Table 17

Supplementary Table 18

Supplementary Table 19

Supplementary Table 20

Supplemental Table 21

Supplementary Table 22

Supplementary Table 23

Supplementary Table 24

Supplementary Table 25

Supplementary Table 26
