# Supplementary material for: Genetics of self-reported risk-taking behaviour, trans-ethnic consistency and relevance to brain gene expression
Source: Transl Psychiatry. 2018 Sep 4;8:178. doi: 10.1038/s41398-018-0236-1 (PMC6123450; doi:10.1038/s41398-018-0236-1)
Supplement: Supplementary file 1 — Supplemental information [file 41398_2018_236_MOESM1_ESM.docx]

**Genetics of risk-taking behaviour and relevance to brain gene expression and white matter integrity**

**Supplementary Methods**

***Genotyping, imputation and quality control***

UK Biobank samples were genotyped with the Affymetrix UK BiLEVE Axiom array (Santa Clara, CA, USA (10.2%)) or the Affymetrix UK Biobank Axiom array (89.8%), which have at least 95% of content in common. Only autosomal data were available. Prior to imputation, SNPs were excluded for minor allele frequency (MAF) <1% of if the were multi-allelic. Imputation using the Haplotype Reference Consortium reference panel was conducted for all participants (n=502 664) with phasing being conducted with a modified version of SHAPEIT2 and imputation performed using IMPUTE2 (implemented on a C++ platform) (1-3). Quality control was conducted centrally at the Wellcome Trust Centre for Human Genetics before data release (4). Recommended UK Biobank genomic analysis exclusions were applied (Biobank Data Dictionary item #22010), with participants being excluded due to quality control failure, relatedness, sex mismatch (reported compared to genetic) and SNPs being were removed due to deviation from Hardy–Weinberg equilibrium (*P*<1x10^−6^), low MAF (<0.01), poor imputation quality (<0.4) and missingness (>10%).

***Selection of discovery sample set***

It is well established that there are significant differences in genetic architecture and allele frequencies across Europe (5). This is also true in the UK Biobank data, with those self-reporting white British ancestry clustering together, whereas self-reported white non-British participants demonstrate (Figure S1) more diverse genetic backgrounds. In order to maximise homogeneity, the discovery cohort included only those with self-reported white British ancestry (Figure 1).

***Genetic correlations with related traits***

Published summary statistics from GWAS of ADHD, schizophrenia, BD, MDD, anxiety, PTSD, fluid intelligence, years of education, smoking status (ever smoked) and lifetime cannabis use which were available courtesy of the Psychiatric Genetics Consortium (https://www.med.unc.edu/pgc). Obesity is frequently concomitant with psychiatric disorders and overeating can be considered a problem behaviour. Furthermore, reward circuitry is implicated in regulation of obesity (6), thus BMI (reflecting total obesity) was also included in this analysis (courtesy of the GIANT consortium (http://portals.broadinstitute.org/collaboration/giant/index.php/Main_Page)).

***Polygenic risk score calculation***

PRS were calculated using p-value thresholds of p<5x10^-8^, p<1x10^-5^, p<0.001 and p<0.05. LD pruning was performed in PLINK, on a random sample of 10,000 individuals using an r^2^>0.05 in a 250kb window. The SNP with the lowest p-value was selected from each of the LD-clumped SNP sets. Where 2 or more SNPs from a set had the same p-value, the SNP with the larger beta coefficient was used. The scores were calculated in PLINK to produce a per-allele weighted score (without mean imputation).

***Selection of brain regions of interest (ROIs).***

A (non-exhaustive) literature search was conducted for studies of the associations between measures of ‘risk taking’, ‘risky decision making’ or ‘impulsivity’ and structural imaging measures of grey matter volumes or cortical thickness (Supplemental Information). Based on the 47 relevant studies revealed, we selected the 8 cortical/subcortical ROIs which were most commonly associated with risk taking/impulsivity measures, reported in between 2 and 6 studies: middle frontal gyrus, amygdala, orbitofrontal cortex, anterior cingulate, insular cortex, caudate, hippocampus, supramarginal gyrus (7-24). We also included the nucleus accumbens and putamen as ROIs, as these brain regions implicated in reward circuits have been consistently linked to risky decision making in meta-analyses of functional MRI studies (25, 26).

***MRI acquisition and pre-processing***

The details of the UK Biobank imaging protocol and data processing has previously been described (27) and are available online (<http://www.fmrib.ox.ac.uk/ukbiobank/index.html>). In brief, brain MRI scanning was conducted at a single site (Manchester) using a Siemens Skyra 3T scanner with a 32-channel head coil. T1-weighted structural images were acquired using a 3D MPRAGE sequence (resolution 1mm^3^). Tissue volumes and volumes of 139 brain regions were derived using FAST (FMRIB’s Automated Segmentation Tool) (28). Total volume of white matter hyper-intensities was derived using the BIANCA tool from T2-weighted (Fluid-Attenuated Inversion Recovery) images alongside T1 images.

Diffusion MRI was conducted using a Stejskal-Tanner pulse sequence (fov = 104x104x72; TE = 92 ms). Gradient distortion correction was applied, and data were corrected for eddy currents and head motion using FSL’s Eddy tool (<http://fsl.fmrib.ox.ac.uk/fsl/fslwiki/EDDY>). Measures of common diffusion tensor imaging indices of white matter integrity, namely fractional anisotropy (FA), and mean diffusivity (MD), were acquired for 27 white matter tracts after modelling of within-voxel tract structure using BEDPOSTx and PROBTRACKx. For both structural T1 and DTI measures, FSL (FMRIB Software Library) was used (29). For further details on MRI variables see <https://biobank.ctsu.ox.ac.uk/crystal/docs/brain_mri.pdf>. Volumes (mm^3^) of the 139 regions are currently available as UK Biobank Imaging Derived Phenotypes (IDPs) for around 10,000 UK Biobank participants.

White matter tract integrity was assessed using general components of FA and MD (*g*FA; *g*MD), derived using principal component analysis across the 27 white matter tract IDPs (30, 31). Scores were extracted for the first unrotated component: this first factor accounted for 44.15% of the total variance in FA, and 49.6% in MD.

***Analysis of polygenic risk score effects on MRI***

For bilateral tracts/anatomical ROIs (all ROIs and all tracts apart from forceps major, forceps minor, middle cerebellar peduncle), linear mixed models first examined PRS*hemisphere interactions. None of these interactions reached significance for any tract or region, and so results reported are those with hemisphere as a fixed factor (without the hemisphere*PRS interaction). Associations between PRS and tract-specific measures of FA and MD for each of 15 tracts (12 of which were bilateral) were also examined (see Tables 4 and 5 for tracts). Models were adjusted for age at MRI visit, age^2^, sex, genotype array and the first eight genetic principal components. Anatomical ROI and white matter hyper-intensity analyses were additionally adjusted for total brain volume (calculated by summing total grey matter, white matter and ventricular cerebrospinal fluid (CSF) volume). Tissue type measures (total grey matter, white matter and ventricular CSF) were normalised for head size. False discovery rate (FDR) correction was applied (32).

These individuals were predominantly white British individuals (Figure 1) and overlapped with the discovery GWAS. To avoid problems with prediction into the same sample, the discovery GWAS was re-run excluding the MRI subset (removal of 2% of controls and 2.3% of risk-takers) and the summary statistics from this run were used for calculating PRS, as previously described (33)

**Supplementary References**

1. Biobank U (2015): Genotype imputation and genetic association studies of UK Biobank, Interim Data Release. 11 September 2015.

2. Delaneau O, Zagury JF, Marchini J (2013): Improved whole-chromosome phasing for disease and population genetic studies. *Nat Methods*. 10:5-6.

3. Howie B, Marchini J, Stephens M (2011): Genotype imputation with thousands of genomes. *G3 (Bethesda)*. 1:457-470.

4. Biobank U (2015): Genotyping of 500,000 UK Biobank participants. Description of sample processing workflow and preparation of DNA for genotyping. 11 September 2015.

5. Huckins LM, Boraska V, Franklin CS, Floyd JA, Southam L, Gcan, et al. (2014): Using ancestry-informative markers to identify fine structure across 15 populations of European origin. *Eur J Hum Genet*. 22:1190-1200.

6. Locke AE, Kahali B, Berndt SI, Justice AE, Pers TH, Day FR, et al. (2015): Genetic studies of body mass index yield new insights for obesity biology. *Nature*. 518:197-206.

7. Asensio S, Morales JL, Senabre I, Romero MJ, Beltran MA, Flores-Bellver M, et al. (2016): Magnetic resonance imaging structural alterations in brain of alcohol abusers and its association with impulsivity. *Addict Biol*. 21:962-971.

8. Charpentier J, Dzemidzic M, West J, Oberlin BG, Eiler WJ, 2nd, Saykin AJ, et al. (2016): Externalizing personality traits, empathy, and gray matter volume in healthy young drinkers. *Psychiatry Res Neuroimaging*. 248:64-72.

9. Coutinho J, Ramos AF, Maia L, Castro L, Conceicao E, Geliebter A, et al. (2015): Volumetric alterations in the nucleus accumbens and caudate nucleus in bulimia nervosa: a structural magnetic resonance imaging study. *Int J Eat Disord*. 48:206-214.

10. Dang LC, Samanez-Larkin GR, Young JS, Cowan RL, Kessler RM, Zald DH (2016): Caudate asymmetry is related to attentional impulsivity and an objective measure of ADHD-like attentional problems in healthy adults. *Brain Struct Funct*. 221:277-286.

11. Depue BE, Olson-Madden JH, Smolker HR, Rajamani M, Brenner LA, Banich MT (2014): Reduced amygdala volume is associated with deficits in inhibitory control: a voxel- and surface-based morphometric analysis of comorbid PTSD/mild TBI. *Biomed Res Int*. 2014:691505.

12. Drobetz R, Hanggi J, Maercker A, Kaufmann K, Jancke L, Forstmeier S (2014): Structural brain correlates of delay of gratification in the elderly. *Behav Neurosci*. 128:134-145.

13. Fradkin Y, Khadka S, Bessette KL, Stevens MC (2017): The relationship of impulsivity and cortical thickness in depressed and non-depressed adolescents. *Brain Imaging Behav*. 11:1515-1525.

14. Hill SY, Wang S, Kostelnik B, Carter H, Holmes B, McDermott M, et al. (2009): Disruption of orbitofrontal cortex laterality in offspring from multiplex alcohol dependence families. *Biol Psychiatry*. 65:129-136.

15. Lin CS, Lin HH, Wu SY (2016): Functional and Structural Signatures of the Anterior Insula are associated with Risk-taking Tendency of Analgesic Decision-making. *Sci Rep*. 6:37816.

16. Mei S, Xu J, Carroll KM, Potenza MN (2015): Self-reported impulsivity is negatively correlated with amygdalar volumes in cocaine dependence. *Psychiatry Res*. 233:212-217.

17. Meyer-Lindenberg A, Buckholtz JW, Kolachana B, A RH, Pezawas L, Blasi G, et al. (2006): Neural mechanisms of genetic risk for impulsivity and violence in humans. *Proc Natl Acad Sci U S A*. 103:6269-6274.

18. Moreno-Lopez L, Catena A, Fernandez-Serrano MJ, Delgado-Rico E, Stamatakis EA, Perez-Garcia M, et al. (2012): Trait impulsivity and prefrontal gray matter reductions in cocaine dependent individuals. *Drug Alcohol Depend*. 125:208-214.

19. O'Neill A, D'Souza A, Carballedo A, Joseph S, Kerskens C, Frodl T (2013): Magnetic resonance imaging in patients with borderline personality disorder: a study of volumetric abnormalities. *Psychiatry Res*. 213:1-10.

20. Rahman AS, Xu J, Potenza MN (2014): Hippocampal and amygdalar volumetric differences in pathological gambling: a preliminary study of the associations with the behavioral inhibition system. *Neuropsychopharmacology*. 39:738-745.

21. Soloff P, White R, Diwadkar VA (2014): Impulsivity, aggression and brain structure in high and low lethality suicide attempters with borderline personality disorder. *Psychiatry Res*. 222:131-139.

22. Takeuchi H, Tsurumi K, Murao T, Takemura A, Kawada R, Urayama SI, et al. (2017): Common and differential brain abnormalities in gambling disorder subtypes based on risk attitude. *Addict Behav*. 69:48-54.

23. Tschernegg M, Pletzer B, Schwartenbeck P, Ludersdorfer P, Hoffmann U, Kronbichler M (2015): Impulsivity relates to striatal gray matter volumes in humans: evidence from a delay discounting paradigm. *Front Hum Neurosci*. 9:384.

24. Vollm BA, Zhao L, Richardson P, Clark L, Deakin JF, Williams S, et al. (2009): A voxel-based morphometric MRI study in men with borderline personality disorder: preliminary findings. *Crim Behav Ment Health*. 19:64-72.

25. Liu X, Hairston J, Schrier M, Fan J (2011): Common and distinct networks underlying reward valence and processing stages: a meta-analysis of functional neuroimaging studies. *Neurosci Biobehav Rev*. 35:1219-1236.

26. Silverman MH, Jedd K, Luciana M (2015): Neural networks involved in adolescent reward processing: An activation likelihood estimation meta-analysis of functional neuroimaging studies. *Neuroimage*. 122:427-439.

27. Miller KL, Alfaro-Almagro F, Bangerter NK, Thomas DL, Yacoub E, Xu J, et al. (2016): Multimodal population brain imaging in the UK Biobank prospective epidemiological study. *Nat Neurosci*. 19:1523-1536.

28. Zhang Y, Brady M, Smith S (2001): Segmentation of brain MR images through a hidden Markov random field model and the expectation-maximization algorithm. *IEEE Trans Med Imaging*. 20:45-57.

29. Jenkinson M, Beckmann CF, Behrens TE, Woolrich MW, Smith SM (2012): Fsl. *Neuroimage*. 62:782-790.

30. Cox SR, Ritchie SJ, Tucker-Drob EM, Liewald DC, Hagenaars SP, Davies G, et al. (2016): Ageing and brain white matter structure in 3,513 UK Biobank participants. *Nat Commun*. 7:13629.

31. Reus LM, Shen X, Gibson J, Wigmore E, Ligthart L, Adams MJ, et al. (2017): Association of polygenic risk for major psychiatric illness with subcortical volumes and white matter integrity in UK Biobank. *Sci Rep*. 7:42140.

32. Benjamini Y, Hochberg Y, Benjamini Y, Hochberg Y (1995): Controlling the false discovery rate: a practical and powerful approach to multiple testing. *J R Stat Soc B*. 57:289-300.

33. Strawbridge RJ, Ward J, Cullen B, Tunbridge EM, Hartz S, Bierut L, et al. (2018): Genome-wide analysis of self-reported risk-taking behaviour and cross-disorder genetic correlations in the UK Biobank cohort. *Transl Psychiatry*. 8:39.

**Supplementary Tables**

Supplemental Table 1. Description of UK Biobank participants included in the risk-taking GWAS (discovery analysis).

Supplementary Table 2: Exclusion criteria for MRI analysis

Supplemental Table 3: Lead SNPs in risk-taking associated loci

Supplementary Table 4: Conditional analysis of the CADM2 locus

Supplementary Table 5: Conditional analysis of the HLA region

Supplementary Table 6: Conditional analysis of the SOX2 locus

Supplementary Table 7: Description of UK Biobank participants included in the sex-specific analyses of risk-taking

Supplementary Table 8: Demographics of individuals included in the analysis of other ethnicities.

Supplementary Table 9: Effect of lead SNPs on risk-taking in additional ethnicities

Supplementary Table 10: Demographics of the MRI subset

Supplemental Table 11: Associations between PRS for risk-taking (top quintile vs. bottom quintile) and standardised grey matter volumes of cortical/subcortical regions of interest.

Supplemental Table 12. Associations between PRS for risk-taking (top quintile vs. bottom quintile) and white matter integrity general components.

Supplemental Table 13: Associations between PRS for risk taking (top quintile vs. bottom quintile) and standardised total tissue volumes and white matter hyperintensity volumes.

Supplementary Table 14. Associations between PRS for risk taking (top quintile vs. bottom quintile) and tract-specific Mean Diffusivity

Supplementary Table 15. Associations between PRS for risk taking (top quintile vs. bottom quintile) and tract-specific Fractional Anisotropy

Supplementary Table 16: Lead SNPs with replicated eQTLs in the dorsolateral prefrontal

cortex

Supplementary Table 17: Predicted effects of SNPs with suggestive evidence of association with risk-taking

Supplementary Table 18: Effects of SNPs within 500kb of the lead risk-taking SNPs on relevant psychiatric and cardiometabolic traits.

Supplementary Table 19: Results of loci reported by Clifton et al in this study

Supplementary Table 20: Conditional analyses of lead risk-taking SNPS

Supplementary Table 21: Trans-ethnic meta-analysis of Clifton et al lead SNPs

Supplementary Table 22. Associations between PRS for risk taking (top quintile vs. bottom quintile) and standardised volumes of cortical/subcortical regions of interest.

Supplementary Table 23. Associations between PRS for risk taking (top quintile vs. bottom quintile) and white matter integrity general components.

Supplementary Table 24. Associations between PRS for risk taking (top quintile vs. bottom quintile) and standardised total tissue volumes and white matter hyperintensity volumes.

Supplementary Table 25. Associations between PRS for risk taking (top quintile vs. bottom quintile) and tract-specific Fractional Anisotropy

Supplementary Table 26. Associations between PRS for risk taking (top quintile vs. bottom quintile) and tract-specific Mean Diffusivity

**Supplementary Figures**

Supplementary Figure 1: Scatter plot of the first two principle components for participants of self-reported white British (red dots) and white non-British (blue dots) ancestry.

Supplementary Figure 2: Manhattan plot of risk-taking, excluding the MRI-subset individuals. Inset: qq plot.

Supplementary Figure 3: Tissue expression profile for genes implicated in Chr6. (A) *AL022393.7,* (B) Z*SCAN31,* (C) *ZSCAN23*, (D) *RP5-874C20.3* and (E) *C6orf100*.

Supplemental Figure 4: Genotype-specific expression patterns of Chr2 rs2304681, where the A allele predicts increased expression of some features (left) but decreased expression of others (right) for both the *CGREF1* (a) and *KHK* (b) genes.

Supplemental Figure 5: Tissue expression patterns of candidate genes, A) *CGREF1*, B) *KHK*, C) *DPYSL5*, D) *SDCCAG8*, E) *C15orf59*.

Supplemental Figure 6: Expression patterns of candidate genes over the lifespan, A) *CGREF1*, B) *KHK*, C) *DPYSL5*, D) *SDCCAG8*, E) *C15orf59*.
