# Supplementary figures and images for: Genetics of self-reported risk-taking behaviour, trans-ethnic consistency and relevance to brain gene expression
Source: Transl Psychiatry. 2018 Sep 4;8:178. doi: 10.1038/s41398-018-0236-1 (PMC6123450; doi:10.1038/s41398-018-0236-1)

Supplementary Figure 1

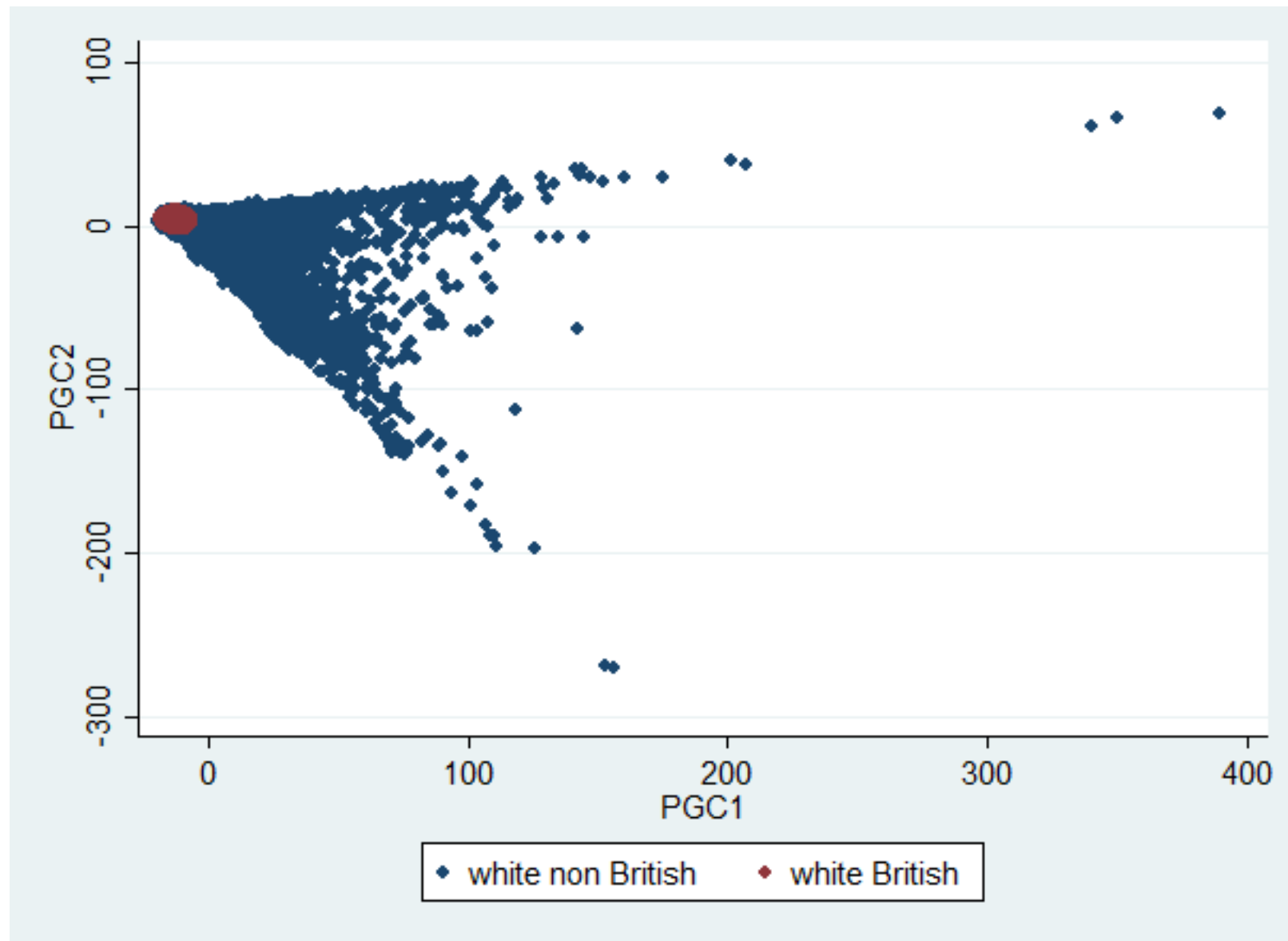

Supplement: Supplementary file 2 — Supplementary Figure 1 [file 41398_2018_236_MOESM2_ESM.pdf]

Supplementary Figure 2

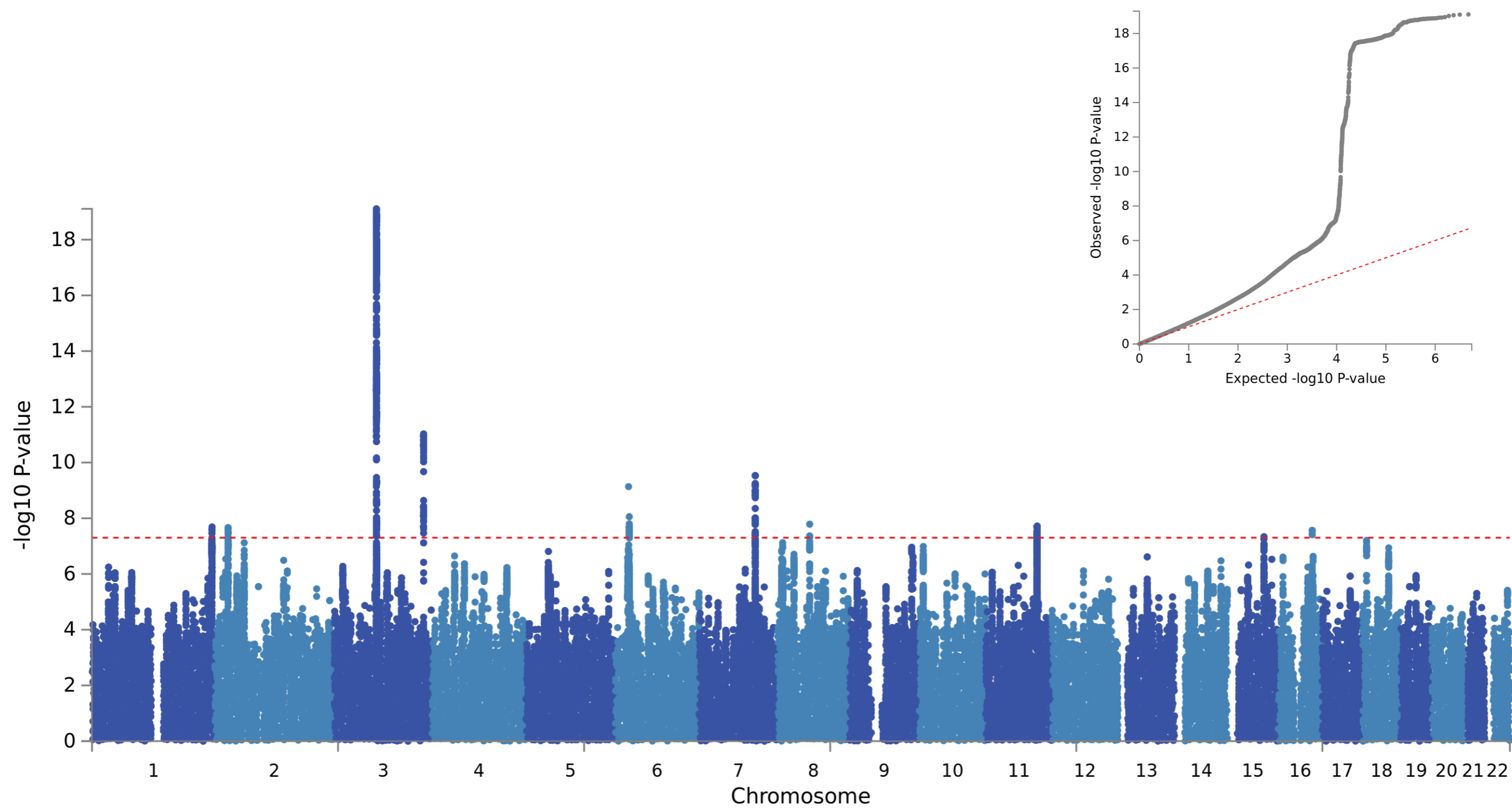

Supplement: Supplementary file 3 — Supplementary Figure 2 [file 41398_2018_236_MOESM3_ESM.pdf]

A)

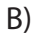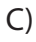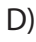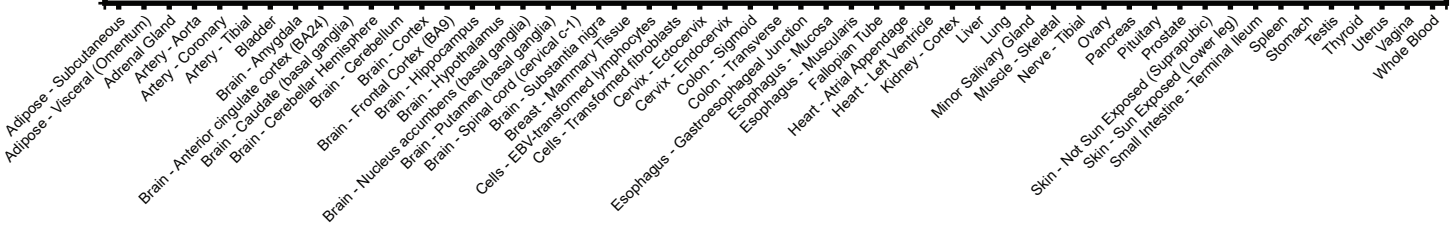

E)

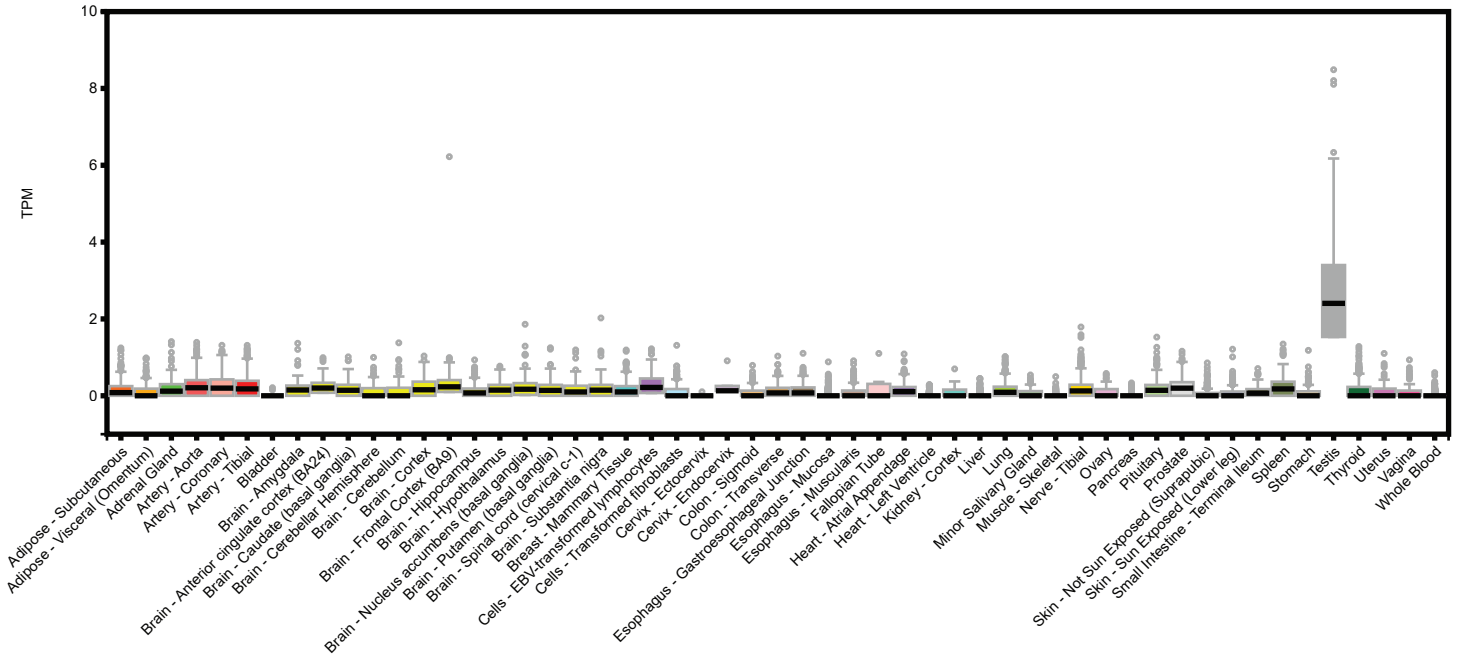

Supplement: Supplementary file 4 — Supplementary Figure 3 [file 41398_2018_236_MOESM4_ESM.pdf]

Supplementary Figure 4

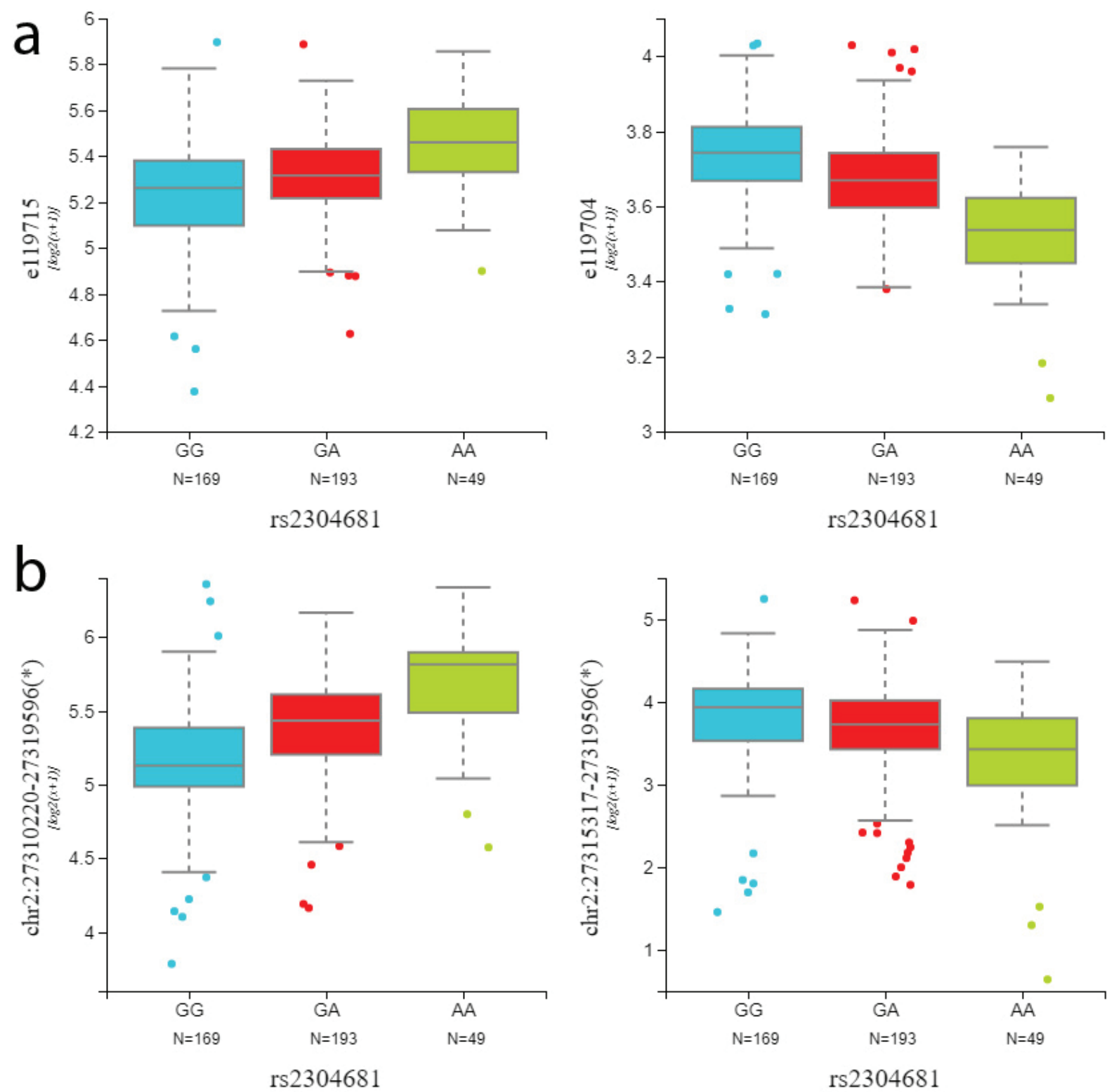

Supplement: Supplementary file 5 — Supplementary Figure 4 [file 41398_2018_236_MOESM5_ESM.pdf]

Supplementary Figure 5

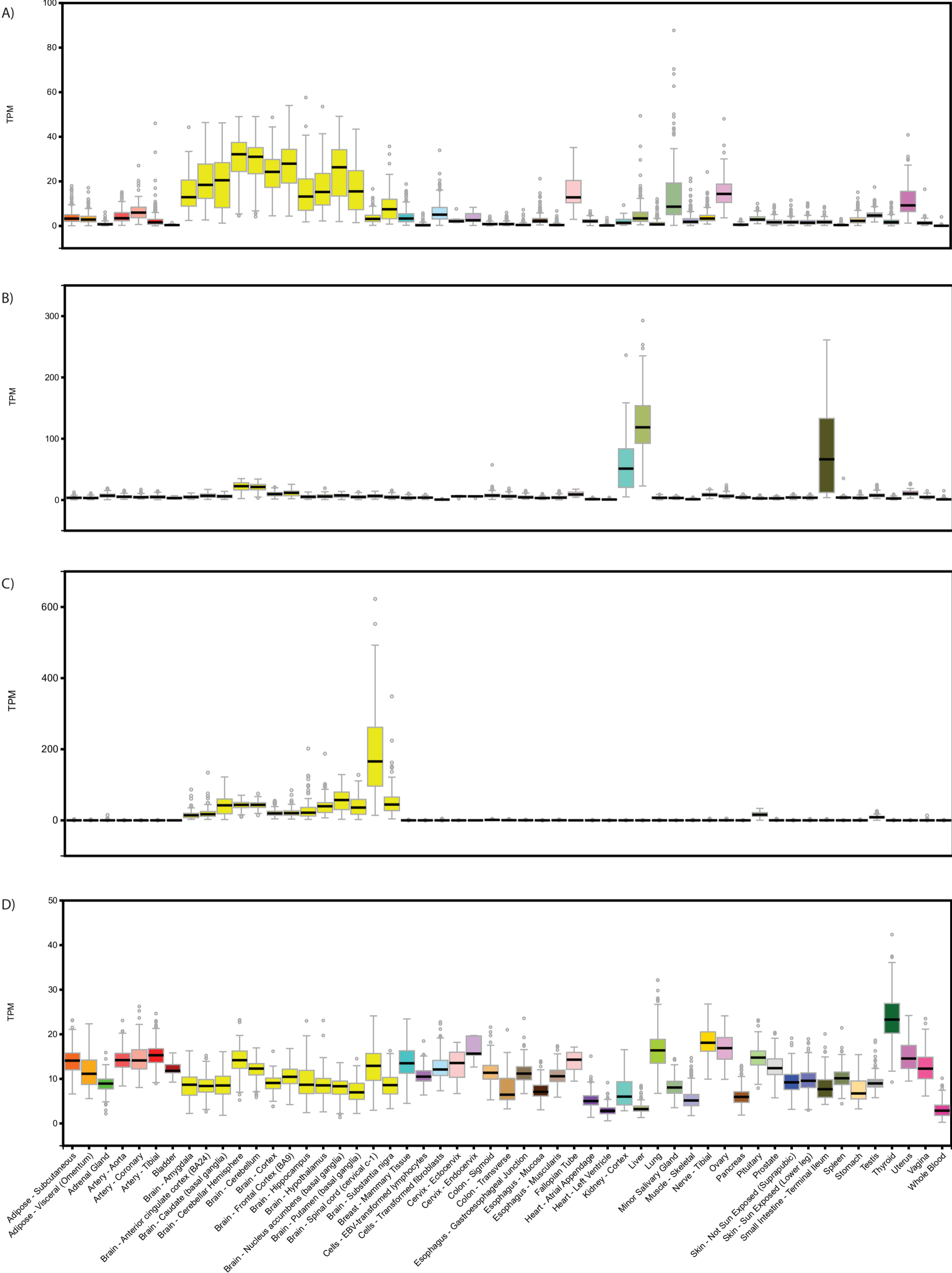

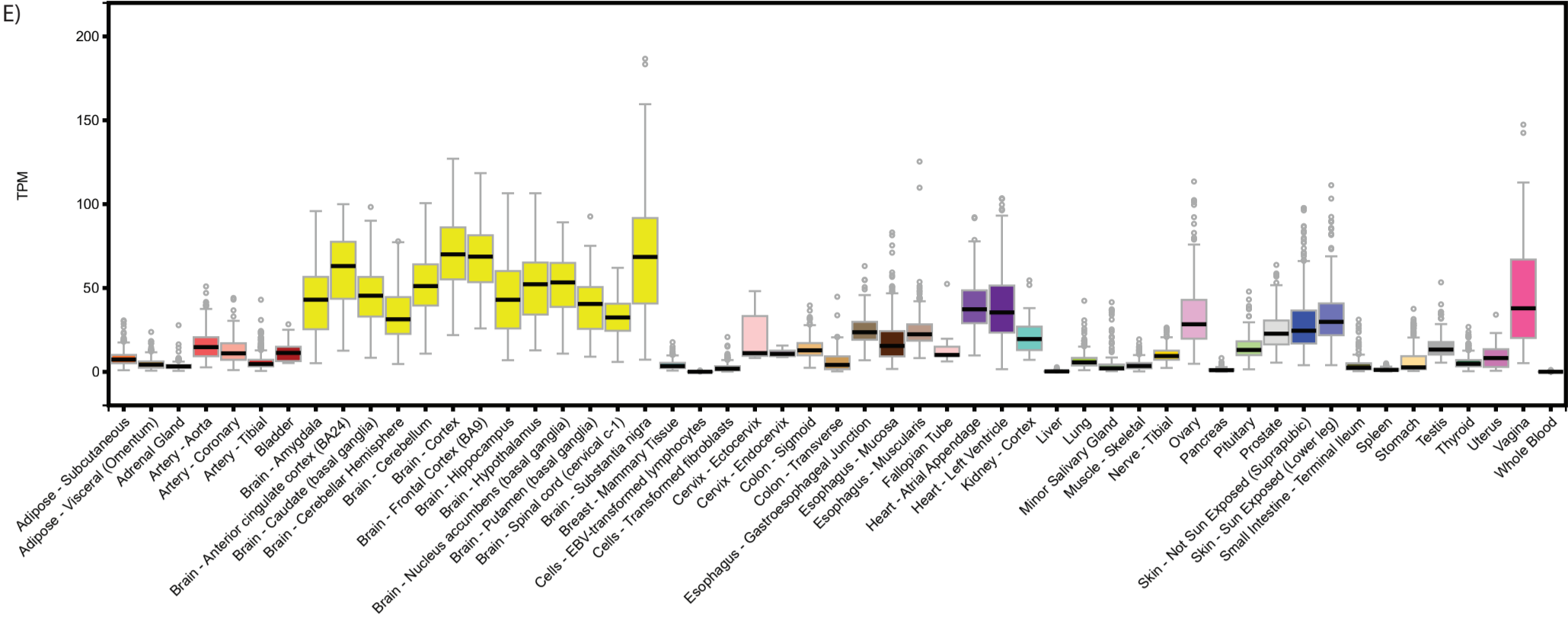

Supplement: Supplementary file 6 — Supplementary Figure 5 [file 41398_2018_236_MOESM6_ESM.pdf]

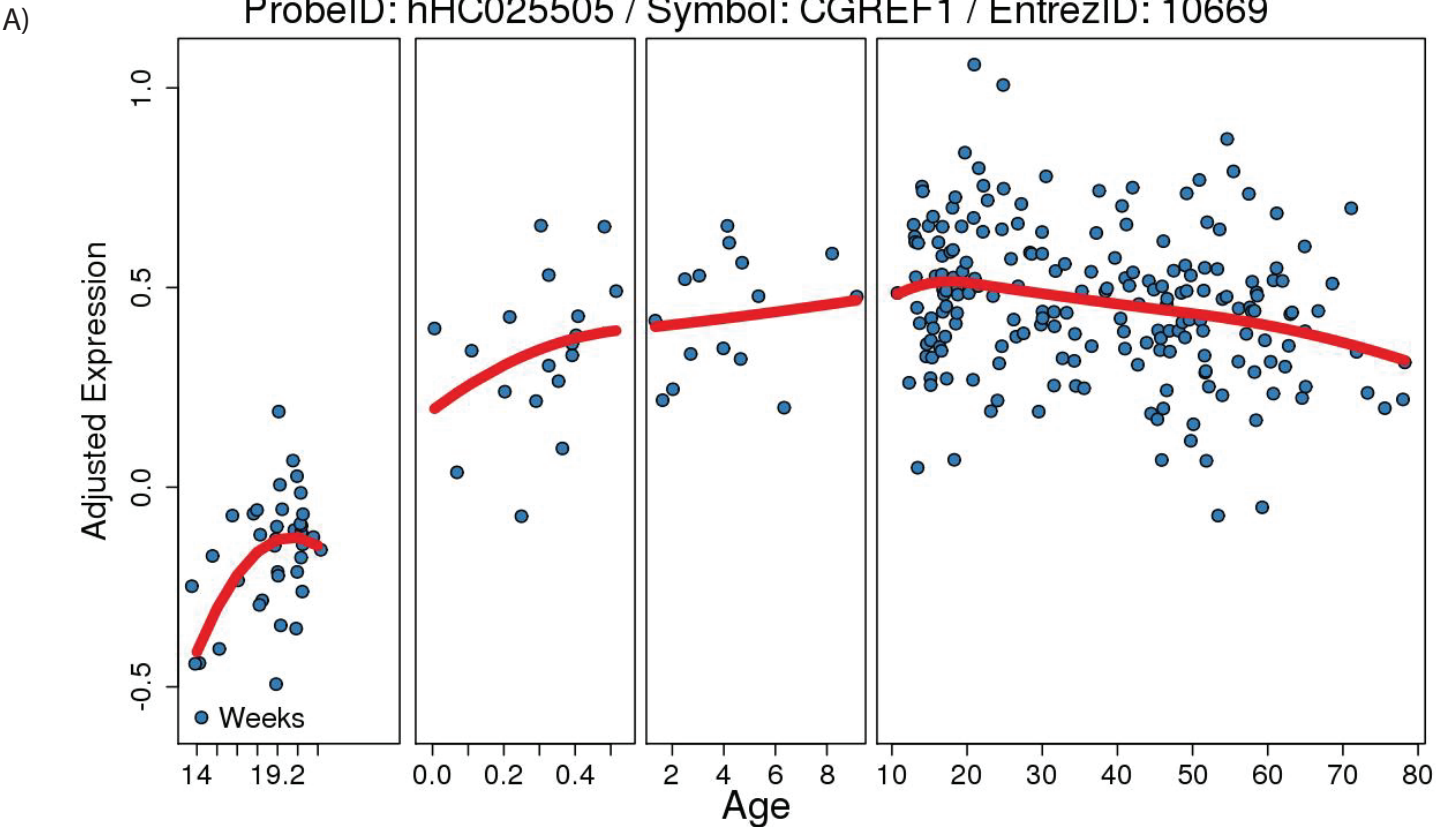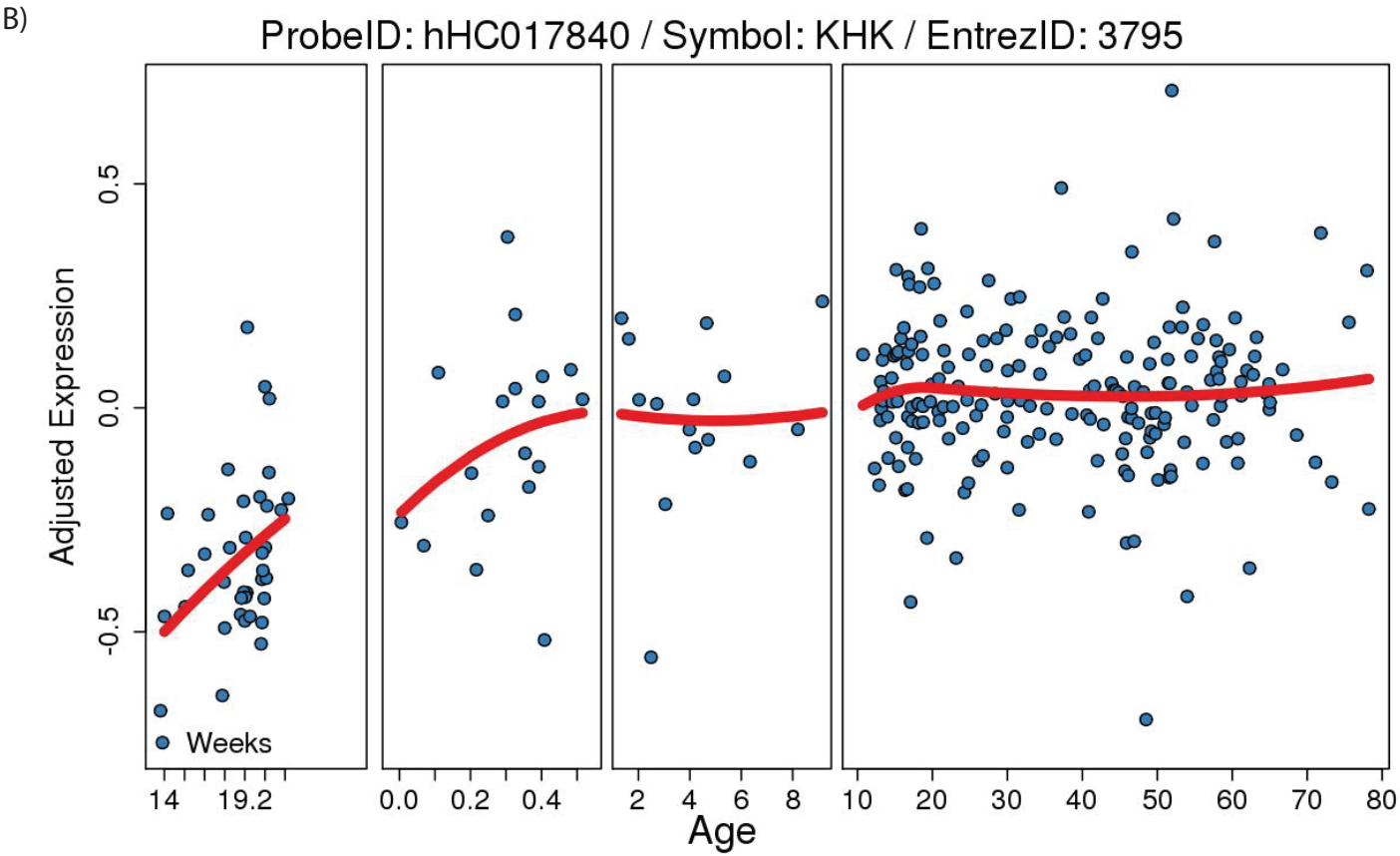

C)

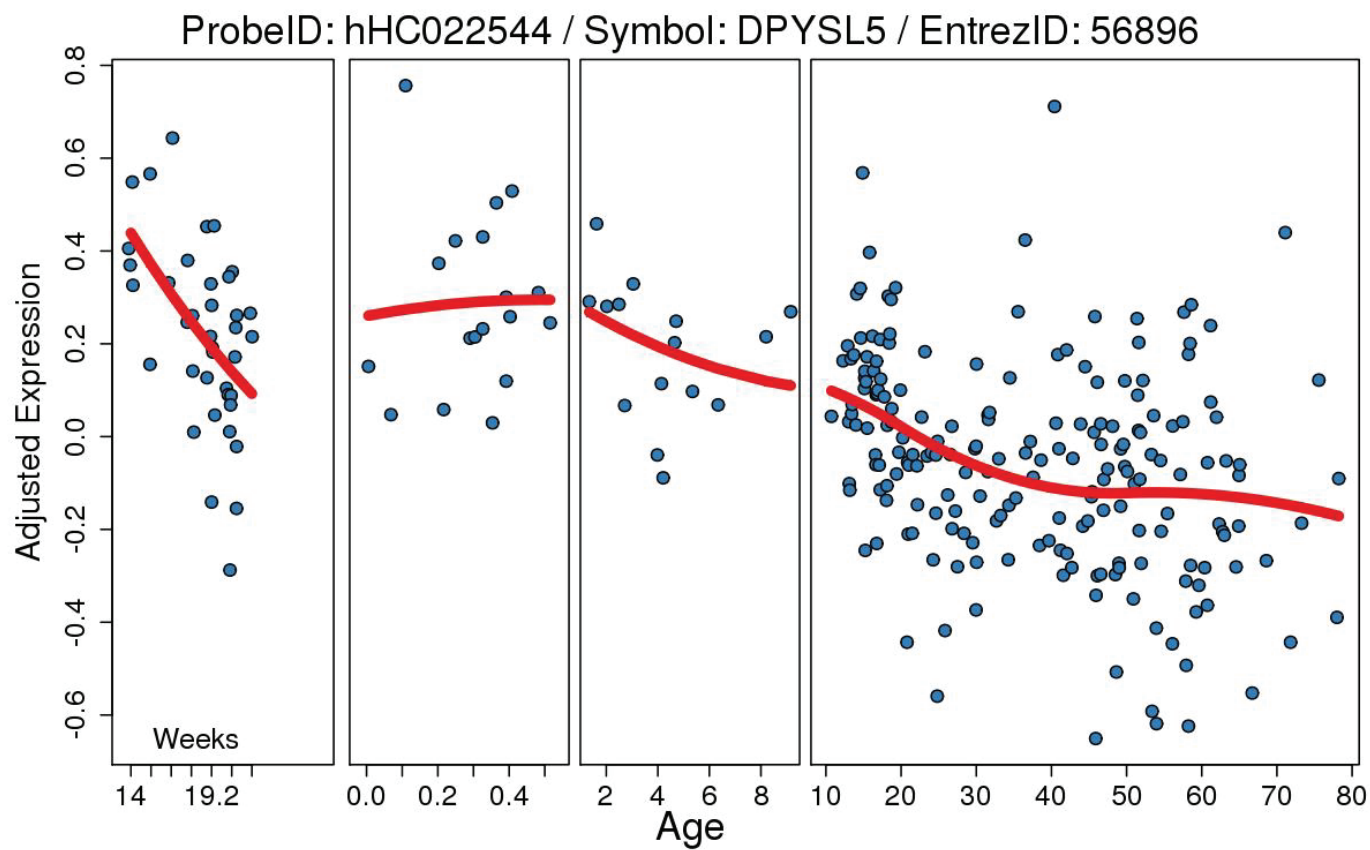

D)

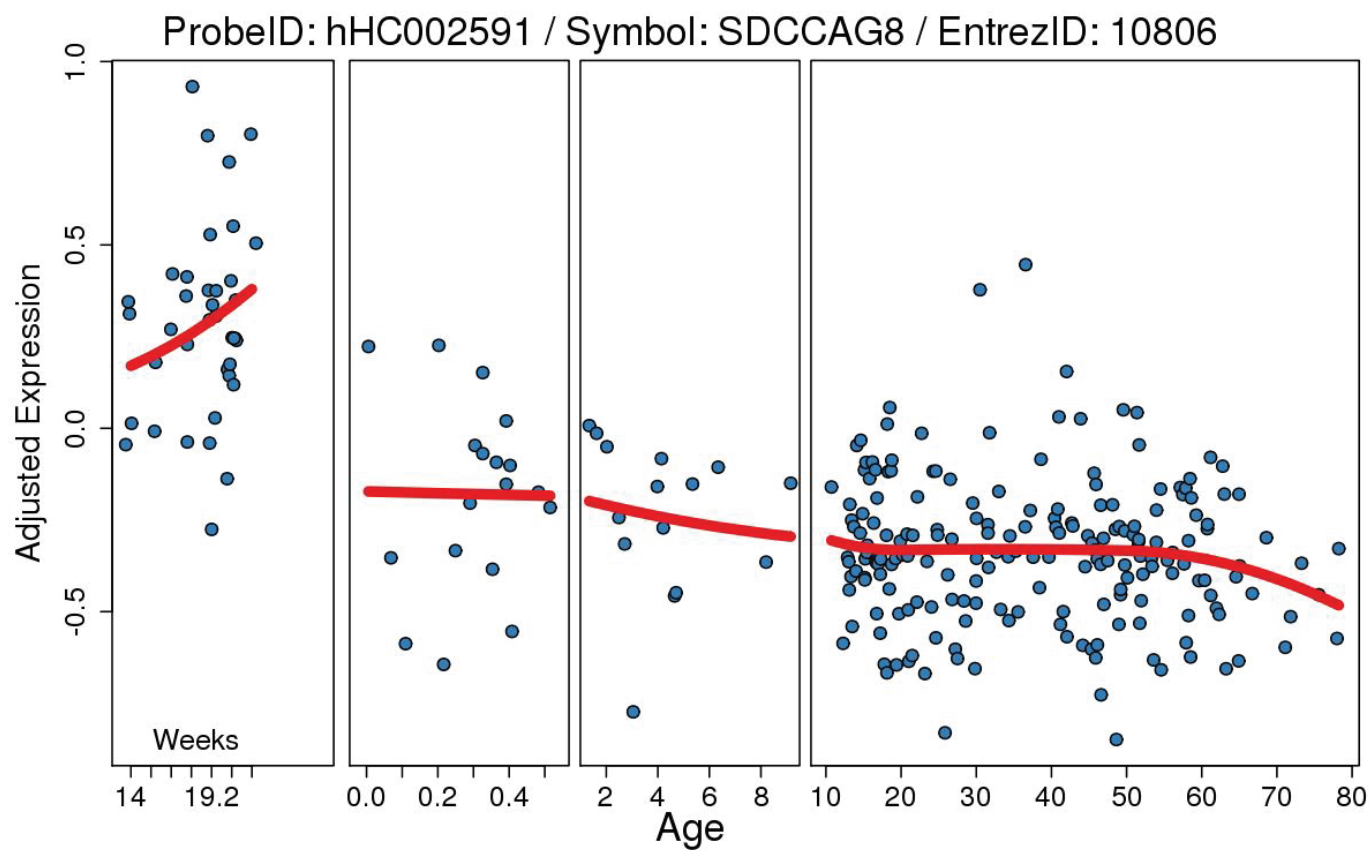

E)

ProbeID: hHR021371 / Symbol: C15orf59 / EntrezID: 388135

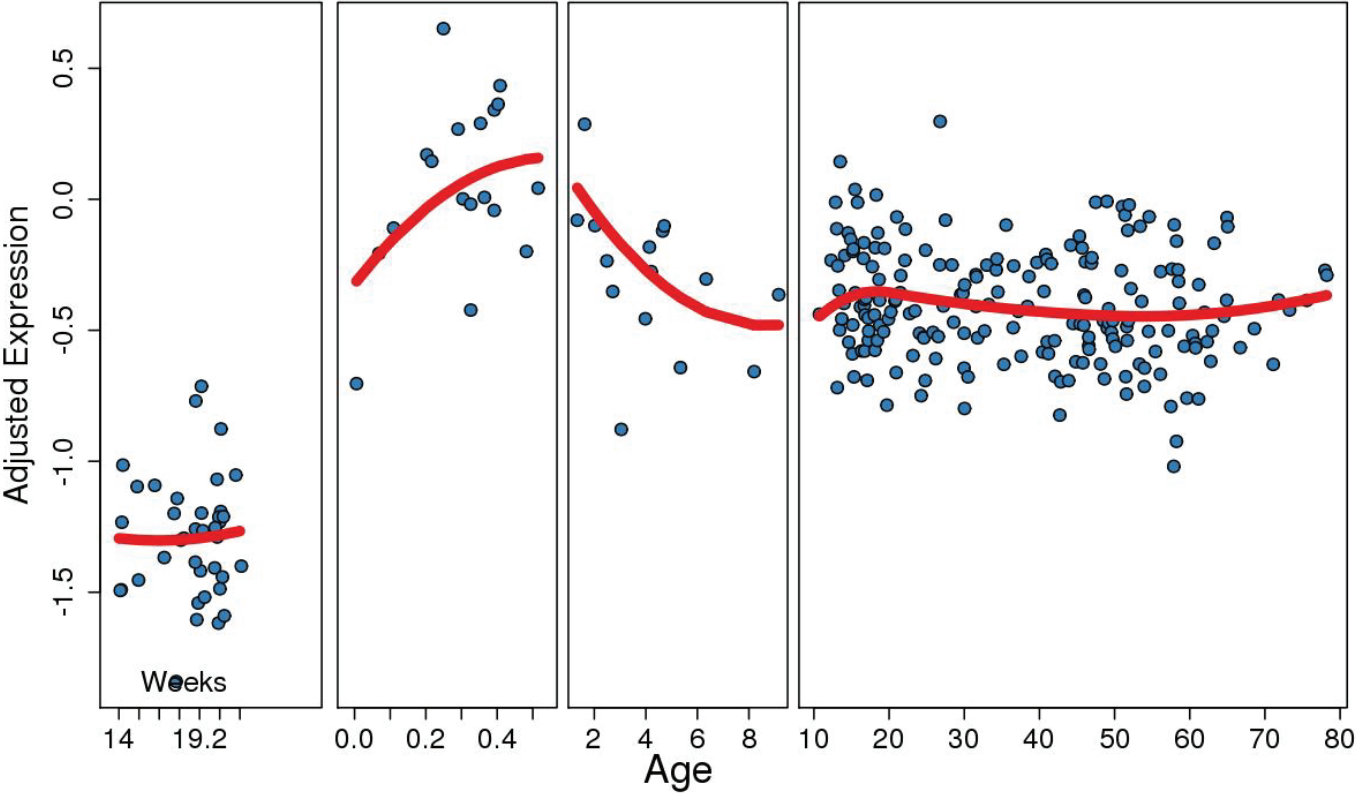

Supplement: Supplementary file 7 — Supplementary Figure 6 [file 41398_2018_236_MOESM7_ESM.pdf]
