# Supplementary material for: Genetics of self-reported risk-taking behaviour, trans-ethnic consistency and relevance to brain gene expression
Source: Transl Psychiatry. 2018 Sep 4;8:178. doi: 10.1038/s41398-018-0236-1 (PMC6123450; doi:10.1038/s41398-018-0236-1)
Supplement: Supplementary file 10 — Supplementary Table 3 [file 41398_2018_236_MOESM10_ESM.docx]

Supplemental Table 3: Lead SNPs in risk-taking associated loci

| CHR | BP | SNP | Closest Gene | Analysis | A1 | A2 | MAF | N | BETA | SE | L95 | U95 | P | N SNPs |
| --- | --- | --- | --- | --- | --- | --- | --- | --- | --- | --- | --- | --- | --- | --- |
| 1 | 243812368 | rs560977020 | *AKT3* | all | C | T | 0.32 | 323626 | -0.035 | 0.006 | -0.047 | -0.023 | **2.04E-08** | 11 |
| 2 | 27315252 | rs2304681 | *KHK* | all | A | G | 0.37 | 328339 | -0.033 | 0.006 | -0.045 | -0.022 | **2.58E-08** | 12 |
| 3 | 85617378 | rs542809491 | *CADM2* | all | A | T | 0.38 | 300100 | 0.056 | 0.006 | 0.044 | 0.069 | **1.02E-19** | 812 |
| 3 | 181408124 | rs9841382 | *SOX2* | all | C | T | 0.14 | 327162 | 0.055 | 0.008 | 0.039 | 0.071 | **2.26E-11** | 42 |
| 3 | 181420180 | rs75907840^a^ |  | all | G | T | 0.21 | 327136 | -0.042 | 0.007 |  |  | **4.26E-09** |  |
| 6 | 27766842 | rs188973463 | *POM121L2* | all | G | T | 0.26 | 316407 | -0.042 | 0.007 | -0.055 | -0.029 | **5.16E-10** | 2 |
|  | 29230129 | rs566858049 | *OR14J1* | all | C | T | 0.39 | 324739 | -0.036 | 0.006 | -0.047 | -0.024 | **2.00E-09** | 26 |
| 7 | 114156758 | 7:114156758 | *FOXP2* | all | GT | G | 0.36 | 297228 | -0.040 | 0.006 | -0.053 | -0.028 | **2.01E-10** | 31 |
|  | 114109349 | rs727644^b^ |  | all | A | G | 0.40 | 325370 | -0.036 | 0.006 | -0.048 | -0.025 | **8.99E-10** |  |
| 8 | 65508415 | rs189335278 | *CYP7B1* | all | A | T | 0.11 | 326189 | -0.053 | 0.009 | -0.071 | -0.035 | **1.03E-08** | 7 |
| 11 | 104700736 | 11:104700736 | *CASP12* | all | A | ACTTCAC | 0.25 | 309973 | 0.039 | 0.007 | 0.026 | 0.053 | **7.59E-09** | 208 |
|  | 104700048 | rs10895735^b^ |  | all | G | A | 0.24 | 326950 | 0.039 | 0.007 | 0.025 | 0.052 | **1.17E-08** |  |
| 15 | 74064198 | rs545973460 | *C15orf59* | all | A | G | 0.35 | 324177 | 0.034 | 0.006 | 0.022 | 0.046 | **2.16E-08** | 2 |
| 16 | 69550486 | rs145206681 | *NFAT5* | all | T | C | 0.06 | 320781 | 0.067 | 0.012 | 0.043 | 0.090 | **2.47E-08** | 5 |
| 3 | 85513793 | rs62250713 | *CADM2* | men | A | G | 0.36 | 151957 | 0.060 | 0.008 | 0.045 | 0.076 | **3.42E-14** | 795 |
| 12 | 24237747 | rs10505932 | *SOX5* | men | G | T | 0.26 | 149255 | 0.049 | 0.009 | 0.032 | 0.066 | **2.81E-08** | 1 |
| 1 | 243458502 | rs3943093 | *AKT3* | women | T | C | 0.32 | 175362 | -0.052 | 0.009 | -0.070 | -0.033 | **4.33E-08** | 1 |
| 3 | 85617378 | rs542809491 | *CADM2* | women | A | T | 0.38 | 160803 | 0.052 | 0.009 | 0.034 | 0.070 | **3.19E-08** | 2 |
| 8 | 65508415 | rs189335278 | *CYP7B1* | women | A | T | 0.11 | 174824 | -0.084 | 0.014 | -0.112 | -0.056 | **2.98E-09** | 20 |
| 10 | 8784773 | rs11255890 | *RP11-428L9.1* | women | C | A | 0.38 | 171862 | 0.051 | 0.009 | 0.034 | 0.069 | **1.48E-08** | 51 |
| 15 | 74060078 | rs17187323 | *C15orf59* | women | A | T | 0.35 | 172736 | 0.053 | 0.009 | 0.035 | 0.071 | **7.90E-09** | 4 |
| Where: N SNPs, number of GWAS significant SNPs; N signals, number of signals in the locus; ^a^ suggestive secondary signal; ^b^ a proxy with an rsid, used for data-mining | | | | | | | | | | | | | | |
