# Supplementary material for: Genetics of self-reported risk-taking behaviour, trans-ethnic consistency and relevance to brain gene expression
Source: Transl Psychiatry. 2018 Sep 4;8:178. doi: 10.1038/s41398-018-0236-1 (PMC6123450; doi:10.1038/s41398-018-0236-1)
Supplement: Supplementary file 11 — Supplementary Table 4 [file 41398_2018_236_MOESM11_ESM.docx]

Supplemental Table 4: conditional analysis of the CADM2 locus

|  |  |  |  |  |  |  | age, sex, chip, population structure | | | age, sex, chip, population structure, rs542809491 | | | age, sex, chip, population structure, rs13084531 | | |
| --- | --- | --- | --- | --- | --- | --- | --- | --- | --- | --- | --- | --- | --- | --- | --- |
| CHR | SNP | BP | A1 | A2 | MAF | NMISS | BETA | SE | P | BETA | SE | P | BETA | SE | P |
| 3 | rs542809491 | 85617378 | A | T | 0.38 | 300100 | 0.056 | 0.006 | **1.02E-19** |  |  |  | 0.051 | 0.009 | **6.16E-09** |
| 3 | rs13084531 | 85553994 | G | C | 0.23 | 327728 | 0.050 | 0.007 | **4.21E-13** | 0.009 | 0.010 | 3.81E-01 |  |  |  |
| 3 | rs75892230 | 85506121 | A | C | 0.06 | 323164 | -0.067 | 0.012 | **3.54E-08** | -0.045 | 0.013 | 4.90E-04 | -0.055 | 0.012 | 7.26E-06 |
| Where: rs542809491, new lead SNP; rs13084531, old lead SNP; rs75892230, most significant SNP after conditioning on the new lead SNP. | | | | | | | | | | | | | | | |
