# Supplementary material for: Genetics of self-reported risk-taking behaviour, trans-ethnic consistency and relevance to brain gene expression
Source: Transl Psychiatry. 2018 Sep 4;8:178. doi: 10.1038/s41398-018-0236-1 (PMC6123450; doi:10.1038/s41398-018-0236-1)
Supplement: Supplementary file 12 — Supplementary Table 5 [file 41398_2018_236_MOESM12_ESM.docx]

Supplemental Table 5: Conditional analysis of the HLA region

|  |  |  |  |  | age, sex, chip, population structure | | | age, sex, chip, population structure, rs9379971 | | | age, sex, chip, population structure, rs188973463 | | | age, sex, chip, population structure, rs566858049 | | |
| --- | --- | --- | --- | --- | --- | --- | --- | --- | --- | --- | --- | --- | --- | --- | --- | --- |
| CHR | SNP | A1 | A2 | MAF | BETA | SE | P | BETA | SE | P | BETA | SE | P | BETA | SE | P |
| **6** | **rs2393923** | **C** | **T** | **0.32** | **-0.034** | **0.006** | **4.64E-08** | -0.031 | 0.009 | 5.59E-04 | -0.021 | 0.007 | 2.97E-03 | -0.026 | 0.007 | 7.06E-05 |
| **6** | **rs188973463** | **G** | **T** | **0.26** | **-0.042** | **0.007** | **5.16E-10** | -0.036 | 0.007 | 6.52E-07 |  |  |  | -0.031 | 0.008 | 8.31E-05 |
| **6** | **rs3130807** | **A** | **G** | **0.38** | **-0.034** | **0.006** | **1.16E-08** | -0.028 | 0.007 | 1.81E-05 | -0.020 | 0.007 | 4.32E-03 | 0.004 | 0.025 | 8.76E-01 |
| **6** | **rs2207338** | **G** | **T** | **0.38** | **-0.034** | **0.006** | **8.25E-09** | -0.029 | 0.007 | 1.31E-05 | -0.020 | 0.007 | 3.78E-03 | 0.003 | 0.029 | 9.08E-01 |
| **6** | **rs2207337** | **T** | **A** | **0.38** | **-0.034** | **0.006** | **8.88E-09** | -0.029 | 0.007 | 1.39E-05 | -0.020 | 0.007 | 4.15E-03 | 0.003 | 0.027 | 9.26E-01 |
| **6** | **rs567676249** | **G** | **A** | **0.38** | **-0.034** | **0.006** | **8.25E-09** | -0.029 | 0.007 | 1.31E-05 | -0.020 | 0.007 | 3.78E-03 | 0.003 | 0.029 | 9.08E-01 |
| **6** | **rs3117340** | **T** | **G** | **0.38** | **-0.035** | **0.006** | **6.11E-09** | -0.029 | 0.007 | 9.45E-06 | -0.020 | 0.007 | 3.47E-03 | -0.007 | 0.027 | 7.92E-01 |
| **6** | **rs3130821** | **C** | **T** | **0.38** | **-0.034** | **0.006** | **9.44E-09** | -0.029 | 0.007 | 1.41E-05 | -0.020 | 0.007 | 4.05E-03 | 0.007 | 0.028 | 8.16E-01 |
| **6** | **rs2064503** | **T** | **C** | **0.38** | **-0.034** | **0.006** | **9.94E-09** | -0.029 | 0.007 | 1.41E-05 | -0.020 | 0.007 | 4.32E-03 | 0.001 | 0.026 | 9.65E-01 |
| **6** | **rs2207336** | **G** | **A** | **0.38** | **-0.034** | **0.006** | **9.92E-09** | -0.029 | 0.007 | 1.51E-05 | -0.020 | 0.007 | 3.82E-03 | 0.007 | 0.028 | 8.04E-01 |
| **6** | **rs3117331** | **A** | **G** | **0.38** | **-0.034** | **0.006** | **7.84E-09** | -0.029 | 0.007 | 1.28E-05 | -0.020 | 0.007 | 3.41E-03 | 0.004 | 0.030 | 8.86E-01 |
| **6** | **rs3117330** | **T** | **C** | **0.38** | **-0.035** | **0.006** | **5.56E-09** | -0.029 | 0.007 | 9.42E-06 | -0.021 | 0.007 | 2.85E-03 | -0.003 | 0.029 | 9.23E-01 |
| **6** | **rs566858049** | **C** | **T** | **0.39** | **-0.036** | **0.006** | **2.00E-09** | -0.030 | 0.007 | 5.89E-06 | -0.022 | 0.007 | 1.58E-03 |  |  |  |
| **6** | **rs3130827** | **T** | **C** | **0.38** | **-0.035** | **0.006** | **5.22E-09** | -0.029 | 0.007 | 8.87E-06 | -0.021 | 0.007 | 2.77E-03 | -0.005 | 0.029 | 8.64E-01 |
| **6** | **rs576286578** | **A** | **C** | **0.38** | **-0.035** | **0.006** | **5.22E-09** | -0.029 | 0.007 | 8.87E-06 | -0.021 | 0.007 | 2.77E-03 | -0.005 | 0.029 | 8.64E-01 |
| **6** | **rs3117347** | **G** | **T** | **0.38** | **-0.035** | **0.006** | **5.61E-09** | -0.029 | 0.007 | 1.04E-05 | -0.021 | 0.007 | 2.58E-03 | -0.007 | 0.028 | 7.97E-01 |
| **6** | **rs3117346** | **T** | **C** | **0.38** | **-0.035** | **0.006** | **6.52E-09** | -0.029 | 0.007 | 1.24E-05 | -0.021 | 0.007 | 2.70E-03 | -0.002 | 0.029 | 9.46E-01 |
| **6** | **rs144567770** | **T** | **C** | **0.38** | **-0.035** | **0.006** | **6.42E-09** | -0.029 | 0.007 | 1.12E-05 | -0.021 | 0.007 | 3.09E-03 | -0.002 | 0.029 | 9.47E-01 |
| **6** | **rs7381760** | **G** | **C** | **0.38** | **-0.035** | **0.006** | **3.94E-09** | -0.029 | 0.007 | 1.02E-05 | -0.021 | 0.007 | 2.45E-03 | -0.006 | 0.029 | 8.29E-01 |
| **6** | **rs182948268** | **G** | **A** | **0.38** | **-0.034** | **0.006** | **8.14E-09** | -0.029 | 0.007 | 1.17E-05 | -0.021 | 0.007 | 2.75E-03 | -0.002 | 0.029 | 9.52E-01 |
| **6** | **rs535176959** | **G** | **C** | **0.38** | **-0.034** | **0.006** | **8.14E-09** | -0.029 | 0.007 | 1.17E-05 | -0.021 | 0.007 | 2.75E-03 | -0.002 | 0.029 | 9.52E-01 |
| **6** | **rs1884123** | **A** | **G** | **0.39** | **-0.034** | **0.006** | **7.92E-09** | -0.029 | 0.007 | 7.64E-06 | -0.021 | 0.007 | 2.85E-03 | -0.006 | 0.020 | 7.56E-01 |
| **6** | **rs6925072** | **A** | **G** | **0.38** | **-0.034** | **0.006** | **9.87E-09** | -0.029 | 0.007 | 7.26E-06 | -0.020 | 0.007 | 3.38E-03 | -0.008 | 0.017 | 6.20E-01 |
| **6** | **rs4711183** | **G** | **A** | **0.38** | **-0.034** | **0.006** | **1.06E-08** | -0.029 | 0.007 | 8.00E-06 | -0.020 | 0.007 | 3.76E-03 | -0.007 | 0.017 | 6.55E-01 |
| **6** | **rs41264409** | **T** | **TCA** | **0.39** | **-0.034** | **0.006** | **7.92E-09** | -0.029 | 0.007 | 7.64E-06 | -0.021 | 0.007 | 2.85E-03 | -0.006 | 0.020 | 7.56E-01 |
| **6** | **rs2394552** | **T** | **C** | **0.38** | **-0.035** | **0.006** | **5.61E-09** | -0.030 | 0.007 | 4.88E-06 | -0.021 | 0.007 | 2.27E-03 | -0.011 | 0.017 | 5.14E-01 |
| **6** | **rs532419719** | **A** | **G** | **0.38** | **-0.034** | **0.006** | **8.76E-09** | -0.029 | 0.007 | 6.43E-06 | -0.020 | 0.007 | 3.47E-03 | -0.009 | 0.017 | 5.95E-01 |
| **6** | **rs548753328** | **T** | **G** | **0.38** | **-0.034** | **0.006** | **1.14E-08** | -0.029 | 0.007 | 8.77E-06 | -0.020 | 0.007 | 4.05E-03 | -0.007 | 0.017 | 6.75E-01 |
| Where: rs9379971, previously reported lead SNP; rs188973463, lead SNP at Chr6:27Mb; rs566858049, lead SNP at Chr6:29Mb | | | | | | | | | | | | | | | | |
