# Supplementary material for: Genetics of self-reported risk-taking behaviour, trans-ethnic consistency and relevance to brain gene expression
Source: Transl Psychiatry. 2018 Sep 4;8:178. doi: 10.1038/s41398-018-0236-1 (PMC6123450; doi:10.1038/s41398-018-0236-1)
Supplement: Supplementary file 13 — Supplementary Table 6 [file 41398_2018_236_MOESM13_ESM.docx]

Supplemental Table 6: conditional analysis of the SOX2 locus

|  |  |  |  |  |  | age, sex, chip, population structure | | | age, sex, chip, population structure, rs9841382 | | | age, sex, chip, population structure, rs75907840 | | |
| --- | --- | --- | --- | --- | --- | --- | --- | --- | --- | --- | --- | --- | --- | --- |
| CHR | SNP | A1 | A2 | MAF | NMISS | BETA | SE | P | BETA | SE | P | BETA | SE | P |
| 3 | rs4855035 | G | T | 0.15 | 325606 | 0.046 | 0.008 | **1.56E-08** | 0.000 | 0.015 | 9.80E-01 | 0.039 | 0.008 | 3.23E-06 |
| 3 | rs4133078 | G | A | 0.13 | 325833 | 0.048 | 0.009 | **2.38E-08** | -0.017 | 0.018 | 3.48E-01 | 0.040 | 0.009 | 3.14E-06 |
| 3 | rs11924735 | T | C | 0.17 | 327995 | 0.042 | 0.008 | **2.97E-08** | 0.006 | 0.012 | 6.05E-01 | 0.035 | 0.008 | 1.00E-05 |
| 3 | rs11927844 | T | C | 0.17 | 328128 | 0.042 | 0.008 | **2.93E-08** | 0.006 | 0.012 | 5.96E-01 | 0.035 | 0.008 | 1.01E-05 |
| 3 | rs6804544 | C | G | 0.17 | 328339 | 0.042 | 0.008 | **2.94E-08** | 0.006 | 0.012 | 5.88E-01 | 0.035 | 0.008 | 9.96E-06 |
| 3 | rs149234347 | G | A | 0.17 | 328339 | 0.042 | 0.008 | **2.94E-08** | 0.006 | 0.012 | 5.88E-01 | 0.035 | 0.008 | 9.96E-06 |
| 3 | rs145482598 | T | A | 0.13 | 324780 | 0.050 | 0.009 | **5.74E-09** | -0.010 | 0.019 | 5.97E-01 | 0.042 | 0.009 | 9.87E-07 |
| 3 | rs9844617 | A | C | 0.20 | 322094 | 0.042 | 0.007 | **4.93E-09** | 0.012 | 0.012 | 3.34E-01 | 0.034 | 0.008 | 5.35E-06 |
| 3 | rs35364893 | G | A | 0.14 | 325068 | 0.054 | 0.008 | **6.66E-11** | -0.034 | 0.078 | 6.68E-01 | 0.046 | 0.008 | 5.23E-08 |
| 3 | rs9841382 | C | T | 0.14 | 327162 | 0.055 | 0.008 | **2.26E-11** |  |  |  | 0.047 | 0.008 | **2.27E-08** |
| 3 | rs9841842 | A | T | 0.15 | 327109 | 0.053 | 0.008 | **2.82E-11** | 0.022 | 0.041 | 5.85E-01 | 0.046 | 0.008 | **3.28E-08** |
| 3 | rs190826351 | T | C | 0.17 | 318644 | 0.043 | 0.008 | **2.67E-08** | 0.009 | 0.013 | 4.93E-01 | 0.035 | 0.008 | 9.74E-06 |
| 3 | rs7631379 | C | T | 0.21 | 328339 | 0.041 | 0.007 | **7.50E-09** | 0.010 | 0.012 | 4.25E-01 | 0.033 | 0.007 | 5.66E-06 |
| 3 | rs7619688 | G | C | 0.21 | 327898 | 0.041 | 0.007 | **4.79E-09** | 0.009 | 0.012 | 4.32E-01 | 0.034 | 0.007 | 4.09E-06 |
| 3 | rs181804999 | T | A | 0.16 | 318104 | 0.045 | 0.008 | **9.05E-09** | 0.010 | 0.014 | 4.55E-01 | 0.038 | 0.008 | 3.76E-06 |
| 3 | rs11921361 | G | C | 0.14 | 327049 | 0.054 | 0.008 | **5.45E-11** | -0.179 | 0.383 | 6.40E-01 | 0.046 | 0.008 | **4.85E-08** |
| 3 | rs7643765 | C | T | 0.14 | 327053 | 0.054 | 0.008 | **5.20E-11** | -0.195 | 0.299 | 5.14E-01 | 0.046 | 0.008 | **4.65E-08** |
| 3 | rs7632216 | A | G | 0.14 | 327057 | 0.053 | 0.008 | **5.94E-11** | -0.290 | 0.290 | 3.17E-01 | 0.046 | 0.008 | 5.29E-08 |
| 3 | rs13060544 | G | A | 0.14 | 326829 | 0.053 | 0.008 | **1.18E-10** | -0.042 | 0.078 | 5.88E-01 | 0.045 | 0.008 | 7.94E-08 |
| 3 | rs4129750 | G | A | 0.15 | 326956 | 0.052 | 0.008 | **1.32E-10** | 0.017 | 0.039 | 6.61E-01 | 0.044 | 0.008 | 1.17E-07 |
| 3 | rs4306901 | T | A | 0.14 | 327096 | 0.054 | 0.008 | **4.87E-11** | 0.117 | 0.230 | 6.10E-01 | 0.046 | 0.008 | **4.53E-08** |
| 3 | rs4510419 | C | G | 0.20 | 327010 | 0.042 | 0.007 | **3.80E-09** | 0.012 | 0.012 | 3.35E-01 | 0.034 | 0.007 | 5.76E-06 |
| 3 | rs6809817 | G | T | 0.14 | 327532 | 0.055 | 0.008 | **2.27E-11** | 0.196 | 0.141 | 1.64E-01 | 0.047 | 0.008 | **2.38E-08** |
| 3 | rs191667545 | G | A | 0.14 | 320061 | 0.053 | 0.008 | **3.28E-10** | -0.008 | 0.050 | 8.66E-01 | 0.045 | 0.009 | 1.45E-07 |
| 3 | rs13096623 | A | G | 0.14 | 328339 | 0.054 | 0.008 | **2.71E-11** | 0.077 | 0.083 | 3.53E-01 | 0.047 | 0.008 | **2.58E-08** |
| 3 | rs34308817 | T | G | 0.12 | 323454 | 0.052 | 0.009 | **7.44E-09** | -0.010 | 0.021 | 6.25E-01 | 0.045 | 0.009 | 1.25E-06 |
| 3 | rs578243885 | C | A | 0.20 | 319482 | 0.042 | 0.007 | **5.73E-09** | 0.010 | 0.013 | 4.19E-01 | 0.033 | 0.008 | 9.03E-06 |
| 3 | rs114600294^a^ | C | G | 0.21 | 328339 | -0.041 | 0.007 | **1.06E-08** | -0.032 | 0.007 | 1.17E-05 | 0.020 | 0.058 | 7.29E-01 |
| 3 | rs9814658 | G | A | 0.14 | 327852 | 0.053 | 0.008 | **6.58E-11** | 0.042 | 0.085 | 6.21E-01 | 0.046 | 0.008 | **4.99E-08** |
| 3 | rs75907840^a^ | G | T | 0.21 | 327136 | -0.042 | 0.007 | **4.26E-09** | -0.033 | 0.007 | 7.02E-06 |  |  |  |
| 3 | rs9290727 | C | T | 0.15 | 327350 | 0.053 | 0.008 | **5.88E-11** | 0.021 | 0.037 | 5.72E-01 | 0.045 | 0.008 | 5.40E-08 |
| 3 | rs541189464 | G | A | 0.21 | 328339 | 0.041 | 0.007 | **7.50E-09** | 0.010 | 0.012 | 4.25E-01 | 0.033 | 0.007 | 5.66E-06 |
| 3 | rs4434184 | G | A | 0.18 | 326240 | 0.048 | 0.007 | **2.20E-10** | 0.022 | 0.015 | 1.55E-01 | 0.040 | 0.008 | 2.72E-07 |
| 3 | rs12487748 | A | T | 0.14 | 325788 | 0.055 | 0.008 | **3.75E-11** | -0.001 | 0.056 | 9.83E-01 | 0.047 | 0.008 | **2.70E-08** |
| 3 | rs12497248 | G | C | 0.14 | 325686 | 0.055 | 0.008 | **3.41E-11** | 0.020 | 0.066 | 7.65E-01 | 0.047 | 0.008 | **2.56E-08** |
| 3 | rs534378299 | G | C | 0.18 | 301985 | 0.044 | 0.008 | **2.19E-08** | -0.013 | 0.018 | 4.49E-01 | 0.041 | 0.008 | 1.38E-07 |
| 3 | rs35788479 | A | G | 0.14 | 316977 | 0.056 | 0.008 | **6.28E-11** | 0.016 | 0.066 | 8.11E-01 | 0.048 | 0.009 | **3.07E-08** |
| 3 | rs558923056 | C | A | 0.14 | 328339 | 0.054 | 0.008 | **2.71E-11** | 0.077 | 0.083 | 3.53E-01 | 0.047 | 0.008 | **2.58E-08** |
| 3 | rs574725004 | C | T | 0.17 | 311837 | 0.046 | 0.008 | **2.81E-09** | -0.007 | 0.019 | 7.03E-01 | 0.038 | 0.008 | 2.42E-06 |
| 3 | rs535691787 | A | G | 0.14 | 327494 | 0.054 | 0.008 | **4.20E-11** | 0.086 | 0.089 | 3.34E-01 | 0.046 | 0.008 | **3.23E-08** |
| 3 | rs77716014 | T | C | 0.14 | 320389 | 0.055 | 0.008 | **4.10E-11** | -0.064 | 0.056 | 2.52E-01 | 0.048 | 0.009 | **2.75E-08** |
| 3 | rs541515998^a^ | C | G | 0.21 | 328339 | -0.041 | 0.007 | **1.06E-08** | -0.032 | 0.007 | 1.17E-05 | 0.020 | 0.058 | 7.29E-01 |
| 3 | rs142839329 | A | G | 0.14 | 327210 | 0.054 | 0.008 | **3.99E-11** | 0.071 | 0.080 | 3.74E-01 | 0.046 | 0.008 | **3.06E-08** |
| 3 | rs534455770 | T | C | 0.14 | 326340 | 0.054 | 0.008 | **3.91E-11** | 0.043 | 0.072 | 5.47E-01 | 0.047 | 0.008 | **2.70E-08** |
| 3 | 3:181439915_AT_A | A | AT | 0.17 | 310534 | 0.049 | 0.008 | **2.31E-10** | 0.025 | 0.016 | 1.31E-01 | 0.042 | 0.008 | 1.89E-07 |
| Where: ^a^, possible secondary signal | | | | | |  |  |  |  |  |  |  |  |  |
