# Supplementary material for: Genetics of self-reported risk-taking behaviour, trans-ethnic consistency and relevance to brain gene expression
Source: Transl Psychiatry. 2018 Sep 4;8:178. doi: 10.1038/s41398-018-0236-1 (PMC6123450; doi:10.1038/s41398-018-0236-1)
Supplement: Supplementary file 15 — Supplementary Table 8 [file 41398_2018_236_MOESM15_ESM.docx]

Supplemental Table 8: Demographics of individuals included in the analysis of additional ethnicities.

|  | white non-British | | South Asian | | African-Caribbean | | Mixed | |
| --- | --- | --- | --- | --- | --- | --- | --- | --- |
|  | controls | risk-takers^a^ | controls | risk-takers^a^ | controls | risk-takers^a^ | controls | risk-takers^a^ |
| N | 31814 (66.3) | 16169 (33.7) | 4267 (60.7) | 2764 (39.3) | 4341 (58.0) | 3139 (42.0) | 5988 (60.8) | 3866 (39.2) |
| Men | 15558 (38.1) | 10259 (53.3) | 2168 (50.8) | 1607 (58.1) | 1659 (38.2) | 1502 (47.9) | 2318 (38.7) | 1862 (48.2) |
| Age (years) | 56.0 (8.1) | 55.0 (8.2) | 53.7 (8.5) | 52.8 (8.4) | 52.6 (8.2) | 51.1 (7.8) | 52.9 (8.1) | 51.8 (7.9) |
| BMI (kg/m2) | 27.1 (4.9) | 27.4 (4.80) | 27.1 (4.4) | 27.4 (4.5) | 29.4 (5.5) | 29.6 (3.4) | 26.8 (5.0) | 27.3 (4.8) |
| Current smoker | 14749 (36.2) | 7639 (39.8) | 477 (11.3) | 370 (13.5) | 777 (18.0) | 521 (16.7) | 1395 (23.4) | 1045 (27.2) |
| Ever smoker | 19076 (46.8) | 10918 (56.9) | 779 (18.4) | 693 (25.3) | 1254 (29.1) | 968 (31.0) | 2090 (35.1) | 1716 (44.6) |
| Age completed education^b^ | 16.9 (2.4) | 17.0 (2.7) | 18.2 (3.3) | 18.4 (3.4) | 18.3 (4.1) | 19.0 (4.4) | 18.2 (3.6) | 18.5 (3.8) |
| Has a degree | 14701 (38.5) | 8584 (47.1) | 1578 (39.2) | 1232 (46.6) | 1231 (29.6) | 1215 (39.7) | 2502 (43.4) | 1872 (50.0) |
| Townsend deprivation index | -0.84 (3.24) | -0.35 (3.42) | 0.14 (3.09) | 0.14 (3.13) | 2.52 (3.46) | 2.70 (3.42) | 0.53 (3.60) | 0.97 (3.62) |
| Unstable mood^c^ | 17364 (43.6) | 9133 (48.5) | 1948 (49.0) | 1470 (55.7) | 2066 (50.8) | 1606 (53.6) | 2613 (45.8) | 1943 (52.4) |
| Probable mood phenotyping^d^ | 11377 | 5261 | 1850 | 1158 | 1801 | 1267 | 2136 | 1285 |
| Comparison group^d^ | 8474 (74.5) | 3550 (67.5) | 1568 (84.8) | 922 (79.6) | 1491 (82.8) | 1005 (79.3) | 1713 (80.2) | 915 (71.2) |
| BD^d^ | 146 (1.3) | 153 (2.9) | 23 (1.2) | 31 (2.7) | 27 (1.5) | 25 (2.0) | 31 (1.5) | 38 (3.0) |
| single episode depression^d^ | 696 (6.1) | 318 (6.0) | 50 (2.7) | 31 (2.7) | 52 (2.9) | 51 (4.0) | 71 (3.3) | 56 (4.4) |
| Moderate depression^d^ | 1222 (10.7) | 673 (12.8) | 118 (6.4) | 84 (7.3) | 134 (7.4) | 102 (8.1) | 197 (9.2) | 146 (11.4) |
| Severe depression^d^ | 839 (7.4) | 567 (10.8) | 91 (4.9) | 90 (7.8) | 97 (5.4) | 84 (6.6) | 124 (5.8) | 130 (10.1) |
| any depression | 2757 (24.2) | 1558 (29.6) | 259 (14.0) | 205 (17.7) | 283 (15.7) | 237 (18.7) | 392 (18.4) | 332 (25.8) |
| Mental Health Questionnaire | 12852 | 6225 | 601 | 405 | 628 | 458 | 1331 | 864 |
| BD | 178 (1.4) | 186 (3.0) | 17 (2.9) | 10 (2.5) | 14 (2.3) | 11 (2.5) | 21 (1.6) | 29 (3.4) |
| MDD | 3090 (28.8) | 1674 (32.3) | 118 (26.7) | 74 (23.1) | 110 (21.8) | 90 (23.1) | 316 (28.8) | 211 (30.0) |
| GAD | 928 (10.8) | 530 (12.8) | 37 (9.1) | 38 (13.5) | 29 (6.3) | 26 (7.8) | 102 (11.1) | 60 (10.7) |
| any addiction | 736 (5.8) | 670 (10.9) | 18 (3.0) | 27 (6.7) | 25 (4.0) | 39 (8.6) | 77 (5.8) | 82 (9.7) |
| alcoholism | 270 (2.1) | 233 (3.8) | 5 (0.8) | 8 (2.0) | 11 (1.8) | 11 (2.4) | 25 (1.9) | 25 (3.0) |
| illicit drug addiction | 70 (0.6) | 109 (1.8) | 1 (0.2) | 5 (1.2) | 3 (0.5) | 10 (2.2) | 12 (0.9) | 20 (2.37) |
| OTC/prescription addiction | 104 (0.8) | 80 (1.3) | 3 (0.5) | 5 (1.2) | 1 (0.02) | 7 (1.5) | 11 (0.8) | 6 (0.7) |
| Ever cannabis | 3409 (27.0) | 2459 (39.6) | 65 (10.8) | 62 (15.3) | 133 (21.2) | 115 (25.2) | 324 (24.4) | 278 (32.3) |
| Where: ^a^ participants who answered "yes" to "do you consider yourself a risk taker?"; ^b^ based on a subset of 80 229 subjects; ^c^ Unstable mood, defined by the question ""Does your mood often go up and down?"; Paticipants who answered yes were classified as having unstable mood ; ^d^ definitions as per Smith et al, Plos One, 2013; BD, bipolar disorder; MDD, major depressive disorder; GAD, generalised anxiety disorder; OTC, over the counter. Addiction phenotypes based on self-report. | | | | | | | | |
