# Supplementary material for: Genetics of self-reported risk-taking behaviour, trans-ethnic consistency and relevance to brain gene expression
Source: Transl Psychiatry. 2018 Sep 4;8:178. doi: 10.1038/s41398-018-0236-1 (PMC6123450; doi:10.1038/s41398-018-0236-1)
Supplement: Supplementary file 16 — Supplementary Table 9 [file 41398_2018_236_MOESM16_ESM.docx]

Supplemental Table 9: Effect of lead SNPs on risk-taking in additional ethnicities

|  |  | white non-British (n=47982) | | | | | South Asian (n=6739) | | | | African-Caribbean (n=7095) | | | | Mixed (n=9452) | | | |
| --- | --- | --- | --- | --- | --- | --- | --- | --- | --- | --- | --- | --- | --- | --- | --- | --- | --- | --- |
| CHR | SNP | A1 | A2 | A1F | BETA | P | A1 | A1F | BETA | P | A1 | A1F | BETA | P | A1 | A1F | BETA | P |
| 1 | rs560977020 | C | T | 0.32 | -0.027 | 0.0716 | T | 0.48 | 0.024 | 0.5023 | T | 0.13 | 0.046 | 0.3793 | C | 0.50 | -0.039 | 0.2227 |
| 2 | rs2304681 | A | G | 0.37 | -0.018 | 0.2173 | A | 0.32 | 0.059 | 0.1208 | A | 0.32 | 0.019 | 0.5981 | A | 0.33 | -0.041 | 0.2046 |
| 3 | rs542809491 | A | T | 0.38 | 0.058 | **9.61E-05** | A | 0.41 | 0.025 | 0.5023 | T | 0.37 | 0.019 | 0.6110 | A | 0.35 | 0.027 | 0.4296 |
| 3 | rs9841382 | C | T | 0.15 | 0.025 | 0.1925 | C | 0.05 | 0.208 | **0.0087** | T | 0.44 | -0.060 | 0.1107 | C | 0.14 | 0.005 | 0.9216 |
| 6 | rs188973463 | G | T | 0.24 | -0.023 | 0.1678 | G | 0.24 | 0.027 | 0.5456 | G | 0.48 | 0.008 | 0.8396 | G | 0.30 | -0.035 | 0.3375 |
| 6 | rs566858049 | C | T | 0.38 | -0.013 | 0.3573 | C | 0.35 | 0.017 | 0.6552 | T | 0.27 | 0.022 | 0.5784 | C | 0.47 | -0.018 | 0.5646 |
| 7 | rs727644 | A | G | 0.41 | -0.045 | **0.0016** | A | 0.36 | -0.089 | **0.0222** | A | 0.17 | -0.021 | 0.6727 | A | 0.42 | -0.018 | 0.5864 |
| 8 | rs189335278 | A | T | 0.10 | -0.069 | **0.0029** | A | 0.04 | -0.018 | 0.8513 | A | 0.06 | -0.031 | 0.6542 | A | 0.06 | 0.007 | 0.9175 |
| 11 | rs10895735 | G | A | 0.24 | -0.001 | 0.9571 | G | 0.19 | 0.023 | 0.6157 | G | 0.25 | 0.021 | 0.6076 | G | 0.20 | 0.027 | 0.4721 |
| 15 | rs545973460 | A | G | 0.35 | -0.012 | 0.4090 | A | 0.20 | 0.031 | 0.4772 | A | 0.16 | 0.038 | 0.4204 | A | 0.20 | 0.018 | 0.6537 |
| 16 | rs145206681 | T | C | 0.06 | 0.004 | 0.9031 | T | 0.02 | -0.043 | 0.7218 | T | 0.03 | 0.056 | 0.5964 | T | 0.03 | -0.033 | 0.6955 |
