# Supplementary material for: Genetics of self-reported risk-taking behaviour, trans-ethnic consistency and relevance to brain gene expression
Source: Transl Psychiatry. 2018 Sep 4;8:178. doi: 10.1038/s41398-018-0236-1 (PMC6123450; doi:10.1038/s41398-018-0236-1)
Supplement: Supplementary file 17 — Supplementary Table 10 [file 41398_2018_236_MOESM17_ESM.docx]

Supplemental Table 10: Demographics of the MRI subset

|  | in MRI | |
| --- | --- | --- |
|  | controls | risk-takers^a^ |
| N | 6935 | 2700 |
| Men | 2956 (42.6) | 1640 (60.7) |
| Age (years) | 6746 (97.6) | 2590 (96.3) |
| BMI (kg/m2) | 55.4 (7.4) | 55.2 (7.7) |
| Current smoker | 26.5 (4.3) | 27.1 (4.2) |
| Ever smoker | 2273 (32.8) | 950 (35.3) |
| Age completed education^b^ | 2645 (38.2) | 1214 (45.1) |
| Has a degree | 17.1 (2.3) | 17.2 (2.4) |
| Townsend deprivation index | 2890 (42.3) | 1258 (47.4) |
| Unstable mood^c^ | -2.00 (2.60) | -1.82 (2.75) |
| Probable mood phenotyping^d^ | 2765 (40.4) | 1174 (44.2) |
| Comparison group^d^ | 3134 | 1207 |
| BD^d^ | 2300 (73.4) | 851 (70.5) |
| single episode depression^d^ | 19 (0.6) | 20 (1.7) |
| Moderate depression^d^ | 225 (7.0) | 95 (8.0) |
| Severe depression^d^ | 396 (12.6) | 159 (13.1) |
| any depression | 194 (6.0) | 83 (7.0) |
| Mental Health Questionnaire | 815 (26.0) | 336 (27.8) |
| BD | 5259 | 2008 |
| MDD | 58 (1.1) | 47 (2.4) |
| GAD | 1261 (28.3) | 511 (30.0) |
| any addiction | 354 (9.6) | 148 (10.6) |
| alcoholism | 243 (4.7) | 178 (9.0) |
| illicit drug addiction | 100 (1.9) | 64 (3.2) |
| OTC/prescription addiction | 15 (0.3) | 21 (1.1) |
| Ever cannabis | 37 (0.7) | 18 (0.9) |
| N | 961 (18.3) | 599 (29.9) |
| Where: ^a^ participants who answered "yes" to "do you consider yourself a risk taker?"; ^b^ based on a subset of 80 229 subjects; ^c^ Unstable mood, defined by the question ""Does your mood often go up and down?"; Paticipants who answered yes were classified as having unstable mood ; ^d^ definitions as per Smith et al, Plos One, 2013; BD, bipolar disorder; MDD, major depressive disorder; GAD, generalised anxiety disorder; OTC, over the counter. Addiction phenotypes based on self-report. | | |
