# Supplementary material for: Genetics of self-reported risk-taking behaviour, trans-ethnic consistency and relevance to brain gene expression
Source: Transl Psychiatry. 2018 Sep 4;8:178. doi: 10.1038/s41398-018-0236-1 (PMC6123450; doi:10.1038/s41398-018-0236-1)
Supplement: Supplementary file 23 — Supplementary Table 16 [file 41398_2018_236_MOESM23_ESM.docx]

Supplemental Table 16: Lead SNPs with replicated eQTLs in the dorsolateral prefrontal cortex

| SNP | Feature | Type | beta | dicovery pvalue | FDR p value | corrected Pvalue | replication pvalue | A allele | Coordinates | Symbol | Class | WhichTx |
| --- | --- | --- | --- | --- | --- | --- | --- | --- | --- | --- | --- | --- |
| rs2304681 | chr2:27322709-27324964(*) | J | -0.310 | 1.29E-25 | 3.36E-22 | 5.83E-18 | 3.99E-08 | - | chr2:27322709-27324964(*) | *CGREF1* | InEns | ["ENST00000402550"] |
|  | e119704 | E | -0.100 | 6.14E-23 | 7.04E-20 | 4.01E-15 | 2.11E-07 | - | chr2:27321757-27322708(-) |  | InEns | ["ENST00000440612","ENST00000402550"] |
|  | e119717 | E | -0.085 | 1.73E-15 | 9.85E-13 | 1.13E-07 | 1.94E-06 | - | chr2:27322221-27322708(-) |  | InEns | ["ENST00000452318"] |
|  | chr2:27323360-27324964(*) | J | 0.481 | 1.18E-14 | 1.11E-11 | 5.34E-07 | 3.17E-07 | + | chr2:27323360-27324964(*) |  | AltStartEnd | [] |
|  | chr2:27322709-27323283(*) | J | 0.391 | 1.61E-14 | 1.50E-11 | 7.33E-07 | 3.13E-04 | + | chr2:27322709-27323283(*) |  | InEns | ["ENST00000440612","ENST00000452318"] |
|  | chr2:27324757-27324964(*) | J | 0.157 | 1.13E-13 | 9.51E-11 | 5.12E-06 | 5.96E-10 | + | chr2:27324757-27324964(*) |  | InEns | ["ENST00000452318","ENST00000402394","ENST00000405600","ENST00000312734","ENST00000404694","ENST00000260595"] |
|  | e119715 | E | 0.116 | 5.62E-12 | 2.08E-09 | 3.67E-04 | 6.34E-05 | + | chr2:27324656-27324756(-) |  | InEns | ["ENST00000452318"] |
|  | e119711 | E | 0.102 | 9.20E-10 | 2.45E-07 | 0.0602 | 0.0181 | + | chr2:27333541-27333970(-) |  | InEns | ["ENST00000452318"] |
|  | e119731 | E | 0.118 | 2.23E-09 | 5.55E-07 | 0.1455 | 0.0133 | + | chr2:27333541-27333836(-) |  | InEns | ["ENST00000312734"] |
|  | e119747 | E | 0.081 | 3.39E-08 | 6.82E-06 | 1.0000 | 0.0016 | + | chr2:27324364-27324756(-) |  | InEns | ["ENST00000260595"] |
|  | chr2:27322709-27324882(*) | J | -0.344 | 1.51E-06 | 3.87E-04 | 1.0000 | 0.0263 | - | chr2:27322709-27324882(*) |  | ExonSkip | [] |
|  | TCONS_00390576 | T | -0.230 | 1.59E-06 | 7.79E-04 | 1.0000 | 0.0000 | - | chr2:27321757-27341983(-) |  | InEns | ["ENST00000402550"] |
|  | chr2:27323360-27324882(*) | J | 0.323 | 1.64E-06 | 4.17E-04 | 1.0000 | 0.0089 | + | chr2:27323360-27324882(*) |  | AltStartEnd | [] |
|  | TCONS_00390569 | T | 0.075 | 9.99E-06 | 3.87E-03 | 1.0000 | 0.0046 | + | chr2:27320515-27342024(-) |  | ExonSkip | ["ENST00000312734"] |
|  | chr2:27323411-27324964(*) | J | -0.274 | 1.64E-05 | 3.15E-03 | 1.0000 | 0.0500 | - | chr2:27323411-27324964(*) |  | ExonSkip | [] |
|  | e119741 | E | 0.054 | 1.94E-05 | 2.01E-03 | 1.0000 | 0.0018 | + | chr2:27324094-27324756(-) |  | InEns | ["ENST00000404694"] |
|  | chr2:27323411-27324882(*) | J | -0.239 | 3.1E-05 | 5.42E-03 | 1.0000 | 4.81E-04 | - | chr2:27323411-27324882(*) |  | InEns | ["ENST00000440612"] |
|  | chr2:27310220-27319596(*) | J | 0.284 | 3.24E-21 | 5.88E-18 | 1.47E-13 | 5.34E-08 | + | chr2:27310220-27319596(*) | *KHK* | ExonSkip | [] |
|  | e119698 | E | -0.082 | 3.86E-11 | 1.27E-08 | 0.0025 | 1.14E-04 | - | chr2:27322533-27322837(+) |  | InEns | ["ENST00000464371"] |
|  | e119681 | E | -0.082 | 1.73E-10 | 5.17E-08 | 0.0113 | 3.52E-05 | - | chr2:27322533-27322769(+) |  | InEns | ["ENST00000490823"] |
|  | er40926 | ER | 0.098 | 2.03E-10 | 5.80E-08 | 0.0079 | 0.2264 | + | chr2:27319570-27319669(*) |  | AltStartEnd | [] |
|  | e119663 | E | -0.064 | 2.12E-10 | 6.23E-08 | 0.0138 | 0.0188 | - | chr2:27322533-27323619(+) |  | InEns | ["ENST00000260599"] |
|  | e119671 | E | -0.062 | 5.07E-10 | 1.41E-07 | 0.0332 | 0.0211 | - | chr2:27322533-27323640(+) |  | InEns | ["ENST00000260598"] |
|  | chr2:27319670-27320370(*) | J | 0.094 | 2.29E-07 | 7.10E-05 | 1.00 | 0.0275 | + | chr2:27319670-27320370(*) |  | InEns | ["ENST00000260599","ENST00000260598","ENST00000490823","ENST00000429697","ENST00000469936","ENST00000464371"] |
|  | e119657 | E | -0.091 | 3.09E-07 | 5.10E-05 | 1.00 | 0.0324 | - | chr2:27315200-27315316(+) |  | InEns | ["ENST00000260599","ENST00000260598","ENST00000490823","ENST00000429697"] |
|  | chr2:27310220-27315199(*) | J | -0.092 | 1.04E-05 | 2.12E-03 | 1.00 | 0.0022 | - | chr2:27310220-27315199(*) |  | InEns | ["ENST00000260599","ENST00000260598","ENST00000429697"] |
|  | e119357 | E | -0.123 | 2.50E-07 | 4.22E-05 | 1.00 | 6.41E-04 | - | chr2:27151123-27151191(+) | *DPYSL5* | InEns | ["ENST00000288699","ENST00000401478"] |
|  | e119358 | E | -0.119 | 2.88E-07 | 4.78E-05 | 1.00 | 0.0088 | - | chr2:27154508-27154552(+) |  | InEns | ["ENST00000288699","ENST00000401478"] |
|  | er40806 | ER | -0.120 | 7.33E-07 | 1.10E-04 | 1.00 | 0.0087 | - | chr2:27154508-27154552(*) |  | InEns | [] |
|  | chr2:27151192-27154507(*) | J | -0.122 | 7.61E-07 | 2.10E-04 | 1.00 | 0.0389 | - | chr2:27151192-27154507(*) |  | InEns | ["ENST00000288699","ENST00000401478"] |
|  | er40805 | ER | -0.119 | 1.17E-06 | 1.68E-04 | 1.00 | 0.0034 | - | chr2:27151123-27151191(*) |  | InEns | [] |
|  | e119387 | E | -0.075 | 1.53E-06 | 2.15E-04 | 1.00 | 0.0387 | - | chr2:27161327-27163040(+) |  | InEns | ["ENST00000484882"] |
|  | e119359 | E | -0.112 | 2.87E-06 | 3.77E-04 | 1.00 | 0.0191 | - | chr2:27156126-27156201(+) |  | InEns | ["ENST00000288699","ENST00000401478"] |
|  | chr2:27154553-27156125(*) | J | -0.114 | 5.15E-06 | 0.0011 | 1.00 | 0.0161 | - | chr2:27154553-27156125(*) |  | InEns | ["ENST00000288699","ENST00000401478"] |
|  | e119361 | E | -0.093 | 1.26E-05 | 0.0014 | 1.00 | 0.0067 | - | chr2:27162899-27163040(+) |  | InEns | ["ENST00000288699","ENST00000401478"] |
|  | e119356 | E | -0.098 | 1.34E-05 | 0.0015 | 1.00 | 0.0019 | - | chr2:27150121-27150300(+) |  | InEns | ["ENST00000288699","ENST00000401478"] |
|  | e119360 | E | -0.089 | 1.57E-05 | 0.0017 | 1.00 | 0.0014 | - | chr2:27157446-27157602(+) |  | InEns | ["ENST00000288699","ENST00000401478"] |
|  | er40812 | ER | -0.094 | 1.77E-05 | 0.0019 | 1.00 | 0.0023 | - | chr2:27157446-27158250(*) |  | AltStartEnd | [] |
|  | e119364 | E | -0.089 | 2.67E-05 | 0.0027 | 1.00 | 0.0341 | - | chr2:27167524-27167692(+) |  | InEns | ["ENST00000288699","ENST00000401478","ENST00000484882"] |
|  | e119382 | E | -0.098 | 3.75E-05 | 0.0036 | 1.00 | 0.0018 | - | chr2:27150121-27150269(+) |  | InEns | ["ENST00000431402"] |
|  | er40804 | ER | -0.106 | 4.22E-05 | 0.0040 | 1.00 | 0.0018 | - | chr2:27150121-27150300(*) |  | InEns | [] |
|  | er40837 | ER | -0.096 | 4.29E-05 | 0.0040 | 1.00 | 0.0170 | - | chr2:27167524-27167692(*) |  | InEns | [] |
|  | e119386 | E | -0.098 | 7.56E-05 | 0.0065 | 1.00 | 0.0036 | - | chr2:27150121-27150224(+) |  | InEns | ["ENST00000434719"] |
|  | er40811 | ER | -0.098 | 7.96E-05 | 0.0068 | 1.00 | 0.0051 | - | chr2:27156126-27156201(*) |  | InEns | [] |
|  | chr2:27355792-27355993(*) | J | 0.377 | 2.74E-09 | 1.24E-06 | 0.1244 | 0.0370 | + | chr2:27355792-27355993(*) | *PREB^a^* | AltStartEnd | [] |
|  | er40840 | ER | -0.195 | 2.42E-05 | 0.0025 | 1.00 | 0.0100 | - | chr2:27173383-27173848(*) | *PREB^b^* | AltStartEnd | [] |
| rs17187323 | chr15:74044204-74044450(*) | J | -1.156 | 9.13E-14 | 7.79E-11 | 4.14E-06 | 1.02E-12 | - | chr15:74044204-74044450(*) | *C15orf59* | AltStartEnd | [] |
|  | chr15:74032818-74032908(*) | J | -0.200 | 8.70E-10 | 4.29E-07 | 0.0395 | 1.05E-06 | - | chr15:74032818-74032908(*) |  | InEns | ["ENST00000558834"] |
|  | chr15:74032814-74032908(*) | J | -0.241 | 2.60E-09 | 1.18E-06 | 0.1182 | 1.11E-05 | - | chr15:74032814-74032908(*) |  | AltStartEnd | [] |
|  | TCONS_00236165 | T | -0.168 | 1.68E-07 | 1.05E-04 | 1.00 | 1.02E-07 | - | chr15:74022182-74046408(-) |  | ExonSkip | ["ENST00000569673"] |
|  | TCONS_00236163 | T | -0.080 | 5.64E-07 | 3.11E-04 | 1.00 | 1.68E-06 | - | chr15:74022182-74046408(-) |  | ExonSkip | ["ENST00000569673"] |
|  | er282018 | ER | -0.066 | 1.18E-05 | 0.0013 | 1.00 | 2.40E-07 | - | chr15:74043316-74044203(*) |  | AltStartEnd | [] |
|  | er282016 | ER | 0.095 | 1.49E-05 | 0.0016 | 1.00 | 1.37E-02 | + | chr15:74027794-74032131(*) |  | Novel | [] |
|  | ENSG00000205363 | G | -0.046 | 3.14E-05 | 0.0022 | 1.00 | 3.26E-09 | - | chr15:74032141-74045088(-) |  | InEns | ["ENST00000569673","ENST00000379822","ENST00000558834","ENST00000559817"] |
|  | e883071 | Exon | -0.058 | 5.42E-05 | 0.0049 | 1.00 | 3.20E-05 | - | chr15:74043316-74043816(-) |  | InEns | ["ENST00000379822"] |
|  | chr15:74032984-74043315(*) | J | -0.063 | 5.92E-05 | 0.0094 | 1.00 | 1.73E-04 | - | chr15:74032984-74043315(*) |  | InEns | ["ENST00000569673","ENST00000379822","ENST00000559817"] |
|  | e883069 | E | -0.055 | 6.51E-05 | 0.0057 | 1.00 | 4.04E-07 | - | chr15:74043316-74044038(-) |  | InEns | ["ENST00000569673"] |
| rs3943093 | e109958 | E | 0.109 | 9.07E-07 | 1.35E-04 | 1.00 | 2.88E-04 | + | chr1:243468314-243468389(+) | *SDCCAG8* | InEns | ["ENST00000482234"] |
|  | e109976 | E | 0.059 | 3.03E-06 | 3.95E-04 | 1.00 | 0.0395 | + | chr1:243468015-243468859(+) |  | InEns | ["ENST00000496361"] |
|  | e109963 | E | 0.057 | 2.71E-05 | 0.0027 | 1.00 | 0.0265 | + | chr1:243468314-243469850(+) |  | InEns | ["ENST00000476722"] |
| Where: J, Junction; E, Exon; T, Transcript; G, gene; *CGREF1*, ENSG00000138028; *KHK*, ENSG00000138030; *DPYSL5*, ENSG00000157851; *PREB*^a^, ENSG00000138073; *PREB*^b^, ENSG00000230286; *C15orf59*, ENSG00000205363; *SDCCAG8*, ENSG00000054282. | | | | | | | | | | | | |
