# Supplementary material for: Genetics of self-reported risk-taking behaviour, trans-ethnic consistency and relevance to brain gene expression
Source: Transl Psychiatry. 2018 Sep 4;8:178. doi: 10.1038/s41398-018-0236-1 (PMC6123450; doi:10.1038/s41398-018-0236-1)
Supplement: Supplementary file 24 — Supplementary Table 17 [file 41398_2018_236_MOESM24_ESM.docx]

Supplemental Table 17: Predicted effects of SNPs with suggestive evidence of association with risk-taking

| SNP | CHR | BP | A1 | Gene | Feature | aa | SIFT | PolyPhen | PUBMED |
| --- | --- | --- | --- | --- | --- | --- | --- | --- | --- |
| rs11542623 | 1 | 46280492 | G | *LRRC41* | NM_006369.4 | V/L | deleterious(0.01) | benign(0.151) | - |
| rs11542623 | 1 | 46280492 | T | *LRRC41* | NM_006369.4 | V/I | deleterious(0.03) | benign(0.007) | - |
| rs9865 | 1 | 46361784 | G | *NSUN4* | NM_001256127.1 | I/V | tolerated(0.27) | benign(0.003) | - |
| rs1124649 | 2 | 27037601 | A | *TMEM214* | NM_001083590.1 | V/M | deleterious(0.01) | probably_damaging(0.995) | 16175505, 22568750 |
| rs2304681 | 2 | 27092384 | A | *KHK* | NM_000221.2 | V/I | deleterious(0.04) | benign(0.01) | - |
| rs2384572 | 2 | 27101883 | C | *CGREF1* | NM_001166239.1 | I/M | deleterious(0) | probably_damaging(0.951) | 22581228 |
| rs202239154 | 2 | 27130719 | C | *ABHD1* | NM_032604.3 | L/S | tolerated_low_confidence(0.92) | benign(0) | - |
| rs148409741 | 2 | 27132035 | A | *PREB* | NM_013388.4 | A/V | deleterious(0) | probably_damaging(0.996) | - |
| rs940389 | 2 | 48581013 | C | *STON1-GTF2A1L* | NM_001198593.1 | R/T | tolerated(0.11) | benign(0.037) | 20885795 |
| rs200272831 | 6 | 26156756 | C | *HIST1H1E* | NM_005321.2 | K/N | deleterious(0.01) | benign(0.42) | - |
| rs16897515 | 6 | 27310241 | A | *POM121L2* | NM_033482.3 | G/C | deleterious(0.03) | probably_damaging(0.982) | 25380769, 23894743 |
| rs575015627 | 6 | 27311933 | A | *POM121L2* | NM_033482.3 | P/S | deleterious(0.03) | probably_damaging(0.992) | - |
| rs200484 | 6 | 27807896 | G | *HIST1H2BL* | NM_003519.3 | L/P | tolerated_low_confidence(1) | benign(0) | 25505091 |
| rs539861690^a^ | 6 | 28251581 | A | *ZKSCAN4* | NM_019110.4 | Q/* | - | - | - |
| rs147753198 | 6 | 28287150 | A | *PGBD1* | NM_001184743.1 | F/L | tolerated_low_confidence(0.78) | benign(0) | - |
| rs115701378 | 6 | 28301802 | G | *PGBD1* | NM_001184743.1 | I/V | tolerated(0.42) | benign(0.193) | - |
| rs1997660 | 6 | 28301886 | G | *PGBD1* | NM_001184743.1 | I/V | tolerated(1) | benign(0) | 23437227, 24282510 |
| rs853684 | 6 | 28326773 | C | *ZSCAN31* | NM_001135215.1 | K/R | tolerated(1) | benign(0) | - |
| rs853678 | 6 | 28329536 | A | *ZSCAN31* | NM_001135215.1 | T/S | tolerated(0.24) | benign(0.021) | 25505091 |
| rs853678 | 6 | 28329536 | C | *ZSCAN31* | NM_001135215.1 | T/A | deleterious(0.02) | benign(0.023) | 25505091 |
| rs13201752 | 6 | 28363350 | G | *ZKSCAN3* | NM_001242894.1 | K/E | tolerated(0.8) | benign(0.014) | - |
| rs13201753 | 6 | 28363351 | C | *ZKSCAN3* | NM_001242894.1 | K/T | tolerated(0.45) | benign(0) | - |
| rs1361385 | 6 | 28390543 | G | *ZSCAN12* | NM_001163391.1 | C/R | deleterious(0) | probably_damaging(0.985) | 22245343 |
| rs1416918 | 6 | 28390558 | A | *ZSCAN12* | NM_001163391.1 | R/C | deleterious(0.01) | benign(0) | - |
| rs1416918 | 6 | 28390558 | C | *ZSCAN12* | NM_001163391.1 | R/G | tolerated(0.21) | benign(0) | - |
| rs536999453 | 6 | 28391049 | C | *ZSCAN12* | NM_001163391.1 | Y/C | deleterious(0.02) | benign(0.293) | - |
| rs3116855 | 6 | 29173855 | C | *OR2J2* | NM_030905.2 | Y/H | deleterious(0) | probably_damaging(0.985) | - |
| rs3116855 | 6 | 29173888 | T | *OR2J2* | NM_030905.2 | H/Y | tolerated(0.78) | benign(0) | - |
| rs3116856 | 6 | 29174105 | T | *OR2J2* | NM_030905.2 | A/V | tolerated(0.25) | possibly_damaging(0.841) | - |
| rs3116856 | 6 | 29174072 | C | *OR2J2* | NM_030905.2 | V/A | tolerated(1) | benign(0) | - |
| rs189160951 | 6 | 29355889 | G | *OR5V1* | NM_030876.5 | A/P | deleterious(0) | possibly_damaging(0.786) | - |
| rs529382624 | 6 | 29397508 | G | *OR12D2* | NM_013936.3 | D/G | deleterious(0.03) | benign(0.35) | - |
| rs35951447 | 8 | 142281909 | C | *TSNARE1* | XM_011516922.1 | K/E | - | - | - |
| rs62519835 | 8 | 64580975 | A | *BHLHE22* | NM_152414.4 | L/Q | deleterious_low_confidence(0) | benign(0.127) | - |
| rs73145643 | 12 | 100739970 | C | *ANO4* | XM_011537911.2 | I/L | - | - | - |
| rs7177192 | 15 | 40606445 | C | *CASC5* | NM_144508.4 | R/T | tolerated(0.65) | benign(0) | 18414213 |
| rs12911738 | 15 | 40611486 | G | *CASC5* | NM_144508.4 | T/A | tolerated(1) | benign(0) | 18414213 |
| rs12440118 | 15 | 42451896 | G | *ZNF106* | NM_022473.2 | W/R | tolerated(0.94) | benign(0) | 28106113 |
| Where: all variants missense with moderate impact, except ^a^, which is a stop-gained variant with high impact | | | | | | | | | |
