# Supplementary material for: Genetics of self-reported risk-taking behaviour, trans-ethnic consistency and relevance to brain gene expression
Source: Transl Psychiatry. 2018 Sep 4;8:178. doi: 10.1038/s41398-018-0236-1 (PMC6123450; doi:10.1038/s41398-018-0236-1)
Supplement: Supplementary file 25 — Supplementary Table 18 [file 41398_2018_236_MOESM25_ESM.docx]

Supplemental Table 18: Effects of SNPs within 500kb of the lead risk-taking SNPs on relevant psychiatric and cardiometabolic traits

| GWAS catalogue | | | | | | | UKBiobank risk-taking GWAS | | | | |
| --- | --- | --- | --- | --- | --- | --- | --- | --- | --- | --- | --- |
| REGION | PUBMEDID | DISEASE/TRAIT | CHR | SNP-RA | RAF | OR or BETA | RA | RAF | BETA | SE | P |
| 1q43 | 20421936 | Obesity (early onset extreme) | 1 | rs12145833-T | NR | 1.19 |  |  |  |  |  |
| 1q43 | 27225129 | Educational attainment (years of education) | 1 | rs2992632-A | 0.72 | 0.02 | T | 0.29 | -0.03 | 0.01 | 4.70E-06 |
| 1q43 | 23974872 | Schizophrenia | 1 | rs1538774-G | 0.74 | 1.09 |  |  |  |  |  |
| 1q43 | 25056061 | Schizophrenia | 1 | rs77149735-A | 0.02 | 1.32 |  |  |  |  |  |
| 1q43 | 26198764 | Schizophrenia | 1 | rs77149735-A | NR | 1.32 |  |  |  |  |  |
| 1q43 | 28991256 | Schizophrenia | 1 | rs10803138-G | NR | 1.07 |  |  |  |  |  |
| 1q43 | 21926974 | Schizophrenia | 1 | rs6703335-? | NR | 1.09 |  |  |  |  |  |
| 1q43 | 24166486 | Schizophrenia, schizoaffective disorder or bipolar disorder | 1 | rs6703335-? | 0.56 |  |  |  |  |  |  |
| 1q44 | 23974872 | Schizophrenia | 1 | rs14403-C | 0.77 | 1.10 |  |  |  |  |  |
| 1q44 | 26198764 | Schizophrenia | 1 | rs13376709-C | NR | 1.06 | T | 0.31 | -0.03 | 0.01 | 7.75E-08 |
| 1q44 | 28991256 | Schizophrenia | 1 | rs35978510-C | NR | 1.06 | T | 0.32 | -0.03 | 0.01 | 1.09E-07 |
| 1q44 | 20031603 | RR interval (heart rate) | 1 | rs4132509-A | 0.21 | 0.18 |  |  |  |  |  |
| 1q44 | 23726511 | Post-traumatic stress disorder (asjusted for relatedness) | 1 | rs4430311-? | NR |  | C | 0.33 | -0.03 | 0.01 | 2.62E-07 |
| 1q44 | 23400010 | Thiazide-induced adverse metabolic effects in hypertensive patients | 1 | rs12127679-T | 0.19 | 22.31 |  |  |  |  |  |
| 1q44 | 21441570 | Diabetic retinopathy | 1 | rs10927101-A | 0.38 | 1.33 |  |  |  |  |  |
| 1q44 | 21441570 | Diabetic retinopathy | 1 | rs476141-A | 0.51 | 1.37 |  |  |  |  |  |
| 1q44 | 22885689 | Schizophrenia | 1 | rs10429924-? | NR | 1.61 |  |  |  |  |  |
| 1q44 | 29071344 | Major depression and alcohol dependence | 1 | rs488109-G | 0.40 | 0.48 |  |  |  |  |  |
| 2p23.3 | 23251661 | Obesity-related traits | 2 | rs11887277-A | 0.46 | 0.04 |  |  |  |  |  |
| 2p23.3 | 22581228 | Fasting glucose-related traits (BMI interaction) | 2 | rs1371614-? |  |  |  |  |  |  |  |
| 2p23.3 | 23726366 | Triglycerides | 2 | rs4665972-T | 0.12 | 0.07 |  |  |  |  |  |
| 2p23.3 | 18454146 | Waist circumference and related phenotypes | 2 | rs1260326-? | NR |  |  |  |  |  |  |
| 2p23.3 | 19060906 | Triglycerides | 2 | rs1260326-T | 0.45 | 0.12 |  |  |  |  |  |
| 2p23.3 | 19936222 | Lipid metabolism phenotypes | 2 | rs1260326-? | NR | 0.05 |  |  |  |  |  |
| 2p23.3 | 19936222 | Lipid metabolism phenotypes | 2 | rs1260326-? | NR | 0.36 |  |  |  |  |  |
| 2p23.3 | 20081857 | Two-hour glucose challenge | 2 | rs1260326-T | NR | 0.07 |  |  |  |  |  |
| 2p23.3 | 20139978 | Triglycerides | 2 | rs1260326-C | 0.45 | 0.10 |  |  |  |  |  |
| 2p23.3 | 20657596 | Hypertriglyceridemia | 2 | rs1260326-T | 0.41 | 1.75 |  |  |  |  |  |
| 2p23.3 | 20686565 | Triglycerides | 2 | rs1260326-T | 0.41 | 8.76 |  |  |  |  |  |
| 2p23.3 | 20686565 | Cholesterol, total | 2 | rs1260326-T | 0.41 | 1.91 |  |  |  |  |  |
| 2p23.3 | 21300955 | C-reactive protein levels | 2 | rs1260326-T | NR | 0.07 |  |  |  |  |  |
| 2p23.3 | 21943158 | Cardiovascular disease risk factors | 2 | rs1260326-T | 0.40 | 0.08 |  |  |  |  |  |
| 2p23.3 | 22001757 | Liver enzyme levels (gamma-glutamyl transferase) | 2 | rs1260326-T | 0.38 | 3.20 |  |  |  |  |  |
| 2p23.3 | 23118302 | Lipoprotein-associated phospholipase A2 activity and mass | 2 | rs1260326-T | 0.44 | 0.01 |  |  |  |  |  |
| 2p23.3 | 23505323 | Hypertriglyceridemia | 2 | rs1260326-? | 0.26 | 1.41 |  |  |  |  |  |
| 2p23.3 | 23903356 | Glycemic traits (pregnancy) | 2 | rs1260326-T | 0.41 | 0.01 |  |  |  |  |  |
| 2p23.3 | 24097068 | Cholesterol, total | 2 | rs1260326-T | 0.39 | 0.05 |  |  |  |  |  |
| 2p23.3 | 24097068 | Triglycerides | 2 | rs1260326-T | 0.39 | 0.12 |  |  |  |  |  |
| 2p23.3 | 24386095 | Lipid traits | 2 | rs1260326-T | 0.44 | 0.07 |  |  |  |  |  |
| 2p23.3 | 25961943 | Cholesterol, total | 2 | rs1260326-T | 0.36 | 0.05 |  |  |  |  |  |
| 2p23.3 | 25961943 | Triglycerides | 2 | rs1260326-T | 0.36 | 0.12 |  |  |  |  |  |
| 2p23.3 | 26831199 | Glomerular filtration rate in non diabetics (creatinine) | 2 | rs1260326-T | NR | 0.01 |  |  |  |  |  |
| 2p23.3 | 27588450 | Glomerular filtration rate | 2 | rs1260326-C | 0.60 | 0.83 |  |  |  |  |  |
| 2p23.3 | 27798624 | Resting heart rate | 2 | rs1260326-C | 0.39 | 0.27 |  |  |  |  |  |
| 2p23.3 | 28334899 | Triglyceride levels | 2 | rs1260326-T | 0.41 | 0.12 |  |  |  |  |  |
| 2p23.3 | 28937693 | Alcohol consumption | 2 | rs1260326-T | 0.39 | 0.03 |  |  |  |  |  |
| 2p23.3 | 28937693 | Alcohol consumption in current drinkers | 2 | rs1260326-G | 0.38 | 0.03 |  |  |  |  |  |
| 2p23.3 | 28270201 | Fasting plasma glucose | 2 | rs780095-G | 0.43 | 0.07 |  |  |  |  |  |
| 2p23.3 | 17463246 | Triglycerides | 2 | rs780094-T | 0.35 | 0.50 |  |  |  |  |  |
| 2p23.3 | 18179892 | LDL cholesterol | 2 | rs780094-T | 0.39 |  |  |  |  |  |  |
| 2p23.3 | 18193043 | Triglycerides | 2 | rs780094-T | 0.39 | 8.59 |  |  |  |  |  |
| 2p23.3 | 18193044 | Triglycerides | 2 | rs780094-T | 0.34 | 0.13 |  |  |  |  |  |
| 2p23.3 | 18439548 | C-reactive protein | 2 | rs780094-A | NR | 0.14 |  |  |  |  |  |
| 2p23.3 | 19060911 | Triglycerides | 2 | rs780094-G | 0.63 | 0.10 |  |  |  |  |  |
| 2p23.3 | 20081858 | Fasting glucose-related traits | 2 | rs780094-C | 0.62 |  |  |  |  |  |  |
| 2p23.3 | 22399527 | Metabolic syndrome | 2 | rs780094-A | 0.36 | 0.13 |  |  |  |  |  |
| 2p23.3 | 22581228 | Fasting insulin-related traits (BMI interaction) | 2 | rs780094-? | NR |  |  |  |  |  |  |
| 2p23.3 | 22885922 | Type 2 diabetes | 2 | rs780094-C | 0.61 | 1.06 |  |  |  |  |  |
| 2p23.3 | 23726366 | Triglycerides | 2 | rs780094-C | 0.36 | 0.07 |  |  |  |  |  |
| 2p23.3 | 25187374 | Fasting plasma glucose | 2 | rs780094-? | NR | 0.05 |  |  |  |  |  |
| 2p23.3 | 27599772 | Hypertriglyceridemia | 2 | rs780094-T | NR | 1.22 |  |  |  |  |  |
| 2p23.3 | 27911795 | Alcohol consumption | 2 | rs780094-T | 0.40 | 0.01 |  |  |  |  |  |
| 2p23.3 | 28334899 | Triglyceride levels | 2 | rs780094-T | 0.52 | 0.10 |  |  |  |  |  |
| 2p23.3 | 28485404 | Alcohol consumption (drinks per week) | 2 | rs780094-T | 0.41 | 0.03 |  |  |  |  |  |
| 2p23.3 | 28869590 | Type 2 diabetes | 2 | rs780094-C | 0.59 | 0.06 |  |  |  |  |  |
| 2p23.3 | 21386085 | Triglycerides-Blood Pressure (TG-BP) | 2 | rs780093-A | NR | 0.18 |  |  |  |  |  |
| 2p23.3 | 21386085 | Waist Circumference - Triglycerides (WC-TG) | 2 | rs780093-A | NR | 0.19 |  |  |  |  |  |
| 2p23.3 | 26780889 | Triglycerides | 2 | rs780093-T | 0.36 | 0.11 |  |  |  |  |  |
| 2p23.3 | 26818947 | Type 2 diabetes | 2 | rs780093-C | NR | 1.08 |  |  |  |  |  |
| 2p23.3 | 26833098 | circulating leptin levels | 2 | rs780093-C | 0.61 | 0.03 |  |  |  |  |  |
| 2p23.3 | 26833098 | circulating leptin levels adjusted for BMI | 2 | rs780093-C | 0.61 | 0.03 |  |  |  |  |  |
| 2p23.3 | 28334899 | Total cholesterol levels | 2 | rs780093-T | 0.41 | 0.05 |  |  |  |  |  |
| 2p23.3 | 21909109 | Triglycerides | 2 | rs780092-G | 0.33 | 0.05 |  |  |  |  |  |
| 2p23.3 | 24023260 | Lipid traits | 2 | rs780092-A | 0.68 | 0.08 |  |  |  |  |  |
| 2p23.3 | 28334899 | Total cholesterol levels | 2 | rs780092-A | 0.67 | 0.06 |  |  |  |  |  |
| 2p23.3 | 28334899 | Triglyceride levels | 2 | rs814295-A | 0.67 | 0.10 |  |  |  |  |  |
| 2p23.3 | 20864672 | Triglycerides | 2 | rs1260333-C | 0.55 | 0.05 |  |  |  |  |  |
| 2p23.3 | 26582766 | Triglycerides | 2 | rs1260333-A | 0.52 | 0.02 |  |  |  |  |  |
| 2p23.3 | 26582766 | Cholesterol, total | 2 | rs1260333-A | 0.52 | 1.77 |  |  |  |  |  |
| 2p23.3 | 28937693 | Alcohol consumption | 2 | rs11127048-G | 0.38 | 0.04 |  |  |  |  |  |
| 2p23.3 | 28485404 | Alcohol consumption (drinks per week) | 2 | rs4665985-C | 0.28 | 0.04 |  |  |  |  |  |
| 2p23.3 | 21386085 | Waist Circumference - Triglycerides (WC-TG) | 2 | rs1919128-A | NR | 0.18 |  |  |  |  |  |
| 2p23.3 | 21386085 | Waist Circumference - Triglycerides (WC-TG) | 2 | rs13022873-A | NR | 0.17 |  |  |  |  |  |
| 2p23.3 | 22359512 | Phospholipid levels (plasma) | 2 | rs4666002-? | NR | 0.00 |  |  |  |  |  |
| 2p23.3 | 21386085 | Waist Circumference - Triglycerides (WC-TG) | 2 | rs3749147-C | NR | 0.18 |  |  |  |  |  |
| 2p23.3 | 22159054 | Alzheimer's disease | 2 | rs17006206-G | 0.10 | 2.05 |  |  |  |  |  |
| 2p23.2 | 22581228 | Fasting glucose-related traits (BMI interaction) | 2 | rs3736594-? |  |  |  |  |  |  |  |
| 2p23.2 | 28991256 | Schizophrenia | 2 | rs12623170-C | NR | 1.06 |  |  |  |  |  |
| 2p23.2 | 26198764 | Schizophrenia | 2 | rs12474906-A | NR | 1.07 |  |  |  |  |  |
| 2p23.3 | 27599772 | Hypertriglyceridemia | 2 | rs780093-?; rs780094-? | NR | 1.35 |  |  |  |  |  |
| 3p12.1 | 28937693 | Alcohol consumption | 3 | rs1376935-A | 0.32 | 0.03 |  |  |  |  |  |
| 3p12.1 | 28937693 | Alcohol consumption in current drinkers | 3 | rs13078384-A | 0.31 | 0.02 |  |  |  |  |  |
| 3p12.1 | 28937693 | Alcohol consumption | 3 | rs67028245-A | 0.40 | 0.02 |  |  |  |  |  |
| 3p12.1 | 22589738 | Visceral fat | 3 | rs13323436-A | 0.10 |  | A | 0.08 | 0.05 | 0.01 | 1.04E-06 |
| 3p12.1 | 28937693 | Alcohol consumption | 3 | rs9841829-G | 0.23 | 0.02 | G | 0.23 | 0.05 | 0.01 | **3.94E-13** |
| 3p12.1 | 25644384 | Cognitive function | 3 | rs17518584-? | NR | 0.03 | C | 0.37 | 0.05 | 0.01 | **1.11E-17** |
| 3p12.1 | 25869804 | Information processing speed | 3 | rs17518584-T | 0.64 | 5.92 | C | 0.37 | 0.05 | 0.01 | **1.11E-17** |
| 3p12.1 | 27046643 | Educational attainment | 3 | rs55686445-? | NR | 0.03 | C | 0.35 | -0.04 | 0.01 | **7.32E-13** |
| 3p12.1 | 27225129 | Educational attainment (years of education) | 3 | rs62263923-A | 0.64 | 0.02 | G | 0.35 | -0.04 | 0.01 | **1.43E-12** |
| 3p12.1 | 25673413 | Body mass index | 3 | rs13078960-G | 0.20 | 0.03 |  |  |  |  |  |
| 3p12.1 | 28443625 | BMI (adjusted for smoking behaviour) | 3 | rs13078960-T | 0.81 | 0.03 |  |  |  |  |  |
| 3p12.1 | 28443625 | Body mass index (joint analysis main effects and smoking interaction) | 3 | rs13078960-T | 0.81 |  |  |  |  |  |  |
| 3p12.1 | 28443625 | BMI in non-smokers | 3 | rs13078960-T | 0.81 | 0.02 |  |  |  |  |  |
| 3p12.1 | 25673412 | Waist circumference | 3 | rs2325036-A | 0.60 | 0.02 | C | 0.38 | -0.03 | 0.01 | 8.34E-07 |
| 3p12.1 | 25673412 | Hip circumference | 3 | rs13098327-A | 0.20 | 0.03 |  |  |  |  |  |
| 3p12.1 | 28448500 | Body mass index (joint analysis main effects and physical activity interaction) | 3 | rs9852127-? | NR |  |  |  |  |  |  |
| 3p12.1 | 28448500 | Body mass index | 3 | rs9852127-A | 0.19 | 0.03 |  |  |  |  |  |
| 3p12.1 | 28448500 | Body mass index | 3 | rs9852859-C | 0.19 | 0.03 |  |  |  |  |  |
| 3p12.1 | 28448500 | Body mass index in physically active individuals | 3 | rs9818122-C | 0.20 | 0.03 |  |  |  |  |  |
| 3p12.1 | 22832960 | Temperament | 3 | rs12494658-T | 0.75 | 0.08 |  |  |  |  |  |
| 3p12.1 | 20935630 | Body mass index | 3 | rs13078807-G | 0.20 | 0.10 |  |  |  |  |  |
| 3p12.1 | 23563607 | Obesity | 3 | rs13078807-G | 0.20 | 1.06 |  |  |  |  |  |
| 3p12.1 | 28892062 | Body mass index | 3 | rs12495178-C | 0.37 | 0.01 | C | 0.35 | -0.03 | 0.01 | 2.77E-06 |
| 3p12.1 | 25778476 | Alzheimer's disease in APOE e4+ carriers | 3 | rs71316816-C | 0.92 | 1.25 |  |  |  |  |  |
| 3p12.1 | 27046643 | Educational attainment | 3 | rs112374913-? | NR | 0.03 |  |  |  |  |  |
| 3p12.1 | 22589738 | Subcutaneous adipose tissue | 3 | rs2324999-T | 0.20 |  |  |  |  |  |  |
| 3p12.1 | 22589738 | Subcutaneous adipose tissue | 3 | rs2324999-T | 0.20 |  |  |  |  |  |  |
| 3q26.33 | 26433762 | Bipolar disorder and eating disorder | 3 | rs1805576-G | 0.21 | 1.79 |  |  |  |  |  |
| 3q26.33 | 26433762 | Eating disorder in bipolar disorder | 3 | rs1805576-G | 0.21 | 1.71 |  |  |  |  |  |
| 3q26.33 | 26198764 | Schizophrenia | 3 | rs1805203-G | NR | 1.09 |  |  |  |  |  |
| 3q26.33 | 26433762 | Bipolar disorder and eating disorder | 3 | rs4854912-T | 0.19 | 1.91 |  |  |  |  |  |
| 3q26.33 | 26433762 | Eating disorder in bipolar disorder | 3 | rs13086738-G | 0.20 | 1.84 |  |  |  |  |  |
| 3q26.33 | 28991256 | Schizophrenia | 3 | rs9859557-A | NR | 1.09 |  |  |  |  |  |
| 3q26.33 | 26198764 | Schizophrenia | 3 | rs1878874-T | NR | 1.06 |  |  |  |  |  |
| 3q26.33 | 25056061 | Schizophrenia | 3 | rs9841616-T | 0.83 | 1.08 |  |  |  |  |  |
| 3q26.33 | 26198764 | Schizophrenia | 3 | rs9841616-T | NR | 1.09 |  |  |  |  |  |
| 3q26.33 | 28991256 | Schizophrenia | 3 | rs9841616-T | NR | 1.08 |  |  |  |  |  |
| 3q26.33 | 27126917 | Night sleep phenotypes | 3 | rs10937060-C | NR | 0.25 |  |  |  |  |  |
| 3q26.33 | 24514567 | Anorexia nervosa | 3 | rs9839776-T | 0.27 | 1.16 |  |  |  |  |  |
| 6p22.1 | 19571808 | Schizophrenia | 6 | rs6932590-T | 0.78 | 1.16 | C | 0.27 | -0.03 | 0.01 | 3.76E-07 |
| 6p22.1 | 26198764 | Schizophrenia | 6 | rs13217239-T | NR | 1.09 |  |  |  |  |  |
| 6p22.1 | 28540026 | Autism spectrum disorder or schizophrenia | 6 | rs7746199-? |  | 1.14 | T | 0.19 | -0.03 | 0.01 | 4.20E-06 |
| 6p22.1 | 23894747 | Schizophrenia | 6 | rs16897515-C | 0.16 | 1.30 | A | 0.19 | -0.03 | 0.01 | 7.35E-06 |
| 6p22.1 | 28540026 | Autism spectrum disorder or schizophrenia | 6 | rs13212562-A |  | 1.14 |  |  |  |  |  |
| 6p22.1 | 28540026 | Autism spectrum disorder or schizophrenia | 6 | rs764284-A |  | 1.07 |  |  |  |  |  |
| 6p22.1 | 28115744 | Bipolar disorder | 6 | rs9393813-A | NR | 1.09 |  |  |  |  |  |
| 6p22.1 | 28115744 | Bipolar disorder | 6 | rs2205829-G | NR | 1.09 |  |  |  |  |  |
| 6p22.1 | 28540026 | Autism spectrum disorder or schizophrenia | 6 | rs141342723-T |  | 1.20 |  |  |  |  |  |
| 6p22.1 | 27089181 | Neuroticism | 6 | rs9468186-A | 0.79 | 0.02 |  |  |  |  |  |
| 6p22.1 | 27182965 | Parkinson's disease | 6 | rs4713118-? | NR | 1.12 |  |  |  |  |  |
| 6p22.1 | 28892059 | Parkinson's disease | 6 | rs9468199-A | 0.17 | 1.11 |  |  |  |  |  |
| 6p22.1 | 28540026 | Autism spectrum disorder or schizophrenia | 6 | rs6940116-A |  | 1.11 |  |  |  |  |  |
| 6p22.1 | 22688191 | Schizophrenia | 6 | rs17693963-? | NR | 1.24 |  |  |  |  |  |
| 6p22.1 | 24166486 | Schizophrenia, schizoaffective disorder or bipolar disorder | 6 | rs17693963-? | 0.90 |  |  |  |  |  |  |
| 6p22.1 | 24280982 | Schizophrenia or bipolar disorder | 6 | rs17693963-? |  |  |  |  |  |  |  |
| 6p22.1 | 24280982 | Schizophrenia or bipolar disorder | 6 | rs17693963-? | NR |  |  |  |  |  |  |
| 6p22.1 | 26198764 | Schizophrenia | 6 | rs34706883-A | NR | 1.24 |  |  |  |  |  |
| 6p22.1 | 28540026 | Autism spectrum disorder or schizophrenia | 6 | rs200986-? |  | 1.10 |  |  |  |  |  |
| 6p22.1 | 28540026 | Autism spectrum disorder or schizophrenia | 6 | rs202906-T |  | 1.13 |  |  |  |  |  |
| 6p22.1 | 28540026 | Autism spectrum disorder or schizophrenia | 6 | rs182087722-A |  | 1.09 |  |  |  |  |  |
| 6p22.1 | 28892059 | Parkinson's disease | 6 | rs17767294-G | NR | 1.11 |  |  |  |  |  |
| 6p22.1 | 28540026 | Autism spectrum disorder or schizophrenia | 6 | rs1150688-T |  | 1.07 |  |  |  |  |  |
| 6p22.1 | 28540026 | Autism spectrum disorder or schizophrenia | 6 | rs34787248-? |  | 1.09 | T | 0.20 | -0.03 | 0.01 | 7.41E-06 |
| 6p22.1 | 22037552 | Schizophrenia | 6 | rs1635-? | NR | 1.28 |  |  |  |  |  |
| 6p22.1 | 27089181 | Depression | 6 | rs853679-A | NR | 5.30 |  |  |  |  |  |
| 6p22.1 | 26198764 | Schizophrenia | 6 | rs13217619-T | NR | 1.25 |  |  |  |  |  |
| 6p22.1 | 28540026 | Autism spectrum disorder or schizophrenia | 6 | rs6921919-C |  | 1.12 |  |  |  |  |  |
| 6p22.1 | 28115744 | Bipolar disorder | 6 | rs115769866-A | NR | 1.19 |  |  |  |  |  |
| 6p22.1 | 28540026 | Autism spectrum disorder or schizophrenia | 6 | rs116663187-A |  | 1.11 |  |  |  |  |  |
| 6p22.1 | 28540026 | Autism spectrum disorder or schizophrenia | 6 | rs144911693-? |  | 1.09 |  |  |  |  |  |
| 6p22.1 | 28540026 | Autism spectrum disorder or schizophrenia | 6 | rs116137698-A |  | 1.22 |  |  |  |  |  |
| 6p22.1 | 28078323 | Cognitive decline (age-related) | 6 | rs6939297--? |  | 0.01 |  |  |  |  |  |
| 6p22.1 | 25056061 | Schizophrenia | 6 | rs115329265-A | 0.85 | 1.21 |  |  |  |  |  |
| 6p22.1 | 28540026 | Autism spectrum disorder or schizophrenia | 6 | rs115329265-A |  | 1.17 |  |  |  |  |  |
| 6p22.1 | 28991256 | Schizophrenia | 6 | rs115329265-A | NR | 1.21 |  |  |  |  |  |
| 6p22.1 | 27089181 | Neuroticism | 6 | rs114304113-C | 0.05 | 0.05 |  |  |  |  |  |
| 6p22.1 | 27126917 | Daytime sleep phenotypes | 6 | rs150548387-G | NR | 2.54 |  |  |  |  |  |
| 6p22.1 | 27126917 | Daytime sleep phenotypes | 6 | rs114683528-T | NR | 2.44 |  |  |  |  |  |
| 6p22.1 | 27126917 | Daytime sleep phenotypes | 6 | rs116330539-G | NR | 2.32 |  |  |  |  |  |
| 6p22.1 | 27089181 | Subjective well-being | 6 | rs144077837-A | 0.05 | 0.03 |  |  |  |  |  |
| 6p22.1 | 28540026 | Autism spectrum disorder or schizophrenia | 6 | rs144649399-? |  | 1.11 |  |  |  |  |  |
| 6p22.1 | 24047820 | Social communication problems | 6 | rs9257616-G | 0.56 | 0.09 |  |  |  |  |  |
| 6p22.1 | 27126917 | Daytime sleep phenotypes | 6 | rs114080364-G | NR | 2.33 |  |  |  |  |  |
| 6p22.1 | 26198764 | Schizophrenia | 6 | rs144447022-G | NR | 1.25 |  |  |  |  |  |
| 6p22.1 | 28540026 | Autism spectrum disorder or schizophrenia | 6 | rs115661163-T |  | 1.09 |  |  |  |  |  |
| 6p22.1 | 28540026 | Autism spectrum disorder or schizophrenia | 6 | rs115937317-C |  | 1.09 |  |  |  |  |  |
| 6p22.1 | 28540026 | Autism spectrum disorder or schizophrenia | 6 | rs145501595-? |  | 1.09 |  |  |  |  |  |
| 6p22.1 | 28540026 | Autism spectrum disorder or schizophrenia | 6 | rs115123779-? |  | 1.15 |  |  |  |  |  |
| 6p22.1 | 27922604 | Schizophrenia | 6 | rs115070292-? |  | 1.30 |  |  |  |  |  |
| 6p22.1 | 28440896 | Nicotine dependence | 6 | rs56020557-T |  | 0.05 |  |  |  |  |  |
| 6p22.1 | 28440896 | Nicotine dependence | 6 | rs62392942-T |  | 0.06 |  |  |  |  |  |
| 6p22.1 | 23793025 | Migraine without aura | 6 | rs3095267-? | 0.81 | 1.12 |  |  |  |  |  |
| 6p22.1 | 28540026 | Autism spectrum disorder or schizophrenia | 6 | rs114041423-? |  | 1.11 |  |  |  |  |  |
| 6p22.1 | 26198764 | Schizophrenia | 6 | rs385492-T | NR | 1.06 |  |  |  |  |  |
| 6p22.1 | 28540026 | Autism spectrum disorder or schizophrenia | 6 | rs385492-T |  | 1.06 |  |  |  |  |  |
| 6p22.1 | 26198764 | Schizophrenia | 6 | rs3131888-C | NR | 1.28 |  |  |  |  |  |
| 7q31.1 | 28991256 | Schizophrenia | 7 | rs11534004-G | NR | 1.12 |  |  |  |  |  |
| 7q31.1 | 27225129 | Educational attainment (years of education) | 7 | rs11771168-T | 0.24 | 0.01 |  |  |  |  |  |
| 7q31.1 | 25189868 | Blood pressure (smoking interaction) | 7 | rs12705959-? | NR |  |  |  |  |  |  |
| 7q31.1 | 25189868 | Blood pressure (smoking interaction) | 7 | rs12705959-? | NR |  |  |  |  |  |  |
| 7q31.1 | 27992416 | Sleep duration | 7 | rs10953765-G | 0.45 | 0.02 | G | 0.45 | -0.03 | 0.01 | 2.55E-06 |
| 8q13.1 | 23251661 | Obesity-related traits | 8 | rs6981992-A | 0.49 | 0.02 |  |  |  |  |  |
| 8q13.1 | 26220383 | Epilepsy and lamotrigine-induced maculopapular eruptions | 8 | rs150435906-? | 0.01 | 8.60 |  |  |  |  |  |
| 10p14 | 23251661 | Obesity-related traits | 10 | rs4749791-A | 0.47 | 0.03 |  |  |  |  |  |
| 10p14 | 23793025 | Migraine | 10 | rs827382-C | 0.25 | 1.07 |  |  |  |  |  |
| 10p14 | 29221444 | Coronary artery calcified atherosclerotic plaque (90 or 130 HU threshold) in type 2 diabetes | 10 | rs114408555-? | NR | 0.22 |  |  |  |  |  |
| 10p14 | 25656473 | Clozapine-induced cytotoxicity | 10 | rs1149933-? | NR |  |  |  |  |  |  |
| 10p14 | 26089329 | Stroke | 10 | rs768606-A | 0.07 | 1.62 |  |  |  |  |  |
| 10p14 | 26089329 | Stroke | 10 | rs768606-A | 0.07 | 0.48 |  |  |  |  |  |
| 11q22.3 | 26198764 | Schizophrenia | 11 | rs1144403-A | NR | 1.05 |  |  |  |  |  |
| 11q22.3 | 27770636 | Late-onset Alzheimer's disease | 11 | rs79930850-A | 0.99 | 0.45 |  |  |  |  |  |
| 12p12.1 | 26112879 | LDL peak particle diameter (total fat intake interaction) | 12 | rs16926789-A | 0.89 | 0.05 |  |  |  |  |  |
| 12p12.1 | 25673413 | Body mass index | 12 | rs7970953-A | 0.30 | 0.02 |  |  |  |  |  |
| 12p12.1 | 26198764 | Schizophrenia | 12 | rs7314326-A | NR | 1.06 |  |  |  |  |  |
| 12p12.1 | 20195266 | Response to antipsychotic treatment | 12 | rs1464500-? | 0.23 |  |  |  |  |  |  |
| 12p12.1 | 27798624 | Resting heart rate | 12 | rs4963772-A | 0.15 | 0.71 |  |  |  |  |  |
| 12p12.1 | 28135244 | Pulse pressure | 12 | rs4963772-A | 0.15 | 0.21 |  |  |  |  |  |
| 12p12.1 | 28610988 | Heart rate variability traits (RMSSD) | 12 | rs4963772-A | 0.13 | 0.07 |  |  |  |  |  |
| 12p12.1 | 20639392 | Resting heart rate | 12 | rs17287293-G | 0.15 | 8.60 |  |  |  |  |  |
| 12p12.1 | 23583979 | Heart rate | 12 | rs17287293-A | 0.85 | 0.44 |  |  |  |  |  |
| 12p12.1 | 28613276 | Heart rate variability traits (SDNN) | 12 | rs10842383-C | 0.86 | 0.05 |  |  |  |  |  |
| 12p12.1 | 28613276 | Heart rate variability traits (RMSSD) | 12 | rs10842383-C | 0.86 | 0.06 |  |  |  |  |  |
| 12p12.1 | 28613276 | Heart rate variability traits (pvRSA/HF) | 12 | rs10842383-C | 0.87 | 0.12 |  |  |  |  |  |
| 12p12.1 | 29127183 | PR interval | 12 | rs146974314-A | 0.14 | 2.66 |  |  |  |  |  |
| 12p12.1 | 20062060 | PR interval | 12 | rs11047543-A | 0.15 | 2.09 |  |  |  |  |  |
| 15q24.1 | 22001757 | Liver enzyme levels (gamma-glutamyl transferase) | 15 | rs8038465-T | 0.39 | 2.40 |  |  |  |  |  |
| 15q24.1 | 21738484 | Bipolar disorder | 15 | rs1038094-G | NR | 1.27 |  |  |  |  |  |
| 15q24.1 | 25673412 | Waist circumference adjusted for body mass index | 15 | rs4886782-A | 0.37 | 0.03 |  |  |  |  |  |
| 15q24.1 | 28443625 | Waist circumference adjusted for BMI (adjusted for smoking behaviour) | 15 | rs4886782-A | 0.36 | 0.03 |  |  |  |  |  |
| 15q24.1 | 28443625 | Waist circumference adjusted for BMI in smokers | 15 | rs4886782-A | 0.36 | 0.06 |  |  |  |  |  |
| 15q24.1 | 28448500 | Waist circumference adjusted for BMI (joint analysis main effects and physical activity interaction) | 15 | rs4886782-? | 0.62 |  |  |  |  |  |  |
| 15q24.1 | 28448500 | Waist circumference adjusted for body mass index | 15 | rs4886782-G | 0.63 | 0.04 |  |  |  |  |  |
| 15q24.1 | 19584346 | Aortic root size | 15 | rs893817-G | 0.34 | 0.02 |  |  |  |  |  |
| 15q24.1 | 28135244 | Diastolic blood pressure | 15 | rs56007454-C | 0.03 | 0.37 |  |  |  |  |  |
| 15q24.1 | 23251661 | Obesity-related traits | 15 | rs9783698-A | 0.15 | 0.04 |  |  |  |  |  |
| 16q22.1 | 18821565 | Attention deficit hyperactivity disorder | 16 | rs8047014-? | NR |  |  |  |  |  |  |
| 16q22.1 | 25189868 | Blood pressure (smoking interaction) | 16 | rs12149862-? | NR |  |  |  |  |  |  |
| 16q22.1 | 24204828 | Homeostasis model assessment of beta-cell function (dietary factor interaction) | 16 | rs744972-C | 0.26 |  |  |  |  |  |  |
| 16q22.1 | 25673413 | Body mass index | 16 | rs889398-C | 0.58 | 0.01 |  |  |  |  |  |
| 16q22.1 | 28135244 | Pulse pressure | 16 | rs141767645-T | 0.02 | 1.30 |  |  |  |  |  |
| Where: RA, risk allele; RAF, risk allele frequency | | | | | | | | | | | |
