# Supplementary material for: Genetics of self-reported risk-taking behaviour, trans-ethnic consistency and relevance to brain gene expression
Source: Transl Psychiatry. 2018 Sep 4;8:178. doi: 10.1038/s41398-018-0236-1 (PMC6123450; doi:10.1038/s41398-018-0236-1)
Supplement: Supplementary file 27 — Supplementary Table 20 [file 41398_2018_236_MOESM27_ESM.docx]

Supplementary Table 20: Conditional analyses of lead risk-taking SNPs

|  |  |  | LD r2 |  | oringinal analysis | | conditional analyses | | | | | | | | |  |
| --- | --- | --- | --- | --- | --- | --- | --- | --- | --- | --- | --- | --- | --- | --- | --- | --- |
| CHR | SNP | BP |  | A1 | BETA | P | BETA | P | BETA | P | BETA | P | BETA | P | |  |
| 1 | rs58560561^c^ | 243537729 | 0 | T |  | | -0.020 | 1.12E-03 |  | |  | | | | |  |
|  | rs560977020 | 243812368 |  | C | -0.035 | **2.04E-08** |  | | -0.031 | 1.58E-06 |  |  |  |  |  |  |
| 2 | rs2304681 | 27315252 | 0.7 | A | -0.033 | **2.58E-08** | -0.018 | 1.14E-01 |  | |  | | | | |  |
|  | rs12617392^c^ | 27336827 |  | A |  | | | | -0.016 | 1.49E-01 |  |  |  |  |  |  |
| 3 | rs6762267^c^ | 85513115 | 0.8 | C |  | | | | 0.028 | 6.05E-02 |  | | | | |  |
|  | rs542809491 | 85617378 |  | A | 0.056 | **1.02E-19** | 0.031 | 3.82E-02 |  | |  |  |  |  |  |  |
| 6 | rs188973463 | 27766842 | 0.2 | G | -0.042 | **5.16E-10** | -0.033 | 1.08E-05 |  | | -0.031 | 8.31E-05 | -0.031 | 7.24E-05 | |  |
|  | rs6923811^c^ | 27289776 |  | C |  | |  | | -0.021 | 3.04E-03 | -0.026 | 9.27E-05 | -0.025 | 1.54E-04 | |  |
| 6 | rs566858049 | 29230129 | 1 | C | -0.036 | **2.00E-09** | -0.028 | 8.06E-06 | -0.022 | 1.58E-03 |  | | -0.029 | 2.80E-01 | |  |
|  | rs3117340^c^ | 29210596 |  | T |  | | -0.027 | 1.41E-05 | -0.020 | 3.47E-03 | -0.007 | 7.92E-01 |  | | |  |
| 7 | rs727644^ab^ | 114109349 | 0 | A | -0.036 | **8.99E-10** |  | | -0.034 | **1.31E-08** |  | | | | |  |
| 7 | rs1358391c | 115111838 |  |  |  | | -0.023 | 8.70E-05 |  |  |  |  |  |  |  |  |
| 8 | rs62519827^c^ | 65481947 | 1 | C |  | |  | | | | | | | | |  |
|  | rs189335278 | 65508415 |  | A | -0.053 | **1.03E-08** |  |  |  |  |  |  |  |  |  |  |
| 11 | 11:104700736 | 104700736 | 0.3 | A | 0.039 | **7.59E-09** | 0.026 | 1.66E-03 |  | |  | | | | |  |
|  | rs11226319^c^ | 104221573 |  | A |  | | | | 0.031 | 1.07E-03 |  |  |  |  |  |  |
| 16 | rs145206681 | 69550486 | 0 | T | 0.067 | **2.47E-08** | 0.064 | 1.44E-07 |  | |  | | | | |  |
| 16 | rs891124^c^ | 71440756 |  | C |  | | | | -0.030 | 3.14E-06 |  |  |  |  |  |  |
| Where: ^a^ a proxy with an rsid, used for data-mining; ^b^ same lead SNP as Clifton et al; ^c^ indicates Clifton et al lead SNPs | | | | | | | | | | | | | | |  | |
