# Supplementary material for: Genetics of self-reported risk-taking behaviour, trans-ethnic consistency and relevance to brain gene expression
Source: Transl Psychiatry. 2018 Sep 4;8:178. doi: 10.1038/s41398-018-0236-1 (PMC6123450; doi:10.1038/s41398-018-0236-1)
Supplement: Supplementary file 28 — Supplemental Table 21 [file 41398_2018_236_MOESM28_ESM.docx]

Supplementary Table 21: transethnic meta-analysis of clifton et al lead SNPs

| CHR | MarkerName | BP | A1 | A2 | Freq1 | FreqSE | Effect | StdErr | P-value | Direction | HetISq | HetChiSq | HetDf | HetPVal |
| --- | --- | --- | --- | --- | --- | --- | --- | --- | --- | --- | --- | --- | --- | --- |
| 1 | rs4653015 | 33776431 | t | c | 0.27 | 0.04 | 0.032 | 0.006 | 2.29E-07 | ++--+ | 73.5 | 15.108 | 4 | **0.0045** |
| 1 | rs4233093 | 73446245 | a | g | 0.51 | 0.04 | 0.030 | 0.005 | 3.17E-08 | ++++- | 59.3 | 9.839 | 4 | **0.0432** |
| 1 | rs58560561^a^ | 243537729 | t | g | 0.36 | 0.05 | -0.027 | 0.005 | 4.25E-07 | --0+- | 0 | 3.938 | 4 | 0.4144 |
| 2 | rs12617392^a^ | 27336827 | a | c | 0.45 | 0.04 | -0.030 | 0.005 | 1.84E-08 | --+-- | 0 | 3.224 | 4 | 0.5211 |
| 2 | rs542883 | 45143382 | c | g | 0.57 | 0.05 | 0.029 | 0.005 | 8.60E-08 | ++++- | 0 | 1.652 | 4 | 0.7994 |
| 2 | rs12476923 | 145830053 | a | c | 0.33 | 0.02 | 0.023 | 0.005 | 2.26E-05 | ++++- | 54 | 8.692 | 4 | 0.0693 |
| 3 | rs283914 | 17330649 | t | c | 0.54 | 0.03 | 0.029 | 0.005 | 4.25E-08 | +++-+ | 32.4 | 5.921 | 4 | 0.2052 |
| 3 | rs6762267^a^ | 85513115 | a | c | 0.61 | 0.05 | -0.056 | 0.005 | 3.90E-25 | ---+- | 22.6 | 5.165 | 4 | 0.2708 |
| 3 | rs9841382^a^ | 181408124 | t | c | 0.84 | 0.08 | -0.052 | 0.007 | 7.40E-13 | ----- | 41.4 | 6.824 | 4 | 0.1455 |
| 4 | rs992493 | 106180264 | t | c | 0.19 | 0.02 | 0.034 | 0.006 | 4.23E-08 | ++--- | 0 | 2.916 | 4 | 0.5720 |
| 6 | rs6923811^a^ | 27289776 | t | c | 0.67 | 0.03 | 0.032 | 0.005 | 4.61E-09 | ++-++ | 0 | 2.78 | 4 | 0.5952 |
| 6 | rs3117340^a^ | 29210596 | t | g | 0.38 | 0.04 | -0.030 | 0.005 | 1.33E-08 | ---+- | 0 | 3.432 | 4 | 0.4883 |
| 6 | rs34905321 | 109131107 | t | c | 0.57 | 0.02 | 0.020 | 0.005 | 0.000154 | ++++- | 0 | 2.54 | 4 | 0.6374 |
| 7 | rs727644^a^ | 114109349 | a | g | 0.40 | 0.03 | -0.038 | 0.005 | 2.02E-12 | ----- | 0 | 2.55 | 4 | 0.6357 |
| 7 | rs1358391 | 115111838 | t | g | 0.49 | 0.03 | -0.026 | 0.005 | 1.33E-06 | ----- | 0 | 3.926 | 4 | 0.4161 |
| 8 | rs7829912 | 33479228 | t | c | 0.56 | 0.03 | 0.031 | 0.005 | 7.43E-09 | +++++ | 32.3 | 5.912 | 4 | 0.2058 |
| 8 | rs62519827^a^ | 65481947 | t | c | 0.89 | 0.01 | 0.051 | 0.008 | 7.67E-10 | ++++- | 0 | 1.89 | 4 | 0.7560 |
| 8 | rs7817124 | 81404008 | c | g | 0.26 | 0.08 | 0.032 | 0.006 | 2.38E-07 | +++++ | 0 | 0.889 | 4 | 0.9262 |
| 9 | rs12115650 | 126367705 | a | g | 0.27 | 0.02 | -0.032 | 0.006 | 1.91E-07 | ---+- | 0 | 1.672 | 4 | 0.7958 |
| 10 | rs10823791 | 73338334 | a | t | 0.60 | 0.01 | -0.028 | 0.005 | 1.21E-07 | ----- | 0 | 0.405 | 4 | 0.9821 |
| 11 | rs11226319^a^ | 104221573 | a | g | 0.16 | 0.02 | 0.036 | 0.007 | 3.70E-07 | +++-- | 71.4 | 13.989 | 4 | **0.0073** |
| 14 | rs35914833 | 94182383 | t | c | 0.32 | 0.03 | -0.028 | 0.005 | 2.82E-07 | --++- | 0 | 2.854 | 4 | 0.5825 |
| 14 | rs1381287 | 98597552 | t | c | 0.45 | 0.02 | 0.025 | 0.005 | 2.54E-06 | +++++ | 0 | 0.552 | 4 | 0.9683 |
| 16 | rs891124^a^ | 71440756 | t | c | 0.71 | 0.01 | 0.029 | 0.005 | 9.23E-08 | +++-- | 37.9 | 6.439 | 4 | 0.1687 |
| 18 | rs4801000 | 53456943 | a | g | 0.66 | 0.02 | -0.029 | 0.005 | 1.09E-07 | ----- | 57.7 | 9.462 | 4 | 0.0505 |
| 22 | rs28520003 | 46411969 | a | g | 0.32 | 0.04 | -0.026 | 0.005 | 1.34E-06 | --+++ | 71.4 | 13.987 | 4 | **0.0073** |
| Where: bold indicatesnominal (p<0.05) significance; ^a^, loci identified in the current study. | | | | | | | | | | | | | | |
